# An updated list of the genus *Hypena* Schrank (Lepidoptera, Erebidae) from Korea with five additional records to the fauna

**DOI:** 10.3897/BDJ.13.e155581

**Published:** 2025-05-02

**Authors:** Dahee Jin, Sung-Soo Kim, Bora Shin, Sei-Woong Choi

**Affiliations:** 1 Mokpo National University, Muan, Republic of Korea Mokpo National University Muan Republic of Korea; 2 Research Institute for East Asian Environment and Biology, Seoul, Republic of Korea Research Institute for East Asian Environment and Biology Seoul Republic of Korea

**Keywords:** checklist, external morphology, genitalia, DNA barcoding, phylogeny

## Abstract

**Background:**

The paper provides the updated checklist of the genus *Hypena* Schrank from Korea. This genus is one of the largest genera within the Noctuoidea comprising more than 680 species worldwide and the genus is the monophyletic group based on the morphological characters. The external examination along with the genitalia examination and DNA barcoding could reveal the diversity of the genus in Korea.

**New information:**

In this study, we examined a total of 192 specimens and barcoded 16 species and listed a total of 29 species of *Hypena* including five new additions, *Hypenatamsi* Filipjev (1927), *Hypenaobacerralis* Walker (1859), *Hypenapulverulenta* Wileman (1911), *Hypenaperspicua* Leech (1900) and *Hypenamandarina* Leech (1900) to the Korean fauna. We provided the detailed distribution of each species of the genus across South Korea and the photographs of adults and genitalia. In addition, the monophyly of the genus was also confirmed using two outgroup species of Herminiinae. This study significantly contributes to the knowledge of erebid fauna in Korea and the phylogenetic relationship amongst the species of the genus.

## Introduction

The genus *Hypena* Schrank (1802), one of the largest genera within the Noctuoidea, comprises more than 680 species worldwide and is predominant in the Tropics (Lödl 1994). The genus is monophyletic and can be recognised by the morphological characters such as ciliate male antennae, the elongated and obliquely porrect labial palps, the unified ground plan of the wing pattern elements with a prominent postmedial line of forewing and a uniform hindwing, a firm and coarse corium on the dorsal surface of the tuba analis and the hooked and apically acute uncus and the simple valva with a short longitudinal pleat centrally over the basal part (= clavus) in the male genitalia and the simple ostium bursae and the long slender ductus bursae in the female genitalia (Lödl 1994, Lödl 2000, Holloway 2008).

In Korea, the genus was first recorded by Bryk (1949) who recorded five species. Kononenko et al. (1998) listed 22 species of the genus. Recently, Lee et al. (2022) reported *Hypenanarratalis* Walker (1859) from a cave in Gangwon Province. The purpose of the present paper is to report five additional species of *Hypena* for the first time in Korea and provide the updated checklist of the genus in Korea with a few notes on the records.

## Materials and methods

### Specimen preparation

We examined a total of 194 specimens collected from South Korea. Materials were collected using an ultraviolet bucket trap and are now deposited in the Collection of Insects at Mokpo National University (MNU). The collected adults were preserved in a freezer and mounted for examination. Species identification was mainly based on the external morphology of adults including wing pattern elements and genitalia. For genitalia slide preparation, the specimen was prepared for 15-20 min. The scales and tissues were removed, stained with Chlorazol black and mounted on slides in Euparal mounting medium. Photographs of the adults were taken using a Nikon D300 DSLR camera equipped with an AF-S Micro-Nikkor 105 mm f/2.8G IF-ED objective and genitalia were photographed with the built-in digital camera of a Leica S9i stereomicroscope.

The terminology of the adult characteristics, including the genitalia, refers to Lödl (1993). Abbreviations are as follows: GW, Gwangwon-do, GG, Gyunggi-do, GB, Gyungsangbuk-do, GN, Gyungsangnam-do, CB, Chungchungbuk-do, CN, Chungchungnam-do, JB, Jeollabuk-do, JN, Jeollanam-do, JJ, Jeju-do; Gen., Genitalia preparation.

### DNA preparation

To examine genetic differences amongst related species, genomic DNA was extracted using the Exgene™ Tissue SV mini kit (GeneAll Biotechnology Co., Ltd., Korea). PCR amplification of a 658 bp segment of the mitochondrial COI gene was performed using a T100™ Thermal Cycler (BioRad, USA), the primers LCO1490 and HCO2198 (Folmer et al. 1994) and GainBlue™ HOT Start Pro Premix, 2x (GainBio, Daejeon, Korea). The PCR procedure adhered to the method outlined by Choi et al. (2021). Purification of the PCR products was carried out using the ExoSAP-IT™ PCR Product Cleanup Reagent (Applied Biosystems, USA) and the samples were sent to Bioneer Co., Ltd. (Daejeon, South Korea) for sequencing. Sequence editing and alignment were conducted using MEGA 11 (Tamura et al. 2021). The resulting COI sequences were identified via NCBI (National Center for Biotechnology Information) and utilised to determine genetic differences amongst species within the genus.

### DNA barcoding and phylogeny

Genetic distance and Maximum Likelihood (ML) analyses were carried out using 35 COI sequences obtained from the current study (16 sequences) and NCBI GenBank database (19 sequences, https://www.ncbi.nlm.nih.gov/). Sequence alignment was completed in MEGA 11 (Tamura et al. 2021) using the MUSCLE algorithm (Edgar 2004) under default parameters. Genetic distances were calculated, based on the Kimura 2-parameter model (Kimura 1980) using MEGA 11. ML analysis was performed in IQ-tree version 1.6.2 (Nguyen et al. 2015). An initial model test in IQ-tree determined the optimal model for the dataset and tree robustness was assessed via 1,000 bootstrap replicates. Details, including voucher/specimen IDs and GenBank accession/sequence IDs for the COI barcodes, can be found in the Suppl. material 1.

## Checklists

### Checklist of the genus Hypena Schrank in Korea

#### Hypena

Schrank, 1802

D99E9EE3-0730-5039-AAD7-897F2B3CA3EB

Ameltropalpis Mabille, 1884 - Mabille (1884) | *Anepischetos* Smith, 1900 - Smith (1900) | *Apanda* Moore, 1882 - Moore (1882) | *Badausa* Walker, 1863 - Walker (1863) | *Bomolocha* Hübner, 1825 - Hübner (1825) | *Camhypena* Prout, 1927 - Prout (1927) | *Dichromia* Guenée, 1854 - Guenée (1854) | *Erichila* Billberg, 1820 -Billberg (1820) | *Eugrona* Holland, 1894 - Holland (1894) | *Euhyphena* Grote, 1873 - Grote (1873c) | *Harita* Moore, 1882 - Moore (1882) | *Herpyzon* Hübner, 1822 - Hübner (1822) | *Lomanaltes* Grote, 1873 - Grote (1873a) | *Macrhypena* Grotte, 1873 - Grote (1873c) | *Mathura* Moore, 1882 -Moore (1882) | *Meghypena* Grote, 1873 - Grote (1873b) | *Nesamiptis* Meyrick, 1899 - Meyrick (1899) | *Ophiuche* Hübner, 1825 - Hübner (1825)| *Peliala* Walker, 1865 - Walker (1865) | *Plathypena* Grote, 1873 - Grote (1873c) | *Placerobela* Turner, 1903 -Turner (1903).

##### Notes

TS: *Phalaenaproboscidalis* Linnaeus, 1758. TL: Europe.

##### Diagnosis

Antennae minutely ciliated in male; labial palpi large, roughly scaled, the second segment long and porrect, strongly projected beyond frons; frons with long projected tufts. Thorax ventrally with long hairs. Forewing ground colour greyish, brownish or light blackish; medial fascia distinct with slanted postmedial lines; apex depressed, slightly acute; areole present. Hindwings ground colour yellowish or slightly darker than forewing; areole formed between veins 3 and 4; discal dot present. Male genitalia. Uncus long, hooked. Valva with distinct clavus; costa slightly sclerotised, curved or straight. Aedeagus with a bundle of cornuti on globular vesica. Female genitalia. Papillae analis broad; anterior apophyses often long, more than twice the posterior apophyses. Ostium broad, cup-shaped, without sclerotised appendages; ductus bursae broad and shrunken or slender; corpus bursae large, globular without a signum.

#### Hypena sagitta

(Fabricius, 1775)

1DCA9D6A-2FFE-51EE-BE8D-CC68CA91DAA3

http://en.wikipedia.org/wiki/Atypus_affinis

##### Materials

- **Type status:** Other material. **Occurrence:** recordedBy: Choi, Sei-Woong; individualCount: 1; sex: female; lifeStage: adult; disposition: Mokpo National University; occurrenceID: 4FB1DF10-1F71-55BD-8955-7DC064BCFA33; **Taxon:** scientificName: *Hypenasagitta*; **Location:** country: South Korea; stateProvince: GW; county: Samcheok; locality: Dongmak-ri, Geundeok-myeon; **Identification:** identifiedBy: Sung-Soo Kim; dateIdentified: 2024; **Event:** samplingProtocol: Ultraviolet bucket trap; samplingEffort: 6 trapping hours; eventDate: 8/22/2023; **Record Level:** modified: 3/30/2025; language: en; collectionCode: Insects; basisOfRecord: PreservedSpecimen
- **Type status:** Other material. **Occurrence:** recordedBy: Choi, Sei-Woong; individualCount: 1; sex: male; lifeStage: adult; disposition: Mokpo National University; occurrenceID: 18A922E3-63B3-5D62-9D20-B50CE28EBFDD; **Taxon:** scientificName: *Hypenasagitta*; **Location:** country: South Korea; stateProvince: GN; county: Sancheong; locality: Yulhayeon-ri, Sindeung-myeon; verbatimElevation: 322; verbatimCoordinates: 35°24'45.63"N 127°58'13.9"E; **Identification:** identifiedBy: Sei-Woong Choi; dateIdentified: 2024; **Event:** samplingProtocol: Ultraviolet bucket trap; samplingEffort: 6 trapping hours; eventDate: 5/24/2019; **Record Level:** modified: 3/30/2025; language: en; collectionCode: Insects; basisOfRecord: PreservedSpecimen

##### Distribution

Korea, Japan, Taiwan, Hong Kong, Macau, India.

##### Notes

Sugi (1989) first recorded this species in Korea without the locality data. This species is rare and we only examined two specimens in this study (Figs 1a, b, 7a, b).

#### Hypena claripennis

Butler, 1878

55790AD6-C142-5041-A45A-D70F84121706

##### Materials

- **Type status:** Other material. **Occurrence:** recordedBy: Choi, Sei-Woong; individualCount: 1; sex: male; lifeStage: adult; disposition: Mokpo National University; occurrenceID: 2AE4EF52-2B52-5B06-BD8F-651DEC0C47FD; **Taxon:** scientificName: *Hypenaclaripennis*; **Location:** country: South Korea; stateProvince: JN; county: Shinan; locality: Osang-ri, Amtae-myeon; verbatimElevation: 150; verbatimCoordinates: 34°50'30"N 126°4'38"E; **Identification:** identifiedBy: Sei-Woong Choi; dateIdentified: 2024; **Event:** samplingProtocol: Ultraviolet bucket trap; samplingEffort: 6 trapping hours; eventDate: 2023/08/24; **Record Level:** modified: 3/30/2025; language: en; collectionCode: Insects; basisOfRecord: PreservedSpecimen
- **Type status:** Other material. **Occurrence:** recordedBy: Choi, Sei-Woong; individualCount: 1; sex: female; lifeStage: adult; disposition: Mokpo National University; occurrenceID: FF4C7722-2124-51B9-9901-0DD1678C4212; **Taxon:** scientificName: *Hypenaclaripennis*; **Location:** country: South Korea; stateProvince: JN; county: Shinan; locality: Osang-ri, Amtae-myeon; verbatimElevation: 150; verbatimCoordinates: 34°50'30"N 126°4'38"E; **Identification:** identifiedBy: Sei-Woong Choi; dateIdentified: 2024; **Event:** samplingProtocol: Ultraviolet bucket trap; samplingEffort: 6 trapping hours; eventDate: 2023/08/24; **Record Level:** modified: 3/30/2025; language: en; collectionCode: Insects; basisOfRecord: PreservedSpecimen
- **Type status:** Other material. **Occurrence:** recordedBy: Choi, Sei-Woong; individualCount: 2; sex: males; lifeStage: adult; disposition: Mokpo National University; occurrenceID: 7FBE33DD-D79A-5C2F-8154-E4621458BB9F; **Taxon:** scientificName: *Hypenaclaripennis*; **Location:** country: South Korea; stateProvince: JN; county: Jindo; locality: Jodo-myeon,; **Identification:** identifiedBy: Sei-Woong Choi; dateIdentified: 2024; **Event:** samplingProtocol: Ultraviolet bucket trap; samplingEffort: 6 trapping hours; eventDate: 2008/07/28; **Record Level:** modified: 3/30/2025; language: en; collectionCode: Insects; basisOfRecord: PreservedSpecimen
- **Type status:** Other material. **Occurrence:** recordedBy: Choi, Sei-Woong; individualCount: 1; sex: male; lifeStage: adult; disposition: Mokpo National University; occurrenceID: CEF012BF-ED94-5B0D-8AAF-A206F5160D6E; **Taxon:** scientificName: *Hypenaclaripennis*; **Location:** country: South Korea; stateProvince: GN; county: Namhae; locality: Idong-myeon; **Identification:** identifiedBy: Sung-Soo Kim; dateIdentified: 2024; **Event:** samplingProtocol: Ultraviolet bucket trap; samplingEffort: 6 trapping hours; eventDate: 2014/04/08; **Record Level:** modified: 3/30/2025; language: en; collectionCode: Insects; basisOfRecord: PreservedSpecimen
- **Type status:** Other material. **Occurrence:** recordedBy: Choi, Sei-Woong; individualCount: 1; sex: male; lifeStage: adult; disposition: Mokpo National University; occurrenceID: 5D763916-13DD-5894-AC97-86339FFB9DD5; **Taxon:** scientificName: *Hypenaclaripennis*; **Location:** country: South Korea; stateProvince: JN; county: Shinan; locality: Jaeun-myeon; verbatimElevation: 84; verbatimCoordinates: 34°53'23"N 126°2'43"E; **Identification:** identifiedBy: Sei-Woong Choi; dateIdentified: 2024; **Event:** samplingProtocol: Ultraviolet bucket trap; samplingEffort: 6 trapping hours; eventDate: 2020/09/08; **Record Level:** modified: 3/30/2025; language: en; collectionCode: Insects; basisOfRecord: PreservedSpecimen
- **Type status:** Other material. **Occurrence:** recordedBy: Choi, Sei-Woong; individualCount: 1; sex: male; lifeStage: adult; disposition: Mokpo National University; occurrenceID: 3382D856-5DBB-5D52-8B68-F90D5348FD5A; **Taxon:** scientificName: *Hypenaclaripennis*; **Location:** country: South Korea; stateProvince: JN; county: Shinan; locality: Gageodo-ri, Heuksan-myeon; verbatimElevation: 160; verbatimCoordinates: 34°4'30"N 125°5'54.96"E; **Identification:** identifiedBy: Sei-Woong Choi; dateIdentified: 2024; **Event:** samplingProtocol: Ultraviolet bucket trap; samplingEffort: 6 trapping hours; eventDate: 2022/07/19; **Record Level:** modified: 3/30/2025; language: en; collectionCode: Insects; basisOfRecord: PreservedSpecimen
- **Type status:** Other material. **Occurrence:** recordedBy: Choi, Sei-Woong; individualCount: 1; sex: male; lifeStage: adult; disposition: Mokpo National University; occurrenceID: 1603AF8E-9C8C-5CE3-A78F-D87675DF9F23; **Taxon:** scientificName: *Hypenaclaripennis*; **Location:** country: South Korea; stateProvince: JN; county: Muan; locality: Cheonggye-ri, Cheonggye-myeon; verbatimElevation: 44; verbatimCoordinates: 34°54'27"N 126°25'14"E; **Identification:** identifiedBy: Sei-Woong Choi; dateIdentified: 2024; **Event:** samplingProtocol: Ultraviolet bucket trap; samplingEffort: 6 trapping hours; eventDate: 2023/09/07; **Record Level:** modified: 3/30/2025; language: en; collectionCode: Insects; basisOfRecord: PreservedSpecimen
- **Type status:** Other material. **Occurrence:** recordedBy: Choi, Sei-Woong; individualCount: 1; sex: male; lifeStage: adult; disposition: Mokpo National University; occurrenceID: 652EF959-9963-5899-865F-7F17D927D6A1; **Taxon:** scientificName: *Hypenaclaripennis*; **Location:** country: South Korea; stateProvince: JN; county: Jindo; locality: Yeomi-ri, Jodo-myeon; verbatimElevation: 170; verbatimCoordinates: 34°19'40.33"N 126°0'13.39"E; **Identification:** identifiedBy: Sei-Woong Choi; dateIdentified: 2024; **Event:** samplingProtocol: Ultraviolet bucket trap; samplingEffort: 6 trapping hours; eventDate: 2024/08/17; **Record Level:** modified: 3/30/2025; language: en; collectionCode: Insects; basisOfRecord: PreservedSpecimen
- **Type status:** Other material. **Occurrence:** recordedBy: Choi, Sei-Woong; individualCount: 1; sex: female; lifeStage: adult; disposition: Mokpo National University; occurrenceID: 55C11CFD-1E0C-5C9F-BAD4-CA6DE590CF13; **Taxon:** scientificName: *Hypenaclaripennis*; **Location:** country: South Korea; stateProvince: JB; county: Jeongeup; locality: Sucheong-ri, Chilbo-myeon; **Identification:** identifiedBy: Sung-Soo Kim; dateIdentified: 2024; **Event:** samplingProtocol: Ultraviolet bucket trap; samplingEffort: 6 trapping hours; eventDate: 2013/05/04; **Record Level:** modified: 3/30/2025; language: en; collectionCode: Insects; basisOfRecord: PreservedSpecimen
- **Type status:** Other material. **Occurrence:** recordedBy: Choi, Sei-Woong; individualCount: 1; sex: female; lifeStage: adult; disposition: Mokpo National University; occurrenceID: 1EDA6FFB-2841-5A30-A90F-9E245891B61B; **Taxon:** scientificName: *Hypenaclaripennis*; **Location:** country: South Korea; stateProvince: JN; county: Yeosu; locality: Yusong-ri, Nam-myeon; verbatimElevation: 118; verbatimCoordinates: 34°32'27"N 127°43'28"E; **Identification:** identifiedBy: Sei-Woong Choi; dateIdentified: 2024; **Event:** samplingProtocol: Ultraviolet bucket trap; samplingEffort: 6 trapping hours; eventDate: 2021/07/21; **Record Level:** modified: 3/30/2025; language: en; collectionCode: Insects; basisOfRecord: PreservedSpecimen
- **Type status:** Other material. **Occurrence:** recordedBy: Choi, Sei-Woong; individualCount: 1; sex: female; lifeStage: adult; disposition: Mokpo National University; occurrenceID: FAA5DA46-8A47-5C71-BA63-94D7F742FDC2; **Taxon:** scientificName: *Hypenaclaripennis*; **Location:** country: South Korea; stateProvince: JN; county: Yeongam; locality: Dogap-ri, Gunseo-myeon; verbatimElevation: 121; verbatimCoordinates: 34°45'17.92"N 126°39'43.79"E; **Identification:** identifiedBy: Sei-Woong Choi; dateIdentified: 2024; **Event:** samplingProtocol: Ultraviolet bucket trap; samplingEffort: 6 trapping hours; eventDate: 2023/08/20; **Record Level:** modified: 3/30/2025; language: en; collectionCode: Insects; basisOfRecord: PreservedSpecimen
- **Type status:** Other material. **Occurrence:** recordedBy: Choi, Sei-Woong; individualCount: 1; sex: female; lifeStage: adult; disposition: Mokpo National University; occurrenceID: A92B3682-E0AB-52D6-9E41-4E94160787CD; **Taxon:** scientificName: *Hypenaclaripennis*; **Location:** country: South Korea; stateProvince: JN; county: Shinan; locality: Gageodo-ri, Heuksan-myeon; verbatimElevation: 110; verbatimCoordinates: 34°4'18.98"N 125°5'39.12"E; **Identification:** identifiedBy: Sei-Woong Choi; dateIdentified: 2024; **Event:** samplingProtocol: Ultraviolet bucket trap; samplingEffort: 6 trapping hours; eventDate: 2022/07/19; **Record Level:** modified: 3/30/2025; language: en; collectionCode: Insects; basisOfRecord: PreservedSpecimen
- **Type status:** Other material. **Occurrence:** recordedBy: Choi, Sei-Woong; individualCount: 1; sex: female; lifeStage: adult; disposition: Mokpo National University; occurrenceID: 2E5535F2-89EF-50FD-A812-F48448B87ECF; **Taxon:** scientificName: *Hypenaclaripennis*; **Location:** country: South Korea; stateProvince: JN; county: Muan; locality: Cheonggye-ri, Cheonggye-myeon; verbatimElevation: 44; verbatimCoordinates: 34°54'27"N 126°25'14"E; **Identification:** identifiedBy: Sei-Woong Choi.; dateIdentified: 2024; **Event:** samplingProtocol: Ultraviolet bucket trap; samplingEffort: 6 trapping hours; eventDate: 2021/09/09; **Record Level:** modified: 3/30/2025; language: en; collectionCode: Insects; basisOfRecord: PreservedSpecimen

##### Distribution

Korea, Japan, China.

##### Notes

Fig. 1c, d.

#### Hypena amica

Butler, 1878

45A0C26C-60E7-5B5F-96A7-AD95B1947336

##### Materials

- **Type status:** Other material. **Occurrence:** recordedBy: Choi, Sei-Woong; individualCount: 1; sex: male; lifeStage: adult; disposition: Mokpo National University; occurrenceID: 7F215988-785D-5DB5-AF37-4FC70BC55E9E; **Taxon:** scientificName: *Hypenaamica*; **Location:** country: South Korea; stateProvince: GW; county: Wonju; locality: Geumdae-ri, Panbu-myeon; verbatimElevation: 543; verbatimCoordinates: 37°15'9.4"N 128°1'53.52"E; **Identification:** identifiedBy: Sei-Woong Choi.; dateIdentified: 2024; **Event:** samplingProtocol: Ultraviolet bucket trap; samplingEffort: 6 trapping hours; eventDate: 5/20/2024; **Record Level:** modified: 3/30/2025; language: en; collectionCode: Insects; basisOfRecord: PreservedSpecimen
- **Type status:** Other material. **Occurrence:** recordedBy: Choi, Sei-Woong; individualCount: 1; sex: female; lifeStage: adult; disposition: Mokpo National University; occurrenceID: 590F8CFF-16D3-5924-A9BE-8B672A9641AE; **Taxon:** scientificName: *Hypenaamica*; **Location:** country: South Korea; stateProvince: GW; county: Wonju; locality: Geumdae-ri, Panbu-myeon; verbatimElevation: 543; verbatimCoordinates: 37°15'9.4"N 128°1'53.52"E; **Identification:** identifiedBy: Sei-Woong Choi.; dateIdentified: 2024; **Event:** samplingProtocol: Ultraviolet bucket trap; samplingEffort: 6 trapping hours; eventDate: 5/20/2024; **Record Level:** modified: 3/30/2025; language: en; collectionCode: Insects; basisOfRecord: PreservedSpecimen
- **Type status:** Other material. **Occurrence:** recordedBy: Choi, Sei-Woong; individualCount: 1; sex: male; lifeStage: adult; disposition: Mokpo National University; occurrenceID: 921E68C7-5B2D-532C-9D77-7E055B562336; **Taxon:** scientificName: *Hypenaamica*; **Location:** country: South Korea; stateProvince: GN; county: Yeosu; locality: Samsan-myeon; verbatimElevation: 26; verbatimCoordinates: 34°3'35"N 125°27'35"E; **Identification:** identifiedBy: Sei-Woong Choi; dateIdentified: 2024; **Event:** samplingProtocol: Ultraviolet bucket trap; samplingEffort: 6 trapping hours; eventDate: 5/27/2009; **Record Level:** modified: 3/30/2025; language: en; collectionCode: Insects; basisOfRecord: PreservedSpecimen
- **Type status:** Other material. **Occurrence:** recordedBy: Choi, Sei-Woong; individualCount: 1; sex: male; lifeStage: adult; disposition: Mokpo National University; occurrenceID: DEF4769C-F881-5E3E-A8A7-1B96FF5E24C6; **Taxon:** scientificName: *Hypenaamica*; **Location:** country: South Korea; stateProvince: GN; county: Sancheong; locality: Chahwang-myeon; verbatimElevation: 209; verbatimCoordinates: 35°26'44"N 127°26'53"E; **Identification:** identifiedBy: Sei-Woong Choi; dateIdentified: 2024; **Event:** samplingProtocol: Ultraviolet bucket trap; samplingEffort: 6 trapping hours; eventDate: 9/5/2008; **Record Level:** modified: 3/30/2025; language: en; collectionCode: Insects; basisOfRecord: PreservedSpecimen
- **Type status:** Other material. **Occurrence:** recordedBy: Choi, Sei-Woong; individualCount: 1; sex: male; lifeStage: adult; disposition: Mokpo National University; occurrenceID: 53DC4BD6-57C9-5305-AD06-7ABD87DADF7C; **Taxon:** scientificName: *Hypenaamica*; **Location:** country: South Korea; stateProvince: JN; county: Gurye; locality: Sandong-myeon; verbatimElevation: 931; verbatimCoordinates: 35°19'23"N 127°31'24.2"E; **Identification:** identifiedBy: Sei-Woong Choi; dateIdentified: 2024; **Event:** samplingProtocol: Ultraviolet bucket trap; samplingEffort: 6 trapping hours; eventDate: 6/6/2024; **Record Level:** modified: 3/30/2025; language: en; collectionCode: Insects; basisOfRecord: PreservedSpecimen
- **Type status:** Other material. **Occurrence:** recordedBy: Choi, Sei-Woong; individualCount: 1; sex: male; lifeStage: adult; disposition: Mokpo National University; occurrenceID: BB3F9FF9-6333-5177-8EF2-F926D8669B92; **Taxon:** scientificName: *Hypenaamica*; **Location:** country: South Korea; stateProvince: JN; county: Gurye; locality: Sandong-myeon; verbatimElevation: 923; verbatimCoordinates: 35°19'22"N 127°31'23"E; **Identification:** identifiedBy: Sei-Woong Choi; dateIdentified: 2024; **Event:** samplingProtocol: Ultraviolet bucket trap; samplingEffort: 6 trapping hours; eventDate: 7/28/2008; **Record Level:** modified: 3/30/2025; language: en; collectionCode: Insects; basisOfRecord: PreservedSpecimen
- **Type status:** Other material. **Occurrence:** recordedBy: Choi, Sei-Woong; individualCount: 1; sex: male; lifeStage: adult; disposition: Mokpo National University; occurrenceID: E8CB97EC-6B32-58B4-9BE3-4396EBD77E37; **Taxon:** scientificName: *Hypenaamica*; **Location:** country: South Korea; stateProvince: JN; county: Wando; locality: Cheongsan-myeon; verbatimElevation: 64; verbatimCoordinates: 34°12'4"N 126°46'3"E; **Identification:** identifiedBy: Sei-Woong Choi; dateIdentified: 2024; **Event:** samplingProtocol: Ultraviolet bucket trap; samplingEffort: 6 trapping hours; eventDate: 5/16/2009; **Record Level:** modified: 3/30/2025; language: en; collectionCode: Insects; basisOfRecord: PreservedSpecimen
- **Type status:** Other material. **Occurrence:** recordedBy: Choi, Sei-Woong; individualCount: 1; sex: male; lifeStage: adult; disposition: Mokpo National University; occurrenceID: CC53B1B3-CF26-584E-A391-BE1E890A7F10; **Taxon:** scientificName: *Hypenaamica*; **Location:** country: South Korea; stateProvince: JN; county: Muan; locality: Dorim-ri, Cheonggye-myeon; verbatimElevation: 52; verbatimCoordinates: 34°54'44"N 126°26'21"E; **Identification:** identifiedBy: Sei-Woong Choi.; dateIdentified: 2024; **Event:** samplingProtocol: Ultraviolet bucket trap; samplingEffort: 6 trapping hours; eventDate: 5/11/2009; **Record Level:** modified: 3/30/2025; language: en; collectionCode: Insects; basisOfRecord: PreservedSpecimen
- **Type status:** Other material. **Occurrence:** recordedBy: Choi, Sei-Woong; individualCount: 1; sex: male; lifeStage: adult; disposition: Mokpo National University; occurrenceID: C5331846-3A62-540C-9940-69731476832E; **Taxon:** scientificName: *Hypenaamica*; **Location:** country: South Korea; stateProvince: JN; county: Wando; locality: Cheongsan-myeon; verbatimElevation: 64; verbatimCoordinates: 34°12'4"N 126°46'3"E; **Identification:** identifiedBy: Sei-Woong Choi.; dateIdentified: 2024; **Event:** samplingProtocol: Ultraviolet bucket trap; samplingEffort: 6 trapping hours; eventDate: 5/16/2009; **Record Level:** modified: 3/30/2025; language: en; collectionCode: Insects; basisOfRecord: PreservedSpecimen
- **Type status:** Other material. **Occurrence:** recordedBy: Choi, Sei-Woong; individualCount: 1; sex: male; lifeStage: adult; disposition: Mokpo National University; occurrenceID: 93C18E28-0AD7-50E4-89A3-30436041CE47; **Taxon:** scientificName: *Hypenaamica*; **Location:** country: South Korea; stateProvince: JJ; county: Jeju; locality: Haean-dong; verbatimElevation: 954; verbatimCoordinates: 33°23'31.6"N 126°29'13"E; **Identification:** identifiedBy: Sei-Woong Choi; dateIdentified: 2024; **Event:** samplingProtocol: Ultraviolet bucket trap; samplingEffort: 6 trapping hours; eventDate: 8/6/2023; **Record Level:** modified: 3/30/2025; language: en; collectionCode: Insects; basisOfRecord: PreservedSpecimen
- **Type status:** Other material. **Occurrence:** recordedBy: Choi, Sei-Woong; individualCount: 1; sex: male; lifeStage: adult; disposition: Mokpo National University; occurrenceID: 5A165041-A6DD-5A92-A704-B11CE070D2D9; **Taxon:** scientificName: *Hypenaamica*; **Location:** country: South Korea; stateProvince: JJ; county: Seogwipo; locality: Mt. Halla; verbatimElevation: 615; verbatimCoordinates: 33°22'N 126°37’E; **Identification:** identifiedBy: Sei-Woong Choi; dateIdentified: 2024; **Event:** samplingProtocol: Ultraviolet bucket trap; samplingEffort: 6 trapping hours; eventDate: 10/28/2006; **Record Level:** modified: 3/30/2025; language: en; collectionCode: Insects; basisOfRecord: PreservedSpecimen
- **Type status:** Other material. **Occurrence:** recordedBy: Choi, Sei-Woong; individualCount: 1; sex: female; lifeStage: adult; disposition: Mokpo National University; occurrenceID: A330076F-BB24-5C76-B35B-A0A145F440F5; **Taxon:** scientificName: *Hypenaamica*; **Location:** country: South Korea; stateProvince: GN; county: Namhae; locality: Sangju-ri, Sangju-myeon; verbatimElevation: 559; verbatimCoordinates: 34°45'4.7"N 127°58'58.3"E; **Identification:** identifiedBy: Sei-Woong Choi; dateIdentified: 2024; **Event:** samplingProtocol: Ultraviolet bucket trap; samplingEffort: 6 trapping hours; eventDate: 9/18/2020; **Record Level:** modified: 3/30/2025; language: en; collectionCode: Insects; basisOfRecord: PreservedSpecimen
- **Type status:** Other material. **Occurrence:** recordedBy: Choi, Sei-Woong; individualCount: 1; sex: female; lifeStage: adult; disposition: Mokpo National University; occurrenceID: B272EF35-6E99-50EB-9D76-A8E3E460DDFA; **Taxon:** scientificName: *Hypenaamica*; **Location:** country: South Korea; stateProvince: GN; county: Hadong; locality: Hwagae-myeon; verbatimElevation: 686; verbatimCoordinates: 35°18'20.8"N 127°38'10.2"E; **Identification:** identifiedBy: Sei-Woong Choi; dateIdentified: 2024; **Event:** samplingProtocol: Ultraviolet bucket trap; samplingEffort: 6 trapping hours; eventDate: 5/8/2021; **Record Level:** modified: 3/30/2025; language: en; collectionCode: Insects; basisOfRecord: PreservedSpecimen
- **Type status:** Other material. **Occurrence:** recordedBy: Choi, Sei-Woong; individualCount: 1; sex: female; lifeStage: adult; disposition: Mokpo National University; occurrenceID: D22D06FF-3EF8-51CE-ADCE-9B6B99ACCD85; **Taxon:** scientificName: *Hypenaamica*; **Location:** country: South Korea; stateProvince: JB; county: Namwon; locality: Sannae-myeon; verbatimElevation: 515; verbatimCoordinates: 35°22'33.1"N 127°34'57.9"E; **Identification:** identifiedBy: Sei-Woong Choi; dateIdentified: 2024; **Event:** samplingProtocol: Ultraviolet bucket trap; samplingEffort: 6 trapping hours; eventDate: 5/12/2023; **Record Level:** modified: 3/30/2025; language: en; collectionCode: Insects; basisOfRecord: PreservedSpecimen
- **Type status:** Other material. **Occurrence:** recordedBy: Choi, Sei-Woong; individualCount: 1; sex: female; lifeStage: adult; disposition: Mokpo National University; occurrenceID: 673FA157-D9D7-5DF8-BEF0-92DD07D20911; **Taxon:** scientificName: *Hypenaamica*; **Location:** country: South Korea; stateProvince: JN; county: Gurye; locality: Toji-myeon; verbatimElevation: 1385; verbatimCoordinates: 35°18'16.2"N 127°33'43.6"E; **Identification:** identifiedBy: Sei-Woong Choi; dateIdentified: 2024; **Event:** samplingProtocol: Ultraviolet bucket trap; samplingEffort: 6 trapping hours; eventDate: 7/12/2021; **Record Level:** modified: 3/30/2025; language: en; collectionCode: Insects; basisOfRecord: PreservedSpecimen
- **Type status:** Other material. **Occurrence:** recordedBy: Choi, Sei-Woong; individualCount: 1; sex: female; lifeStage: adult; disposition: Mokpo National University; occurrenceID: C4883615-8BC4-5587-B106-E71D958400C4; **Taxon:** scientificName: *Hypenaamica*; **Location:** country: South Korea; stateProvince: JN; county: Gurye; locality: Sandong-myeon; verbatimElevation: 1504; verbatimCoordinates: 35°17'37.8"N 127°31'58.5"E; **Identification:** identifiedBy: Sei-Woong Choi; dateIdentified: 2024; **Event:** samplingProtocol: Ultraviolet bucket trap; samplingEffort: 6 trapping hours; eventDate: 7/12/2021; **Record Level:** modified: 3/30/2025; language: en; collectionCode: Insects; basisOfRecord: PreservedSpecimen
- **Type status:** Other material. **Occurrence:** recordedBy: Choi, Sei-Woong; individualCount: 1; sex: female; lifeStage: adult; disposition: Mokpo National University; occurrenceID: 5384DDCD-E538-57B7-B4A5-F9B09EBE4963; **Taxon:** scientificName: *Hypenaamica*; **Location:** country: South Korea; stateProvince: JN; county: Goheung; locality: Sinpyeong-ri, Geumsan-myeon; verbatimElevation: 522.7; verbatimCoordinates: 34°27'40.7"N 127°10'41"E; **Identification:** identifiedBy: Sei-Woong Choi; dateIdentified: 2024; **Event:** samplingProtocol: Ultraviolet bucket trap; samplingEffort: 6 trapping hours; eventDate: 8/12/2021; **Record Level:** modified: 3/30/2025; language: en; collectionCode: Insects; basisOfRecord: PreservedSpecimen
- **Type status:** Other material. **Occurrence:** recordedBy: Choi, Sei-Woong; individualCount: 1; sex: female; lifeStage: adult; disposition: Mokpo National University; occurrenceID: 2689381B-9FF0-5494-8F7A-5540673EDAB4; **Taxon:** scientificName: *Hypenaamica*; **Location:** country: South Korea; stateProvince: JN; county: Wando; locality: Cheongsan-myeon; verbatimElevation: 64; verbatimCoordinates: 34°12'4"N 126°46'3"E; **Identification:** identifiedBy: Sei-Woong Choi.; dateIdentified: 2024; **Event:** samplingProtocol: Ultraviolet bucket trap; samplingEffort: 6 trapping hours; eventDate: 5/16/2009; **Record Level:** modified: 3/30/2025; language: en; collectionCode: Insects; basisOfRecord: PreservedSpecimen
- **Type status:** Other material. **Occurrence:** recordedBy: Choi, Sei-Woong; individualCount: 1; sex: female; lifeStage: adult; disposition: Mokpo National University; occurrenceID: 5ABC0515-EAB0-53D9-892D-CF50069713F4; **Taxon:** scientificName: *Hypenaamica*; **Location:** country: South Korea; stateProvince: JN; county: Yeosu; locality: Yusong-ri, Nam-myeon; verbatimElevation: 118; verbatimCoordinates: 34°32'27"N 127°43'28"E; **Identification:** identifiedBy: Sei-Woong Choi; dateIdentified: 2024; **Event:** samplingProtocol: Ultraviolet bucket trap; samplingEffort: 6 trapping hours; eventDate: 6/21/2021; **Record Level:** modified: 3/30/2025; language: en; collectionCode: Insects; basisOfRecord: PreservedSpecimen
- **Type status:** Other material. **Occurrence:** recordedBy: Choi, Sei-Woong; individualCount: 1; sex: female; lifeStage: adult; disposition: Mokpo National University; occurrenceID: 6C1E733A-F269-5A5B-BD2F-BBE351AF036F; **Taxon:** scientificName: *Hypenaamica*; **Location:** country: South Korea; stateProvince: JN; county: Yeosu; locality: Samsan-myeon; verbatimElevation: 3; verbatimCoordinates: 34°3'36"N 127°23'19"E; **Identification:** identifiedBy: Sei-Woong Choi.; dateIdentified: 2024; **Event:** samplingProtocol: Ultraviolet bucket trap; samplingEffort: 6 trapping hours; eventDate: 5/27/2009; **Record Level:** modified: 3/30/2025; language: en; collectionCode: Insects; basisOfRecord: PreservedSpecimen
- **Type status:** Other material. **Occurrence:** recordedBy: Choi, Sei-Woong; individualCount: 1; sex: female; lifeStage: adult; disposition: Mokpo National University; occurrenceID: BED312A1-EB2B-5FAE-B77E-955F3606697B; **Taxon:** scientificName: *Hypenaamica*; **Location:** country: South Korea; stateProvince: JN; county: Yeosu; locality: Samsan-myeon; verbatimElevation: 26; verbatimCoordinates: 34°3'35"N 125°27'35"E; **Identification:** identifiedBy: Sei-Woong Choi; dateIdentified: 2024; **Event:** samplingProtocol: Ultraviolet bucket trap; samplingEffort: 6 trapping hours; eventDate: 5/27/2009; **Record Level:** modified: 3/30/2025; language: en; collectionCode: Insects; basisOfRecord: PreservedSpecimen
- **Type status:** Other material. **Occurrence:** recordedBy: Choi, Sei-Woong; individualCount: 1; sex: female; lifeStage: adult; disposition: Mokpo National University; occurrenceID: CD59B5FE-FF8C-5BB5-8E5E-7A700357E46B; **Taxon:** scientificName: *Hypenaamica*; **Location:** country: South Korea; stateProvince: JN; county: Yeusu; locality: Yusong-ri, Nam-myeon; verbatimCoordinates: 34.53575000'N 127.70860000’E; **Identification:** identifiedBy: Sei-Woong Choi; dateIdentified: 2024; **Event:** samplingProtocol: Ultraviolet bucket trap; samplingEffort: 6 trapping hours; eventDate: 5/2/2017; **Record Level:** modified: 3/30/2025; language: en; collectionCode: Insects; basisOfRecord: PreservedSpecimen
- **Type status:** Other material. **Occurrence:** recordedBy: Choi, Sei-Woong; individualCount: 1; sex: female; lifeStage: adult; disposition: Mokpo National University; occurrenceID: 11C9955C-888A-5EFE-8CF0-C5162D54068B; **Taxon:** scientificName: *Hypenaamica*; **Location:** country: South Korea; stateProvince: JN; county: Shinan; locality: Iheugam-ri, Imja-myeon; verbatimElevation: 91; verbatimCoordinates: 35°4'47"N 126°5'24"E; **Identification:** identifiedBy: Sei-Woong Choi; dateIdentified: 2024; **Event:** samplingProtocol: Ultraviolet bucket trap; samplingEffort: 6 trapping hours; eventDate: 9/14/2020; **Record Level:** modified: 3/30/2025; language: en; collectionCode: Insects; basisOfRecord: PreservedSpecimen
- **Type status:** Other material. **Occurrence:** recordedBy: Choi, Sei-Woong; individualCount: 1; sex: female; lifeStage: adult; disposition: Mokpo National University; occurrenceID: 0DD67063-E074-5EEA-9EE7-59B0DAFDD61F; **Taxon:** scientificName: *Hypenaamica*; **Location:** country: South Korea; stateProvince: JN; county: Jindo; locality: Jodo-myeon; verbatimElevation: 58; verbatimCoordinates: 34°14'17"N 125°54'27"E; **Identification:** identifiedBy: Sei-Woong Choi; dateIdentified: 2024; **Event:** samplingProtocol: Ultraviolet bucket trap; samplingEffort: 6 trapping hours; eventDate: 6/18/2009; **Record Level:** modified: 3/30/2025; language: en; collectionCode: Insects; basisOfRecord: PreservedSpecimen
- **Type status:** Other material. **Occurrence:** recordedBy: Choi, Sei-Woong; individualCount: 1; sex: female; lifeStage: adult; disposition: Mokpo National University; occurrenceID: C744BB7B-6DC1-5A92-90E3-748B3F393005; **Taxon:** scientificName: *Hypenaamica*; **Location:** country: South Korea; stateProvince: JN; county: Muan; locality: Cheonggye-ri, Cheonggye-myeon; verbatimElevation: 44; verbatimCoordinates: 34°54'27"N 126°25'14"E; **Identification:** identifiedBy: Sei-Woong Choi.; dateIdentified: 2024; **Event:** samplingProtocol: Ultraviolet bucket trap; samplingEffort: 6 trapping hours; eventDate: 5/3/2021; **Record Level:** modified: 3/30/2025; language: en; collectionCode: Insects; basisOfRecord: PreservedSpecimen
- **Type status:** Other material. **Occurrence:** recordedBy: Choi, Sei-Woong; individualCount: 5; sex: males; lifeStage: adult; disposition: Mokpo National University; occurrenceID: 5C83165D-E92B-5C7A-A562-057692A625BA; **Taxon:** scientificName: *Hypenaamica*; **Location:** country: South Korea; stateProvince: JN; county: Shinan; locality: Gageodo-ri, Heuksan-myeon; verbatimElevation: 110; verbatimCoordinates: 34°4'18.98"N 125°5'39.12"E; **Identification:** identifiedBy: Sei-Woong Choi.; dateIdentified: 2024; **Event:** samplingProtocol: Ultraviolet bucket trap; samplingEffort: 6 trapping hours; eventDate: 7/19/2022; **Record Level:** modified: 3/30/2025; language: en; collectionCode: Insects; basisOfRecord: PreservedSpecimen
- **Type status:** Other material. **Occurrence:** recordedBy: Choi, Sei-Woong; individualCount: 1; sex: female; lifeStage: adult; disposition: Mokpo National University; occurrenceID: 8BCC3FCE-EA2A-54DC-B789-64BE8AC1C56F; **Taxon:** scientificName: *Hypenaamica*; **Location:** country: South Korea; stateProvince: JN; county: Shinan; locality: Gageodo-ri, Heuksan-myeon; verbatimElevation: 110; verbatimCoordinates: 34°4'18.98"N 125°5'39.12"E; **Identification:** identifiedBy: Sei-Woong Choi.; dateIdentified: 2024; **Event:** samplingProtocol: Ultraviolet bucket trap; samplingEffort: 6 trapping hours; eventDate: 7/19/2022; **Record Level:** modified: 3/30/2025; language: en; collectionCode: Insects; basisOfRecord: PreservedSpecimen

##### Distribution

Korea, Japan, Taiwan.

##### Notes

Fig. 1e.

#### Hypena trigonalis

Guenée, 1854

01605E9C-168C-5BD3-97E3-A8D0BFE465BF

##### Materials

- **Type status:** Other material. **Occurrence:** recordedBy: Choi, Sei-Woong; individualCount: 1; sex: male; lifeStage: adult; disposition: Mokpo National University; occurrenceID: 4A367D37-059D-53A8-8E0E-BCC25054FF6D; **Taxon:** scientificName: *Hypenatrigonalis*; **Location:** country: South Korea; stateProvince: GB; county: Gunwi; locality: Nakjeon-ri, Goro-myeon; **Identification:** identifiedBy: Sung-Soo Kim; dateIdentified: 2024; **Event:** samplingProtocol: Ultraviolet bucket trap; samplingEffort: 6 trapping hours; eventDate: 5/10/2011; **Record Level:** modified: 3/30/2025; language: en; collectionCode: Insects; basisOfRecord: PreservedSpecimen
- **Type status:** Other material. **Occurrence:** recordedBy: Choi, Sei-Woong; individualCount: 1; sex: male; lifeStage: adult; disposition: Mokpo National University; occurrenceID: 87D10D4E-294E-5C5E-A07B-40AE4B088A35; **Taxon:** scientificName: *Hypenatrigonalis*; **Location:** country: South Korea; stateProvince: JN; county: Shinan; locality: Gageodo-ri, Heuksan-myeon; verbatimElevation: 110; verbatimCoordinates: 34°4'18.98"N 125°5'39.12"E; **Identification:** identifiedBy: Sei-Woong Choi; dateIdentified: 2024; **Event:** samplingProtocol: Ultraviolet bucket trap; samplingEffort: 6 trapping hours; eventDate: 7/19/2022; **Record Level:** modified: 3/30/2025; language: en; collectionCode: Insects; basisOfRecord: PreservedSpecimen
- **Type status:** Other material. **Occurrence:** recordedBy: Choi, Sei-Woong; individualCount: 1; sex: male; lifeStage: adult; disposition: Mokpo National University; occurrenceID: 1057DB80-F130-5E86-91DD-EEF938991E1B; **Taxon:** scientificName: *Hypenatrigonalis*; **Location:** country: South Korea; stateProvince: JJ; county: Jeju; locality: Nohyeong-dong; verbatimElevation: 673; verbatimCoordinates: 33°24'36.1"N 126°29'43.3"E; **Identification:** identifiedBy: Sei-Woong Choi; dateIdentified: 2024; **Event:** samplingProtocol: Ultraviolet bucket trap; samplingEffort: 6 trapping hours; eventDate: 9/6/2024; **Record Level:** modified: 3/30/2025; language: en; collectionCode: Insects; basisOfRecord: PreservedSpecimen
- **Type status:** Other material. **Occurrence:** recordedBy: Choi, Sei-Woong; individualCount: 2; sex: females; lifeStage: adult; disposition: Mokpo National University; occurrenceID: 1A613CA0-1DC7-585A-82CE-BAB931A29428; **Taxon:** scientificName: *Hypenatrigonalis*; **Location:** country: South Korea; stateProvince: JN; county: Jindo; locality: Jodo-myeon; **Identification:** identifiedBy: Sei-Woong Choi; dateIdentified: 2024; **Event:** samplingProtocol: Ultraviolet bucket trap; samplingEffort: 6 trapping hours; eventDate: 7/28/2008; **Record Level:** modified: 3/30/2025; language: en; collectionCode: Insects; basisOfRecord: PreservedSpecimen
- **Type status:** Other material. **Occurrence:** recordedBy: Choi, Sei-Woong; individualCount: 1; sex: female; lifeStage: adult; disposition: Mokpo National University; occurrenceID: C7C13B1B-3C9F-56EC-AEF9-69106BE2BAA4; **Taxon:** scientificName: *Hypenatrigonalis*; **Location:** country: South Korea; stateProvince: JN; county: Shinan; locality: Sugok-ri, Amtae-myeon; verbatimElevation: 170; verbatimCoordinates: 34°50'4"N 126°4'42"E; **Identification:** identifiedBy: Sei-Woong Choi; dateIdentified: 2024; **Event:** samplingProtocol: Ultraviolet bucket trap; samplingEffort: 6 trapping hours; eventDate: 8/24/2023; **Record Level:** modified: 3/30/2025; language: en; collectionCode: Insects; basisOfRecord: PreservedSpecimen
- **Type status:** Other material. **Occurrence:** recordedBy: Choi, Sei-Woong; individualCount: 1; sex: female; lifeStage: adult; disposition: Mokpo National University; occurrenceID: 9220C91B-F9E8-5B55-8CC4-827DFEF9E341; **Taxon:** scientificName: *Hypenatrigonalis*; **Location:** country: South Korea; stateProvince: JN; county: Muan; locality: Gangjeong-ri, Cheonggye-myeon; verbatimElevation: 68; verbatimCoordinates: 34°56'30"N 126°23'50"E; **Identification:** identifiedBy: Sei-Woong Choi; dateIdentified: 2024; **Event:** samplingProtocol: Ultraviolet bucket trap; samplingEffort: 6 trapping hours; eventDate: 7/3/2024; **Record Level:** modified: 3/30/2025; language: en; collectionCode: Insects; basisOfRecord: PreservedSpecimen
- **Type status:** Other material. **Occurrence:** recordedBy: Choi, Sei-Woong; individualCount: 1; sex: female; lifeStage: adult; disposition: Mokpo National University; occurrenceID: A374C57D-66BB-5729-BAC6-608F51C52AD6; **Taxon:** scientificName: *Hypenatrigonalis*; **Location:** country: South Korea; stateProvince: JN; county: Jindo; locality: Jodo-myeon; **Identification:** identifiedBy: Sei-Woong Choi; dateIdentified: 2024; **Event:** samplingProtocol: Ultraviolet bucket trap; samplingEffort: 6 trapping hours; eventDate: 7/18/2008; **Record Level:** modified: 3/30/2025; language: en; collectionCode: Insects; basisOfRecord: PreservedSpecimen
- **Type status:** Other material. **Occurrence:** recordedBy: Choi, Sei-Woong; individualCount: 1; sex: female; lifeStage: adult; disposition: Mokpo National University; occurrenceID: 8A986E3F-27A3-59D6-A93E-1A07B335E0A4; **Taxon:** scientificName: *Hypenatrigonalis*; **Location:** country: South Korea; stateProvince: JN; county: Jindo; locality: Yeomi-ri, Jodo-myeon; verbatimElevation: 2; verbatimCoordinates: 34°20'17.52"N 125°59'48.95"E; **Identification:** identifiedBy: Sei-Woong Choi; dateIdentified: 2024; **Event:** samplingProtocol: Ultraviolet bucket trap; samplingEffort: 6 trapping hours; eventDate: 5/14/2022; **Record Level:** modified: 3/30/2025; language: en; collectionCode: Insects; basisOfRecord: PreservedSpecimen
- **Type status:** Other material. **Occurrence:** recordedBy: Choi, Sei-Woong; individualCount: 1; sex: female; lifeStage: adult; disposition: Mokpo National University; occurrenceID: 5BAF7B90-2CC8-52BD-8CFD-D23EA915B664; **Taxon:** scientificName: *Hypenatrigonalis*; **Location:** country: South Korea; stateProvince: JJ; county: Namjeju-gun; locality: Namwon-eup; verbatimElevation: 636; verbatimCoordinates: 33°22'11"N 126°37'32"E; **Identification:** identifiedBy: Sei-Woong Choi; dateIdentified: 2024; **Event:** samplingProtocol: Ultraviolet bucket trap; samplingEffort: 6 trapping hours; eventDate: 9/8/2017; **Record Level:** modified: 3/30/2025; language: en; collectionCode: Insects; basisOfRecord: PreservedSpecimen
- **Type status:** Other material. **Occurrence:** recordedBy: Choi, Sei-Woong; individualCount: 1; sex: female; lifeStage: adult; disposition: Mokpo National University; occurrenceID: F2DEA12F-A16D-5BCE-85D5-61707E0168A7; **Taxon:** scientificName: *Hypenatrigonalis*; **Location:** country: South Korea; stateProvince: JJ; county: Seogwipo; locality: Hwasun-ro, Andeok-myeon; **Identification:** identifiedBy: Sung-Soo Kim; dateIdentified: 2024; **Event:** samplingProtocol: Ultraviolet bucket trap; samplingEffort: 6 trapping hours; eventDate: 8/28/2014; **Record Level:** modified: 3/30/2025; language: en; collectionCode: Insects; basisOfRecord: PreservedSpecimen
- **Type status:** Other material. **Occurrence:** recordedBy: Choi, Sei-Woong; individualCount: 1; sex: female; lifeStage: adult; disposition: Mokpo National University; occurrenceID: 4E98395F-9959-5A26-8C2F-3936185519F4; **Taxon:** scientificName: *Hypenatrigonalis*; **Location:** country: South Korea; stateProvince: JJ; county: Seogwipo; locality: Hawon-dong,; verbatimElevation: 963; verbatimCoordinates: 33°19'57.6"N 126°27'52.6"E; **Identification:** identifiedBy: Sei-Woong Choi; dateIdentified: 2024; **Event:** samplingProtocol: Ultraviolet bucket trap; samplingEffort: 6 trapping hours; eventDate: 8/6/2023; **Record Level:** modified: 3/30/2025; language: en; collectionCode: Insects; basisOfRecord: PreservedSpecimen
- **Type status:** Other material. **Occurrence:** recordedBy: Choi, Sei-Woong; individualCount: 1; sex: female; lifeStage: adult; disposition: Mokpo National University; occurrenceID: 6B8CCAD5-B0F7-5496-86E3-15A00721B50F; **Taxon:** scientificName: *Hypenatrigonalis*; **Location:** country: South Korea; stateProvince: JJ; county: Seogwipo; locality: Hawon-dong; verbatimElevation: 1109; verbatimCoordinates: 33°21'32.1"N 126°27'44.4"E; **Identification:** identifiedBy: Sei-Woong Choi; dateIdentified: 2024; **Event:** samplingProtocol: Ultraviolet bucket trap; samplingEffort: 6 trapping hours; eventDate: 8/6/2023; **Record Level:** modified: 3/30/2025; language: en; collectionCode: Insects; basisOfRecord: PreservedSpecimen
- **Type status:** Other material. **Occurrence:** recordedBy: Choi, Sei-Woong; individualCount: 1; sex: female; lifeStage: adult; disposition: Mokpo National University; occurrenceID: 3AFD9369-22CF-5387-8202-B332DB299EE3; **Taxon:** scientificName: *Hypenatrigonalis*; **Location:** country: South Korea; stateProvince: JJ; county: Seogwipo; locality: Harye-ri, Namwon-eup; verbatimElevation: 278; verbatimCoordinates: 33°18'57"N 126°37'9.9"E; **Identification:** identifiedBy: Sei-Woong Choi; dateIdentified: 2024; **Event:** samplingProtocol: Ultraviolet bucket trap; samplingEffort: 6 trapping hours; eventDate: 9/5/2023; **Record Level:** modified: 3/30/2025; language: en; collectionCode: Insects; basisOfRecord: PreservedSpecimen
- **Type status:** Other material. **Occurrence:** recordedBy: Choi, Sei-Woong; individualCount: 1; sex: female; lifeStage: adult; disposition: Mokpo National University; occurrenceID: 8BE35309-E434-50B0-873C-5E40CCD3BA94; **Taxon:** scientificName: *Hypenatrigonalis*; **Location:** country: South Korea; stateProvince: JJ; county: Seogwipo; locality: Harye-ri, Namwon-eup; verbatimElevation: 278; verbatimCoordinates: 33°18'57"N 126°37'9.9"E; **Identification:** identifiedBy: Sei-Woong Choi; dateIdentified: 2024; **Event:** samplingProtocol: Ultraviolet bucket trap; samplingEffort: 6 trapping hours; eventDate: 9/6/2024; **Record Level:** modified: 3/30/2025; language: en; collectionCode: Insects; basisOfRecord: PreservedSpecimen
- **Type status:** Other material. **Occurrence:** recordedBy: Choi, Sei-Woong; individualCount: 1; sex: female; lifeStage: adult; disposition: Mokpo National University; occurrenceID: 5F983800-32D3-545E-96AF-9A90909EA48E; **Taxon:** scientificName: *Hypenatrigonalis*; **Location:** country: South Korea; stateProvince: JJ; county: Jeju; locality: Haean-dong; verbatimElevation: 954; verbatimCoordinates: 33°23'31.6"N 126°29'13"E; **Identification:** identifiedBy: Sei-Woong Choi; dateIdentified: 2024; **Event:** samplingProtocol: Ultraviolet bucket trap; samplingEffort: 6 trapping hours; eventDate: 8/6/2023; **Record Level:** modified: 3/30/2025; language: en; collectionCode: Insects; basisOfRecord: PreservedSpecimen

##### Distribution

Korea, Taiwan, China (Hainan), India.

##### Notes

Fig. 1f.

#### Hypena proboscidalis

(Linnaeus, 1758)

2AA380FC-9976-5507-A6C0-8CA2998527A8

##### Materials

- **Type status:** Other material. **Occurrence:** recordedBy: Choi, Sei-Woong; individualCount: 1; sex: female; lifeStage: adult; disposition: Mokpo National University; occurrenceID: C3BEF8E3-EC1F-5BA4-8A27-99A9D6C0A0F3; **Taxon:** scientificName: *Hypenaproboscidalis*; **Location:** country: South Korea; stateProvince: GW; county: Yanggu; locality: Dumu-ri, Guktojeongjungang-myeon; **Identification:** identifiedBy: Sung-Soo Kim; dateIdentified: 2024; **Event:** samplingProtocol: Ultraviolet bucket trap; samplingEffort: 6 trapping hours; eventDate: 7/21/2023; **Record Level:** modified: 3/30/2025; language: en; collectionCode: Insects; basisOfRecord: PreservedSpecimen
- **Type status:** Other material. **Occurrence:** recordedBy: Choi, Sei-Woong; individualCount: 1; sex: male; lifeStage: adult; disposition: Mokpo National University; occurrenceID: 4934AA99-97CC-57CF-9479-B8E0430A800F; **Taxon:** scientificName: *Hypenaproboscidalis*; **Location:** country: South Korea; stateProvince: GW; county: Pyeongchang; locality: Mt. Jungwang, Daehwa-myeon; verbatimElevation: 1200; **Identification:** identifiedBy: Sung-Soo Kim; dateIdentified: 2024; **Event:** samplingProtocol: Ultraviolet bucket trap; samplingEffort: 6 trapping hours; eventDate: 6/11/2014; **Record Level:** modified: 3/30/2025; language: en; collectionCode: Insects; basisOfRecord: PreservedSpecimen
- **Type status:** Other material. **Occurrence:** recordedBy: Choi, Sei-Woong; individualCount: 1; sex: male; lifeStage: adult; disposition: Mokpo National University; occurrenceID: 99C55547-7392-59A9-A37F-DAB6F529FFB4; **Taxon:** scientificName: *Hypenaproboscidalis*; **Location:** country: South Korea; stateProvince: GW; county: Pyeongchang; locality: Mt. Jungwang, Daehwa-myeon; verbatimElevation: 1200; **Identification:** identifiedBy: Sung-Soo Kim; dateIdentified: 2024; **Event:** samplingProtocol: Ultraviolet bucket trap; samplingEffort: 6 trapping hours; eventDate: 6/10/2014; **Record Level:** modified: 3/30/2025; language: en; collectionCode: Insects; basisOfRecord: PreservedSpecimen
- **Type status:** Other material. **Occurrence:** recordedBy: Choi, Sei-Woong; individualCount: 1; sex: female; lifeStage: adult; disposition: Mokpo National University; occurrenceID: E5ABE1A2-A9D1-5BD5-AB18-7F88C8AA485D; **Taxon:** scientificName: *Hypenaproboscidalis*; **Location:** country: South Korea; stateProvince: CB; county: Goesan; locality: Hwayang-ri, Cheongcheon-myeon; **Identification:** identifiedBy: Sung-Soo Kim; dateIdentified: 2024; **Event:** samplingProtocol: Ultraviolet bucket trap; samplingEffort: 6 trapping hours; eventDate: 5/24/2014; **Record Level:** modified: 3/30/2025; language: en; collectionCode: Insects; basisOfRecord: PreservedSpecimen
- **Type status:** Other material. **Occurrence:** recordedBy: Choi, Sei-Woong; individualCount: 1; sex: female; lifeStage: adult; disposition: Mokpo National University; occurrenceID: 822B4B6A-113F-550D-8B10-A3BBB72E857C; **Taxon:** scientificName: *Hypenaproboscidalis*; **Location:** country: South Korea; stateProvince: JN; county: Gurye; locality: Mt.,Jiri; verbatimElevation: 1350; verbatimCoordinates: 35°17'N 127°33’E; **Identification:** identifiedBy: Sei-Woong Choi; dateIdentified: 2024; **Event:** samplingProtocol: Ultraviolet bucket trap; samplingEffort: 6 trapping hours; eventDate: 6/5/2005; **Record Level:** modified: 3/30/2025; language: en; collectionCode: Insects; basisOfRecord: PreservedSpecimen
- **Type status:** Other material. **Occurrence:** recordedBy: Choi, Sei-Woong; individualCount: 1; sex: female; lifeStage: adult; disposition: Mokpo National University; occurrenceID: 54C0EE5B-8923-571D-A8FB-13EF53CC98BE; **Taxon:** scientificName: *Hypenaproboscidalis*; **Location:** country: South Korea; stateProvince: JN; county: Sinan; locality: Sugok-ri, Amtae-myeon; verbatimElevation: 120; verbatimCoordinates: 34°50'19"N 126°4'55"E; **Identification:** identifiedBy: Sei-Woong Choi; dateIdentified: 2024; **Event:** samplingProtocol: Ultraviolet bucket trap; samplingEffort: 6 trapping hours; eventDate: 4/20/2023; **Record Level:** modified: 3/30/2025; language: en; collectionCode: Insects; basisOfRecord: PreservedSpecimen
- **Type status:** Other material. **Occurrence:** recordedBy: Choi, Sei-Woong; individualCount: 1; sex: female; lifeStage: adult; disposition: Mokpo National University; occurrenceID: 4FDC2838-3043-5D11-B94E-0DE9DD37E8C7; **Taxon:** scientificName: *Hypenaproboscidalis*; **Location:** country: South Korea; stateProvince: JJ; county: Jeju; locality: Mt. Halla, Aewol-eup; verbatimElevation: 1694; verbatimCoordinates: 33°21'44"N 126°31'10"E; **Identification:** identifiedBy: Sei-Woong Choi; dateIdentified: 2024; **Event:** samplingProtocol: Ultraviolet bucket trap; samplingEffort: 6 trapping hours; eventDate: 7/4/2019; **Record Level:** modified: 3/30/2025; language: en; collectionCode: Insects; basisOfRecord: PreservedSpecimen

##### Distribution

Korea, Japan, Russia, Taiwan, China (South), Nepal, Bangladesh, India, Europe.

##### Notes

**Figure 10.**
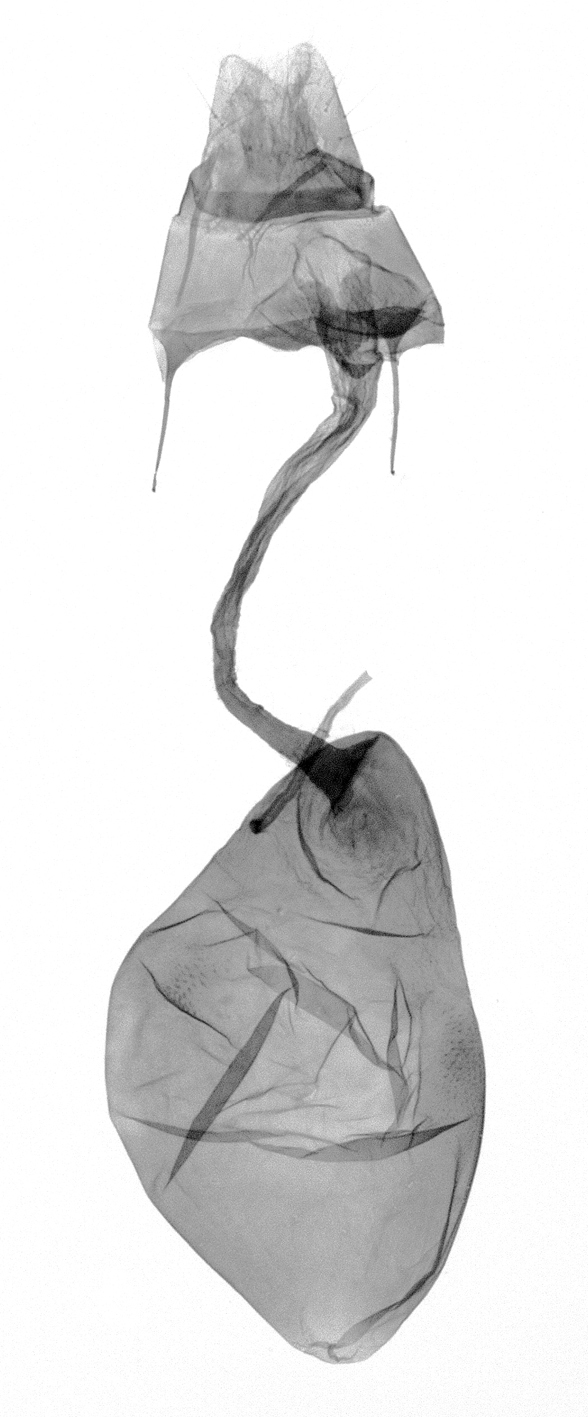
Female genitalia of *Hypenaproboscidalis* from Korea.

DNA barcoding. *Hypenaproboscidalis* (GenBank accession No. PV274479) showed the highest mean genetic distance of 10.25% from *H.pulverulenta*, while it exhibited the lowest mean genetic distance of 0.15% from *H.tamsi* (Figs 2a, 7c, d, 10).

#### Hypena tamsi

Filipjev, 1927

E4435642-7A24-55C4-B7FA-E4772AFC9AF4

##### Materials

- **Type status:** Other material. **Occurrence:** recordedBy: Choi, Sei-Woong; individualCount: 1; sex: male; lifeStage: adult; disposition: Mokpo National University; occurrenceID: 0C4858A0-A84B-5673-8B99-F9DD9691FB77; **Taxon:** scientificName: *Hypenatamsi*; **Location:** country: South Korea; stateProvince: JB; county: Sunchang; locality: Jiseon-ri, Bohheung-myon; **Identification:** identifiedBy: Sung-Soo Kim; dateIdentified: 2024; **Event:** samplingProtocol: Ultraviolet bucket trap; samplingEffort: 6 trapping hours; eventDate: 5/5/2013; **Record Level:** modified: 3/30/2025; language: en; collectionCode: Insects; basisOfRecord: PreservedSpecimen

##### Distribution

Korea, Russian Far East.

##### Notes

This is the first record for the Korean fauna. The DNA barcode of *Hypenatamsi* was first registered in this study (GenBank accession No. PV274484) and the p-distance with *H.pulverulenta* was 10.25%, while the lowest genetic distance of 0.15% was observed with *H.proboscidalis* (Figs 2b, 7e, f).

##### Diagnosis

Wingspan 35 mm. Antennae filiform with cilia; vertex anFd frons covered with long greyish-brown scales, distal tip ochreous; labial palpi long, covered with greyish-brown scales, second segment straight, more than three times longer than the third segment, third segment with ochreous distal end. Thorax greyish-brown; tegula consisting of long scales and hair-like scales. Forewing greyish-brown with numerous short dark brownish lines; antemedial line dark brown, undulating; postmedial line distinct, dark brownish, slightly curved at costal 2/3; subterminal line weakly undulating. Hindwing paler than forewing, greyish-brown. Abdomen greyish-brown. **Male genitalia**. Uncus long, weakly hooked, apex sharply pointed. Tegumen hood-shaped; tuba analis long; juxta broad, thick hexagon-shape on its side; saccus shallow, semi-rounded. Valva simple, stout, weakly sclerotised; costal margin almost flat, outer 1/4 weakly swollen; sacculus ventral margin medially weakly invaginated; basally a slender division of clavus with a minute process. Aedeagus rod-shaped, anteriorly with dense spicules; vesica large sac-shaped, cornuti a mass of large spines.

#### Hypena strigatus

(Fabricius, 1798)

472F5F45-7B8A-54FB-8368-4AB60A517C10

##### Materials

- **Type status:** Other material. **Occurrence:** recordedBy: Choi, Sei-Woong; individualCount: 1; sex: male; lifeStage: adult; disposition: Mokpo National University; occurrenceID: 9736F49B-DBB2-526C-A9BF-DD1D4CC7735A; **Taxon:** scientificName: *Hypenastrigatus*; **Location:** country: South Korea; stateProvince: JJ; county: Seogwipo; locality: Gangjeong-dong; **Identification:** identifiedBy: Sung-Soo Kim; dateIdentified: 2024; **Event:** samplingProtocol: Ultraviolet bucket trap; samplingEffort: 6 trapping hours; eventDate: 10/21/2022; **Record Level:** modified: 3/30/2025; language: en; collectionCode: Insects; basisOfRecord: PreservedSpecimen

##### Distribution

Korea, Japan, India.

##### Notes

Fig. 2c.

#### Hypena furva

Wileman, 1911

9DB41C80-A7B6-5A62-A579-245BD5AF4BE5

##### Distribution

Korea, Japan, Taiwan.

##### Notes

Leech (1889) was the first to document this species in Korea. Although Kononenko et al. (1998) did not examine this species, Kononenko and Han (2007) provided an illustration of the female genitalia of a specimen collected from Okcheon, Chungbuk.

#### Hypena conspersalis

Staudinger, 1888

22E9277A-6C2D-5807-A5E3-3070C913DC14

##### Materials

- **Type status:** Other material. **Occurrence:** recordedBy: Choi, Sei-Woong; individualCount: 1; sex: male; lifeStage: adult; disposition: Mokpo National University; occurrenceID: E8290304-4778-55AB-8C8D-4948DC6C3DE7; **Taxon:** scientificName: *Hypenaconspersalis*; **Location:** country: South Korea; stateProvince: GW; county: Pyeongchang; locality: Mt. Gyebang; verbatimElevation: 801; verbatimCoordinates: 37°41'59.69"N 128°28'47.6“E; **Identification:** identifiedBy: Sung-Soo Kim; dateIdentified: 2024; **Event:** samplingProtocol: Ultraviolet bucket trap; samplingEffort: 6 trapping hours; eventDate: 7/13/2010; **Record Level:** modified: 3/30/2025; language: en; collectionCode: Insects; basisOfRecord: PreservedSpecimen

##### Distribution

Korea, China (Jilin), Russian Far East.

##### Notes

Bryk (1949) was the first to document this species, with Park et al. (2001) later recording it in North Korea. In this study, we identified a male specimen from Gangwon Province, representing a new addition to the South Korean fauna (Fig. 2d).

#### Hypena sinuosa

Wileman, 1911

6D9098B7-789A-5AB5-84DB-7365DC0A154E

##### Materials

- **Type status:** Other material. **Occurrence:** recordedBy: Choi, Sei-Woong; individualCount: 1; sex: female; lifeStage: adult; disposition: Mokpo National University; occurrenceID: DE91B4DE-E030-5ABA-B01A-3A822C019FFB; **Taxon:** scientificName: *Hypenasinuosa*; **Location:** country: South Korea; stateProvince: GN; county: Geoje; locality: Gucheon-ri, Dongbu-myon; **Identification:** identifiedBy: Sung-Soo Kim; dateIdentified: 2024; **Event:** samplingProtocol: Ultraviolet bucket trap; samplingEffort: 6 trapping hours; eventDate: 8/25/2012; **Record Level:** modified: 3/30/2025; language: en; collectionCode: Insects; basisOfRecord: PreservedSpecimen
- **Type status:** Other material. **Occurrence:** recordedBy: Choi, Sei-Woong; individualCount: 1; sex: male; lifeStage: adult; disposition: Mokpo National University; occurrenceID: CBADEC20-3456-5A42-AE5B-36A612E04572; **Taxon:** scientificName: *Hypenasinuosa*; **Location:** country: South Korea; stateProvince: JJ; county: Mt. Halla; verbatimElevation: 525; verbatimCoordinates: 33°19′56.7″ 126°36′25.7″; **Identification:** identifiedBy: Sei-Woong Choi; dateIdentified: 2024; **Event:** samplingProtocol: Ultraviolet bucket trap; samplingEffort: 6 trapping hours; eventDate: 10/4/2024; **Record Level:** modified: 3/30/2025; language: en; collectionCode: Insects; basisOfRecord: PreservedSpecimen
- **Type status:** Other material. **Occurrence:** recordedBy: Choi, Sei-Woong; individualCount: 1; sex: male; lifeStage: adult; disposition: Mokpo National University; occurrenceID: 915DFE8C-97F2-5232-8D2E-94372048EB54; **Taxon:** scientificName: *Hypenasinuosa*; **Location:** country: South Korea; stateProvince: JJ; county: Mt. Halla; verbatimElevation: 525; verbatimCoordinates: 33°19′56.7″ 126°36′25.7″; **Identification:** identifiedBy: Sei-Woong Choi; dateIdentified: 2024; **Event:** samplingProtocol: Ultraviolet bucket trap; samplingEffort: 6 trapping hours; eventDate: 11/9/2024; **Record Level:** modified: 3/30/2025; language: en; collectionCode: Insects; basisOfRecord: PreservedSpecimen

##### Distribution

Korea, Japan, Taiwan.

##### Notes

Fig. 2e.

#### Hypena occata

Moore, 1882

DB1733DE-50AA-5BF0-B382-9413E73D8428

##### Materials

- **Type status:** Other material. **Occurrence:** recordedBy: Choi, Sei-Woong; individualCount: 1; sex: male; lifeStage: adult; disposition: Mokpo National University; occurrenceID: 266524F2-CE7F-5ED5-B70C-C9CA676AA39C; **Taxon:** scientificName: *Hypenaoccata*; **Location:** country: South Korea; stateProvince: GN; county: Tongyeong; locality: Sanyang-eup; **Identification:** identifiedBy: Sung-Soo Kim; dateIdentified: 2024; **Event:** samplingProtocol: Ultraviolet bucket trap; samplingEffort: 6 trapping hours; eventDate: 10/8/2014; **Record Level:** modified: 3/30/2025; language: en; collectionCode: Insects; basisOfRecord: PreservedSpecimen
- **Type status:** Other material. **Occurrence:** recordedBy: Choi, Sei-Woong; individualCount: 1; sex: male; lifeStage: adult; disposition: Mokpo National University; occurrenceID: B15117E1-7409-58E1-8476-EF2ADB330709; **Taxon:** scientificName: *Hypenaoccata*; **Location:** country: South Korea; stateProvince: GN; county: Tongyeong; **Identification:** identifiedBy: Sung-Soo Kim; dateIdentified: 2024; **Event:** samplingProtocol: Ultraviolet bucket trap; samplingEffort: 6 trapping hours; eventDate: 8/24/2019; **Record Level:** modified: 3/30/2025; language: en; collectionCode: Insects; basisOfRecord: PreservedSpecimen
- **Type status:** Other material. **Occurrence:** recordedBy: Choi, Sei-Woong; individualCount: 1; sex: female; lifeStage: adult; disposition: Mokpo National University; occurrenceID: A7943CBA-74B8-56BF-9FB3-DF15CCA00E35; **Taxon:** scientificName: *Hypenaoccata*; **Location:** country: South Korea; stateProvince: GN; county: Geoje; locality: Hakdong-ri, Dongbu-myeon; **Identification:** identifiedBy: Sung-Soo Kim; dateIdentified: 2024; **Event:** samplingProtocol: Ultraviolet bucket trap; samplingEffort: 6 trapping hours; eventDate: 4/9/2014; **Record Level:** modified: 3/30/2025; language: en; collectionCode: Insects; basisOfRecord: PreservedSpecimen
- **Type status:** Other material. **Occurrence:** recordedBy: Choi, Sei-Woong; individualCount: 1; sex: male; lifeStage: adult; disposition: Mokpo National University; occurrenceID: BD337962-93CC-55A5-9643-14F5D28EA9D7; **Taxon:** scientificName: *Hypenaoccata*; **Location:** country: South Korea; stateProvince: JJ; county: Jeju; locality: Gujwa-eup; **Identification:** identifiedBy: Sung-Soo Kim; dateIdentified: 2024; **Event:** samplingProtocol: Ultraviolet bucket trap; samplingEffort: 6 trapping hours; eventDate: 10/3/2000; **Record Level:** modified: 3/30/2025; language: en; collectionCode: Insects; basisOfRecord: PreservedSpecimen
- **Type status:** Other material. **Occurrence:** recordedBy: Choi, Sei-Woong; individualCount: 1; sex: female; lifeStage: adult; disposition: Mokpo National University; occurrenceID: BD2B6CA7-DAB3-5B04-9C4D-013E56FD819D; **Taxon:** scientificName: *Hypenaoccata*; **Location:** country: South Korea; stateProvince: JJ; county: Jeju; locality: Gujwa-eup; **Identification:** identifiedBy: Sung-Soo Kim; dateIdentified: 2024; **Event:** samplingProtocol: Ultraviolet bucket trap; samplingEffort: 6 trapping hours; eventDate: 10/3/2000; **Record Level:** modified: 3/30/2025; language: en; collectionCode: Insects; basisOfRecord: PreservedSpecimen
- **Type status:** Other material. **Occurrence:** recordedBy: Choi, Sei-Woong; individualCount: 1; sex: female; lifeStage: adult; disposition: Mokpo National University; occurrenceID: 76D67263-0CB4-57EA-85E1-340EC3207B23; **Taxon:** scientificName: *Hypenaoccata*; **Location:** country: South Korea; stateProvince: JJ; county: Jeju; locality: Jeoji-ri, Hangyeong-myeon; **Identification:** identifiedBy: Sung-Soo Kim; dateIdentified: 2024; **Event:** samplingProtocol: Ultraviolet bucket trap; samplingEffort: 6 trapping hours; eventDate: 3/20/2020; **Record Level:** modified: 3/30/2025; language: en; collectionCode: Insects; basisOfRecord: PreservedSpecimen
- **Type status:** Other material. **Occurrence:** recordedBy: Choi, Sei-Woong; individualCount: 1; sex: female; lifeStage: adult; disposition: Mokpo National University; occurrenceID: E2C0F95C-EFC3-5F7C-A75A-19214A01E78C; **Taxon:** scientificName: *Hypenaoccata*; **Location:** country: South Korea; stateProvince: JJ; county: Seogwipo; locality: Sagye-ri, Andeok-myeon; **Identification:** identifiedBy: Sung-Soo Kim; dateIdentified: 2024; **Event:** samplingProtocol: Ultraviolet bucket trap; samplingEffort: 6 trapping hours; eventDate: 6/23/2017; **Record Level:** modified: 3/30/2025; language: en; collectionCode: Insects; basisOfRecord: PreservedSpecimen
- **Type status:** Other material. **Occurrence:** recordedBy: Choi, Sei-Woong; individualCount: 1; sex: female; lifeStage: adult; disposition: Mokpo National University; occurrenceID: 14DDB563-92CF-52E6-BD14-4504980B3033; **Taxon:** scientificName: *Hypenaoccata*; **Location:** country: South Korea; stateProvince: JJ; county: Seogwipo; locality: Jungmun-dong; **Identification:** identifiedBy: Sung-Soo Kim; dateIdentified: 2024; **Event:** samplingProtocol: Ultraviolet bucket trap; samplingEffort: 6 trapping hours; eventDate: 3/28/2018; **Record Level:** modified: 3/30/2025; language: en; collectionCode: Insects; basisOfRecord: PreservedSpecimen

##### Distribution

Korea, Japan, Taiwan.

##### Notes

Leech (1889) recorded this species from Wonsan (Gensan), North Korea. Based on the specimens examined in this study, the first record of the species is ambiguous (Figs 2f, 3a).

#### Hypena indicatalis

Walker, 1859

0A9FA79D-5EB0-57D6-A3EE-D8E12DF32500

##### Materials

- **Type status:** Other material. **Occurrence:** recordedBy: Choi, Sei-Woong; individualCount: 1; sex: female; lifeStage: adult; disposition: Mokpo National University; occurrenceID: AF4B9C97-2FD8-551C-AF55-64A8B4FD5CC3; **Taxon:** scientificName: *Hypenaindicatalis*; **Location:** country: South Korea; stateProvince: JN; county: Jindo; locality: Sinjeon-ri, Jodo-myeon; **Identification:** identifiedBy: Sung-Soo Kim; dateIdentified: 2024; **Event:** samplingProtocol: Ultraviolet bucket trap; samplingEffort: 6 trapping hours; eventDate: 10/10/2018; **Record Level:** modified: 3/30/2025; language: en; collectionCode: Insects; basisOfRecord: PreservedSpecimen

##### Distribution

Korea, Japan, Taiwan, India.

##### Notes

Leech (1889) recorded this species from Wonsan (Gensan), North Korea. Based on the specimens examined in this study, the first record of the species is ambiguous (Fig. 3b).

#### Hypena subcyanea

Butler, 1880

A735EDCC-C099-5EFB-AB8F-E57DD0FE3ED7

##### Materials

- **Type status:** Other material. **Occurrence:** recordedBy: Choi, Sei-Woong; individualCount: 1; sex: male; lifeStage: adult; disposition: Mokpo National University; occurrenceID: 1F662305-8128-5E13-BC9D-B3A7572E8E35; **Taxon:** scientificName: *Hypenasubcyanea*; **Location:** country: South Korea; stateProvince: GN; county: Sacheon; locality: Seojeong-ri, Gonyang-myeon; **Identification:** identifiedBy: Sung-Soo Kim; dateIdentified: 2024; **Event:** samplingProtocol: Ultraviolet bucket trap; samplingEffort: 6 trapping hours; eventDate: 10/3/2023; **Record Level:** modified: 3/30/2025; language: en; collectionCode: Insects; basisOfRecord: PreservedSpecimen
- **Type status:** Other material. **Occurrence:** recordedBy: Choi, Sei-Woong; individualCount: 1; sex: male; lifeStage: adult; disposition: Mokpo National University; occurrenceID: 00F32366-3725-5BF1-927A-ABD1ABEB7D63; **Taxon:** scientificName: *Hypenasubcyanea*; **Location:** country: South Korea; stateProvince: GN; county: Geoje; locality: Gucheon-ri, Dongbu-myeon; **Identification:** identifiedBy: Sung-Soo Kim; dateIdentified: 2024; **Event:** samplingProtocol: Ultraviolet bucket trap; samplingEffort: 6 trapping hours; eventDate: 9/25/2012; **Record Level:** modified: 3/30/2025; language: en; collectionCode: Insects; basisOfRecord: PreservedSpecimen
- **Type status:** Other material. **Occurrence:** recordedBy: Choi, Sei-Woong; individualCount: 1; sex: male; lifeStage: adult; disposition: Mokpo National University; occurrenceID: 868366BC-BC93-527C-ACE7-53A4094C6E29; **Taxon:** scientificName: *Hypenasubcyanea*; **Location:** country: South Korea; stateProvince: JN; county: Yeosu; locality: Yulchon-myeon; **Identification:** identifiedBy: Sung-Soo Kim; dateIdentified: 2024; **Event:** samplingProtocol: Ultraviolet bucket trap; samplingEffort: 6 trapping hours; eventDate: 9/28/2022; **Record Level:** modified: 3/30/2025; language: en; collectionCode: Insects; basisOfRecord: PreservedSpecimen

##### Distribution

Korea, Japan, Taiwan.

##### Notes

Leech (1889) and Leech (1900) recorded this species from Wonsan (Gensan), North Korea. Based on the specimens examined in this study, the first record of the species is ambiguous (Fig. 3c).

#### Hypena obacerralis

Walker, 1859

0D6A873E-30DC-568C-B003-937015A1095B

urn:lsid:zoobank.org:act:A4417862-62F2-433B-BCC9-E677576FF1C9

Hypena longipalpis Guenée, 1862 - Guenée (1862) | *Hypenaferriscitalis* Walker, 1866 -Walker (1866) | *Hypenacomes* Butler, 1882 - Butler (1882) | *Hypenasordida* Rothschild, 1921 - Rothschild (1921).

##### Distribution

Korea, Middle East and South Asia (India, Sri Lanka, Malaysia), Australia, Africa.

##### Notes

This is the first record for the Korean fauna. The DNA barcode of *Hypenaobacerralis* was first registered in this study (GenBank accession No. PV274486) and the p-distance with *H.zilla* was 9.32%, while the lowest genetic distance of 7.39% was observed with *H.obesalis* (Figs 3d, 9).

##### Diagnosis

Wingspan 24 mm. Antennae filiform; vertex and frons narrow, greyish-brown, covered with long erected scales; labial palpi dark brown, covered with erected scales on dorsal and ventral surfaces, second segment two times longer than the third segment. Thorax greyish-brown; tegula consisting of long scales and hair-like scales. Forewing ground colour greyish-brown; antemedial and postmedial lines reddish-brown, parallel, strongly oblique. Hindwing greyish-brown, without medial lines; veins distinct with dark greyish-brown. **Male genitalia.** Uncus long, tapering, apex strongly sclerotised, pointed. Tegumen hood-shaped; tuba analis long; saccus short, semi-rounded. Valva simple, weakly sclerotised, medially expanded; costa basally strongly sclerotised, dorsal margin medially strongly swollen; sacculus dorsally with a strongly swollen medial process and a nipple-shaped distal process, ventral margin weakly swollen; basally a relatively thin division of clavus. Aedeagus long, rod-shaped, anteriorly with dense spicules; vesica large, tubular, cornuti a patch of spines.

#### Hypena tristalis

Lederer, 1853

3DD11693-8FB4-5234-9252-C4A1B9FA11AD

##### Materials

- **Type status:** Other material. **Occurrence:** recordedBy: Choi, Sei-Woong; individualCount: 1; sex: female; lifeStage: adult; disposition: Mokpo National University; occurrenceID: 27B1E79B-B457-5EC3-81C3-72161220504C; **Taxon:** scientificName: *Hypenatristalis*; **Location:** country: South Korea; stateProvince: GW; county: Yanggu; locality: Dumu-ri, Guktojeongjungang-myeon; **Identification:** identifiedBy: Sung-Soo Kim; dateIdentified: 2024; **Event:** samplingProtocol: Ultraviolet bucket trap; samplingEffort: 6 trapping hours; eventDate: 7/21/2023; **Record Level:** modified: 3/30/2025; language: en; collectionCode: Insects; basisOfRecord: PreservedSpecimen
- **Type status:** Other material. **Occurrence:** recordedBy: Choi, Sei-Woong; individualCount: 1; sex: male; lifeStage: adult; disposition: Mokpo National University; occurrenceID: 3BDECC4D-6C05-502B-B927-43BDF0E9BA5D; **Taxon:** scientificName: *Hypenatristalis*; **Location:** country: South Korea; stateProvince: GW; county: Inje; locality: Mt. Seorak, Buk-myeon; verbatimElevation: 1700; **Identification:** identifiedBy: Sung-Soo Kim; dateIdentified: 2024; **Event:** samplingProtocol: Ultraviolet bucket trap; samplingEffort: 6 trapping hours; eventDate: 8/1/2013; **Record Level:** modified: 3/30/2025; language: en; collectionCode: Insects; basisOfRecord: PreservedSpecimen
- **Type status:** Other material. **Occurrence:** recordedBy: Choi, Sei-Woong; individualCount: 1; sex: female; lifeStage: adult; disposition: Mokpo National University; occurrenceID: B9FF6AB4-A800-5FA3-9898-40E53E2DE10A; **Taxon:** scientificName: *Hypenatristalis*; **Location:** country: South Korea; stateProvince: GW; county: Inje; locality: Bangdong-ri, Girin-myeon; **Identification:** identifiedBy: Sung-Soo Kim; dateIdentified: 2024; **Event:** samplingProtocol: Ultraviolet bucket trap; samplingEffort: 6 trapping hours; eventDate: 5/12/2013; **Record Level:** modified: 3/30/2025; language: en; collectionCode: Insects; basisOfRecord: PreservedSpecimen
- **Type status:** Other material. **Occurrence:** recordedBy: Choi, Sei-Woong; individualCount: 2; sex: females; lifeStage: adult; disposition: Mokpo National University; occurrenceID: 38764FCF-B3C1-5768-AE41-09D25E9CE81B; **Taxon:** scientificName: *Hypenatristalis*; **Location:** country: South Korea; stateProvince: GW; county: Pyeongchang; locality: Mt. Jungwang, Daehwa-myeon; verbatimElevation: 1200; **Identification:** identifiedBy: Sung-Soo Kim; dateIdentified: 2024; **Event:** samplingProtocol: Ultraviolet bucket trap; samplingEffort: 6 trapping hours; eventDate: 9/17/2013; **Record Level:** modified: 3/30/2025; language: en; collectionCode: Insects; basisOfRecord: PreservedSpecimen
- **Type status:** Other material. **Occurrence:** recordedBy: Choi, Sei-Woong; individualCount: 1; sex: female; lifeStage: adult; disposition: Mokpo National University; occurrenceID: 2E7595B8-A428-58D2-9966-2EB9957F3598; **Taxon:** scientificName: *Hypenatristalis*; **Location:** country: South Korea; stateProvince: JN; county: Gurye; locality: Mt. Jiri, Toji-myeon; verbatimElevation: 1318; verbatimCoordinates: 35°18'12"N 127°33'34“E; **Identification:** identifiedBy: Sei-Woong Choi; dateIdentified: 2024; **Event:** samplingProtocol: Ultraviolet bucket trap; samplingEffort: 6 trapping hours; eventDate: 9/20/2009; **Record Level:** modified: 3/30/2025; language: en; collectionCode: Insects; basisOfRecord: PreservedSpecimen
- **Type status:** Other material. **Occurrence:** recordedBy: Choi, Sei-Woong; individualCount: 1; sex: female; lifeStage: adult; disposition: Mokpo National University; occurrenceID: 71D46044-009B-58F5-8EF9-7B992A2057BE; **Taxon:** scientificName: *Hypenatristalis*; **Location:** country: South Korea; stateProvince: JN; county: Gurye; locality: Mt. Jiri,Sandong-myeon; verbatimElevation: 1504; verbatimCoordinates: 35°17'37.8"N 127°31'58.5“E; **Identification:** identifiedBy: Sei-Woong Choi; dateIdentified: 2024; **Event:** samplingProtocol: Ultraviolet bucket trap; samplingEffort: 6 trapping hours; eventDate: 5/16/2020; **Record Level:** modified: 3/30/2025; language: en; collectionCode: Insects; basisOfRecord: PreservedSpecimen
- **Type status:** Other material. **Occurrence:** recordedBy: Choi, Sei-Woong; individualCount: 1; sex: female; lifeStage: adult; disposition: Mokpo National University; occurrenceID: BB437A0C-FEFB-51DD-8FA8-436413F8D99F; **Taxon:** scientificName: *Hypenatristalis*; **Location:** country: South Korea; stateProvince: JN; county: Gurye; locality: Mt. Jiri, Sandong-myeon; verbatimElevation: 1074; verbatimCoordinates: 35°18'21"N 127°30'45“E; **Identification:** identifiedBy: Sei-Woong Choi; dateIdentified: 2024; **Event:** samplingProtocol: Ultraviolet bucket trap; samplingEffort: 6 trapping hours; eventDate: 9/14/2012; **Record Level:** modified: 3/30/2025; language: en; collectionCode: Insects; basisOfRecord: PreservedSpecimen

##### Distribution

Korea, Japan, China, Russian Far East.

##### Notes

*Hypenatristalis* (GenBank accession No. JN273650, JN273651) showed the highest mean genetic distance of 8.71% from *H.trigonalis*, while it exhibited the lowest mean genetic distance of 4.13% from *H.obesalis* (Fig. 3e).

#### Hypena narratalis

Walker, 1859

46E1783D-15C4-5D37-AABF-2E4F2C5CE155

##### Materials

- **Type status:** Other material. **Occurrence:** recordedBy: Choi, Sei-Woong; individualCount: 1; sex: male; lifeStage: adult; disposition: Mokpo National University; occurrenceID: A3109A78-BC30-5F8E-B9B5-63E15ED8DCD1; **Taxon:** scientificName: *Hypenanarratalis*; **Location:** country: South Korea; stateProvince: GW; county: Pyungchang; locality: Mt. Jungangsan,Daewha-myeon; **Identification:** identifiedBy: Sung-Soo Kim; dateIdentified: 2024; **Event:** samplingProtocol: Ultraviolet bucket trap; samplingEffort: 6 trapping hours; eventDate: 6/13/2012; **Record Level:** modified: 3/30/2025; language: en; collectionCode: Insects; basisOfRecord: PreservedSpecimen
- **Type status:** Other material. **Occurrence:** recordedBy: Choi, Sei-Woong; individualCount: 1; sex: male; lifeStage: adult; disposition: Mokpo National University; occurrenceID: ED5F831C-E275-58AA-9C75-08EEFDD2BB3A; **Taxon:** scientificName: *Hypenanarratalis*; **Location:** country: South Korea; stateProvince: GW; county: Pyungchang; locality: Mt. Jungangsan,Daewha-myeon; verbatimElevation: 1200; **Identification:** identifiedBy: Sung-Soo Kim; dateIdentified: 2024; **Event:** samplingProtocol: Ultraviolet bucket trap; samplingEffort: 6 trapping hours; eventDate: 5/14/2015; **Record Level:** modified: 3/30/2025; language: en; collectionCode: Insects; basisOfRecord: PreservedSpecimen
- **Type status:** Other material. **Occurrence:** recordedBy: Choi, Sei-Woong; individualCount: 1; sex: female; lifeStage: adult; disposition: Mokpo National University; occurrenceID: CC27B8DD-C138-5CD2-B1FC-2E3DEE539D1F; **Taxon:** scientificName: *Hypenanarratalis*; **Location:** country: South Korea; stateProvince: GW; county: Inje; locality: Bangtong-ri, Girin-myon; **Identification:** identifiedBy: Sung-Soo Kim; dateIdentified: 2024; **Event:** samplingProtocol: Ultraviolet bucket trap; samplingEffort: 6 trapping hours; eventDate: 5/8/2011; **Record Level:** modified: 3/30/2025; language: en; collectionCode: Insects; basisOfRecord: PreservedSpecimen
- **Type status:** Other material. **Occurrence:** recordedBy: Choi, Sei-Woong; individualCount: 1; sex: male; lifeStage: adult; disposition: Mokpo National University; occurrenceID: D42BF003-FFFC-5458-8FD6-B16996D8FF46; **Taxon:** scientificName: *Hypenanarratalis*; **Location:** country: South Korea; stateProvince: CB; county: Danyang; locality: Mt. Soback; verbatimElevation: 530; verbatimCoordinates: 36°59'N 128°28'E; **Identification:** identifiedBy: Sei-Woong Choi; dateIdentified: 2024; **Event:** samplingProtocol: Ultraviolet bucket trap; samplingEffort: 6 trapping hours; eventDate: 7/21/2005; **Record Level:** modified: 3/30/2025; language: en; collectionCode: Insects; basisOfRecord: PreservedSpecimen

##### Distribution

Korea, Japan, Taiwan, India (North).

##### Notes

*Hypenanarratalis* (GenBank accession No. PV274487) showed the highest mean genetic distance of 8.71% from *H.trigonalis*, while it exhibited the lowest mean genetic distance of 4.29% from *H.obesalis* (Figs 3f, 4a).

#### Hypena pulverulenta

Wileman, 1911

37740721-7CB1-5C54-8AD2-2DE3D8CF0DC6

##### Materials

- **Type status:** Other material. **Occurrence:** recordedBy: Choi, Sei-Woong; individualCount: 1; sex: male; lifeStage: adult; disposition: Mokpo National University; occurrenceID: C81FAE0B-B02A-5846-A835-07AD4E5A9A15; **Taxon:** scientificName: *Hypenapulverulenta*; **Location:** country: South Korea; stateProvince: Sejong; county: Sejong; locality: Geumnam-myeon; verbatimCoordinates: 36°27'33"N 127°17'53E; **Identification:** identifiedBy: Sung-Soo Kim; dateIdentified: 2024; **Event:** samplingProtocol: Ultraviolet bucket trap; samplingEffort: 6 trapping hours; eventDate: 9/5/2022; **Record Level:** modified: 3/30/2025; language: en; collectionCode: Insects; basisOfRecord: PreservedSpecimen

##### Distribution

Korea, Japan.

##### Notes

This is the first record for the Korean fauna. The DNA barcode of *Hypenapulverulenta* was first recorded in this study (GenBank accession No. PV274491). The p-distance with *H.proboscidalis* and *H.tamsi* was 10.25%, while the lowest genetic distance (6.63%) was recorded for *H.obesalis* (Figs 4b, 8a, b).

##### Diagnosis

Wingspan 24 mm. Antenna filiform covered with yellowish-brown scales; vertex and frons covered with yellowish-brown erected scales; labial palpi long, second segment yellowish-brown, six times longer than the third segment, third segment covered with blackish scales. Thorax yellowish-brown; tegula consisting of long scales and hair-like scales. Forewing ground colour dark brownish, dorsally ochreous with yellowish-brown; central fascia large triangular; discal dot small black; subterminal line costally blackish; apex distinct with a whitish oblique line. Hindwing pale yellowish-brown. Abdomen yellowish-brown. **Male genitalia.** Uncus long, rod-shaped, apex strongly sclerotised, sharply hooked, pointed. Tegumen hood-shaped; tuba analis long; juxta broad, thick diamond-shape on its side; saccus long, semi-rounded. Valva simple, weakly sclerotised, medially expanded; costal margin medially swollen; sacculus ventral margin medially weakly invaginated; basally a relatively thick and long division of clavus with a minute process. Aedeagus rod-shaped, anteriorly simple; vesica tubular, cornuti a row of spines.

#### Hypena abducalis

(Walker, 1859)

616F2733-DB5F-59E2-8DEC-25B5673E0577

##### Materials

- **Type status:** Other material. **Occurrence:** recordedBy: Choi, Sei-Woong; individualCount: 1; sex: female; lifeStage: adult; disposition: Mokpo National University; occurrenceID: CBE95168-27C6-5B1D-ABFA-4FD90E56F7B2; **Taxon:** scientificName: *Hypenaabducalis*; **Location:** country: South Korea; stateProvince: Incheon; county: Incheon; locality: Muui-dong, Jung-gu; **Identification:** identifiedBy: Sung-Soo Kim; dateIdentified: 2024; **Event:** samplingProtocol: Ultraviolet bucket trap; samplingEffort: 6 trapping hours; eventDate: 8/31/2022; **Record Level:** modified: 3/30/2025; language: en; collectionCode: Insects; basisOfRecord: PreservedSpecimen
- **Type status:** Other material. **Occurrence:** recordedBy: Choi, Sei-Woong; individualCount: 1; sex: female; lifeStage: adult; disposition: Mokpo National University; occurrenceID: E42B65BC-57C1-5AF5-B330-05B76B427352; **Taxon:** scientificName: *Hypenaabducalis*; **Location:** country: South Korea; stateProvince: GB; county: Gunwi; locality: Nakjeon-ri, Goro-myeon; **Identification:** identifiedBy: Sung-Soo Kim; dateIdentified: 2024; **Event:** samplingProtocol: Ultraviolet bucket trap; samplingEffort: 6 trapping hours; eventDate: 5/10/2011; **Record Level:** modified: 3/30/2025; language: en; collectionCode: Insects; basisOfRecord: PreservedSpecimen
- **Type status:** Other material. **Occurrence:** recordedBy: Choi, Sei-Woong; individualCount: 1; sex: male; lifeStage: adult; disposition: Mokpo National University; occurrenceID: 0B991DCC-83C9-56DC-936F-8793284B2451; **Taxon:** scientificName: *Hypenaabducalis*; **Location:** country: South Korea; stateProvince: JN; county: Muan; locality: Gangjeong-ri, Cheonggye-myeon; verbatimElevation: 68; verbatimCoordinates: 34°56'30"N 126°23'50“E; **Identification:** identifiedBy: Sei-Woong Choi; dateIdentified: 2024; **Event:** samplingProtocol: Ultraviolet bucket trap; samplingEffort: 6 trapping hours; eventDate: 6/3/2019; **Record Level:** modified: 3/30/2025; language: en; collectionCode: Insects; basisOfRecord: PreservedSpecimen
- **Type status:** Other material. **Occurrence:** recordedBy: Choi, Sei-Woong; individualCount: 1; sex: male; lifeStage: adult; disposition: Mokpo National University; occurrenceID: 84BC9469-A3DA-56DD-B4E0-48AA7A910AD1; **Taxon:** scientificName: *Hypenaabducalis*; **Location:** country: South Korea; stateProvince: JJ; county: Jeju; locality: near Imperial Hotel; **Identification:** identifiedBy: Sung-Soo Kim; dateIdentified: 2024; **Event:** samplingProtocol: Ultraviolet bucket trap; samplingEffort: 6 trapping hours; eventDate: 5/24/2002; **Record Level:** modified: 3/30/2025; language: en; collectionCode: Insects; basisOfRecord: PreservedSpecimen

##### Distribution

Korea, Japan, China (South), India.

##### Notes

Fig. 4c, d.

#### Hypena kengkalis

Bremer, 1864

F682D5FE-ABF6-5905-ACCE-BC356FCD8C0F

##### Materials

- **Type status:** Other material. **Occurrence:** recordedBy: Choi, Sei-Woong; individualCount: 1; sex: male; lifeStage: adult; disposition: Mokpo National University; occurrenceID: 16E6DF78-4786-5F89-808B-C93FAAC7762B; **Taxon:** scientificName: *Hypenakengkalis*; **Location:** country: South Korea; stateProvince: GW; county: Inje; locality: Wondae-ri, Inje-eup; **Identification:** identifiedBy: Sung-Soo Kim; dateIdentified: 2024; **Event:** samplingProtocol: Ultraviolet bucket trap; samplingEffort: 6 trapping hours; eventDate: 7/20/2023; **Record Level:** modified: 3/30/2025; language: en; collectionCode: Insects; basisOfRecord: PreservedSpecimen
- **Type status:** Other material. **Occurrence:** recordedBy: Choi, Sei-Woong; individualCount: 1; sex: male; lifeStage: adult; disposition: Mokpo National University; occurrenceID: 8607FF0E-F1F4-5641-A51D-5C8AF00D2292; **Taxon:** scientificName: *Hypenakengkalis*; **Location:** country: South Korea; stateProvince: GW; county: Pyeongchang; locality: Mt. Nambyeong, Gogil-ri; verbatimElevation: 1000; **Identification:** identifiedBy: Sung-Soo Kim; dateIdentified: 2024; **Event:** samplingProtocol: Ultraviolet bucket trap; samplingEffort: 6 trapping hours; eventDate: 8/19/2016; **Record Level:** modified: 3/30/2025; language: en; collectionCode: Insects; basisOfRecord: PreservedSpecimen
- **Type status:** Other material. **Occurrence:** recordedBy: Choi, Sei-Woong; individualCount: 1; sex: male; lifeStage: adult; disposition: Mokpo National University; occurrenceID: 6A9206F6-7EF9-5734-A749-A5A6B0688B6B; **Taxon:** scientificName: *Hypenakengkalis*; **Location:** country: South Korea; stateProvince: GW; county: Wonju; locality: Bangye-ri, Munmak-eup; verbatimCoordinates: 37°19'40.64"N 127°46'4.61“E; **Identification:** identifiedBy: Sung-Soo Kim; dateIdentified: 2024; **Event:** samplingProtocol: Ultraviolet bucket trap; samplingEffort: 6 trapping hours; eventDate: 9/18/2010; **Record Level:** modified: 3/30/2025; language: en; collectionCode: Insects; basisOfRecord: PreservedSpecimen
- **Type status:** Other material. **Occurrence:** recordedBy: Choi, Sei-Woong; individualCount: 1; sex: female; lifeStage: adult; disposition: Mokpo National University; occurrenceID: E31EBCAF-9C08-592D-AC2A-ADABB7A3D0B5; **Taxon:** scientificName: *Hypenakengkalis*; **Location:** country: South Korea; stateProvince: Seoul; county: Hoegi-dong; locality: Kyung Hee Univ.; **Identification:** identifiedBy: Sung-Soo Kim; dateIdentified: 2024; **Event:** samplingProtocol: Ultraviolet bucket trap; samplingEffort: 6 trapping hours; eventDate: 10/17/1992; **Record Level:** modified: 3/30/2025; language: en; collectionCode: Insects; basisOfRecord: PreservedSpecimen
- **Type status:** Other material. **Occurrence:** recordedBy: Choi, Sei-Woong; individualCount: 1; sex: male; lifeStage: adult; disposition: Mokpo National University; occurrenceID: 5F9FF90F-7411-5A74-BF7B-2BCA8BDA8E1D; **Taxon:** scientificName: *Hypenakengkalis*; **Location:** country: South Korea; stateProvince: GB; county: Yeongdeok; **Identification:** identifiedBy: Sung-Soo Kim; dateIdentified: 2024; **Event:** samplingProtocol: Ultraviolet bucket trap; samplingEffort: 6 trapping hours; eventDate: 9/3/2022; **Record Level:** modified: 3/30/2025; language: en; collectionCode: Insects; basisOfRecord: PreservedSpecimen
- **Type status:** Other material. **Occurrence:** recordedBy: Choi, Sei-Woong; individualCount: 1; sex: female; lifeStage: adult; disposition: Mokpo National University; occurrenceID: 7C37829A-C71D-5A67-8674-F3BBAFC251B7; **Taxon:** scientificName: *Hypenakengkalis*; **Location:** country: South Korea; stateProvince: GN; county: Sancheong; locality: Chahwang-myeon; verbatimElevation: 358; verbatimCoordinates: 35°26'36"N 127°56'20“E; **Identification:** identifiedBy: Sei-Woong Choi; dateIdentified: 2024; **Event:** samplingProtocol: Ultraviolet bucket trap; samplingEffort: 6 trapping hours; eventDate: 9/5/2008; **Record Level:** modified: 3/30/2025; language: en; collectionCode: Insects; basisOfRecord: PreservedSpecimen
- **Type status:** Other material. **Occurrence:** recordedBy: Choi, Sei-Woong; individualCount: 1; sex: male; lifeStage: adult; disposition: Mokpo National University; occurrenceID: E9A39D8E-1F9C-5651-9BA4-9ADAE9B2770E; **Taxon:** scientificName: *Hypenakengkalis*; **Location:** country: South Korea; stateProvince: JN; county: Muan; locality: Mt. Seungdal; verbatimCoordinates: 34°54'N 126°27'E; **Identification:** identifiedBy: Sei-Woong Choi; dateIdentified: 2024; **Event:** samplingProtocol: Ultraviolet bucket trap; samplingEffort: 6 trapping hours; eventDate: 11/9/2004; **Record Level:** modified: 3/30/2025; language: en; collectionCode: Insects; basisOfRecord: PreservedSpecimen

##### Distribution

Korea, Japan, China, Russian Far East.

##### Notes

Fig. 4e.

#### Hypena albopunctalis

(Leech, 1889)

EAC9CD22-E20F-5058-9547-1B597B089FDE

##### Distribution

Korea, China, Taiwan.

##### Notes

Leech (1889) first recorded this species in Gensan, North Korea. In Japan, it is known to feed on *Oreocnidefrutescens* (Thunb.) Miq. (Urticaceae), a plant species that is exclusively found in Biyang-do, Jeju Island, Korea. Kononenko and Han (2007) highlighted the ambiguity surrounding records of this species in Korea. Considering the distribution of its food plant and the observations made by Kononenko and Han (2007), the presence of this species in Korea remains uncertain.

#### Hypena stygiana

Butler, 1878

3AD61DF4-A6AE-52E8-8C14-022AC33A3E8A

##### Materials

- **Type status:** Other material. **Occurrence:** recordedBy: Choi, Sei-Woong; individualCount: 1; sex: male; lifeStage: adult; disposition: Mokpo National University; occurrenceID: B1E5EF3A-9CC6-5796-9F80-933FB67F8045; **Taxon:** scientificName: *Hypenastygiana*; **Location:** country: South Korea; stateProvince: GW; county: Yanggu; locality: Wondang-ri, Dong-myeon; **Identification:** identifiedBy: Sung-Soo Kim; dateIdentified: 2024; **Event:** samplingProtocol: Ultraviolet bucket trap; samplingEffort: 6 trapping hours; eventDate: 5/22/2008; **Record Level:** modified: 3/30/2025; language: en; collectionCode: Insects; basisOfRecord: PreservedSpecimen
- **Type status:** Other material. **Occurrence:** recordedBy: Choi, Sei-Woong; individualCount: 1; sex: male; lifeStage: adult; disposition: Mokpo National University; occurrenceID: BC4A7954-7BE8-5907-A84F-3EC25CF93A17; **Taxon:** scientificName: *Hypenastygiana*; **Location:** country: South Korea; stateProvince: GW; county: Inje; locality: Yongdae-ri, Buk-myeon; **Identification:** identifiedBy: Sung-Soo Kim; dateIdentified: 2024; **Event:** samplingProtocol: Ultraviolet bucket trap; samplingEffort: 6 trapping hours; eventDate: 7/18/2023; **Record Level:** modified: 3/30/2025; language: en; collectionCode: Insects; basisOfRecord: PreservedSpecimen
- **Type status:** Other material. **Occurrence:** recordedBy: Choi, Sei-Woong; individualCount: 1; sex: female; lifeStage: adult; disposition: Mokpo National University; occurrenceID: 612B6166-B097-55B8-B6DF-46C7340C9111; **Taxon:** scientificName: *Hypenastygiana*; **Location:** country: South Korea; stateProvince: GW; county: Yangyang; locality: nearJochimnyeong tunnel; **Identification:** identifiedBy: Sung-Soo Kim; dateIdentified: 2024; **Event:** samplingProtocol: Ultraviolet bucket trap; samplingEffort: 6 trapping hours; eventDate: 4/13/2009; **Record Level:** modified: 3/30/2025; language: en; collectionCode: Insects; basisOfRecord: PreservedSpecimen
- **Type status:** Other material. **Occurrence:** recordedBy: Choi, Sei-Woong; individualCount: 1; sex: male; lifeStage: adult; disposition: Mokpo National University; occurrenceID: BD9CC0E3-B34B-5E5C-8EC6-EC74EAB6E475; **Taxon:** scientificName: *Hypenastygiana*; **Location:** country: South Korea; stateProvince: GW; county: Pyeongchang; locality: Mt.Jungwang,Daehwa-myeon; **Identification:** identifiedBy: Sung-Soo Kim; dateIdentified: 2024; **Event:** samplingProtocol: Ultraviolet bucket trap; samplingEffort: 6 trapping hours; eventDate: 7/1/2011; **Record Level:** modified: 3/30/2025; language: en; collectionCode: Insects; basisOfRecord: PreservedSpecimen
- **Type status:** Other material. **Occurrence:** recordedBy: Choi, Sei-Woong; individualCount: 1; sex: male; lifeStage: adult; disposition: Mokpo National University; occurrenceID: D3144C15-98C0-5A1F-8FD0-A2A0C368A9AB; **Taxon:** scientificName: *Hypenastygiana*; **Location:** country: South Korea; stateProvince: GW; county: Jeongseon; locality: Yeoryang-myeon; **Identification:** identifiedBy: Sung-Soo Kim; dateIdentified: 2024; **Event:** samplingProtocol: Ultraviolet bucket trap; samplingEffort: 6 trapping hours; eventDate: 5/11/2018; **Record Level:** modified: 3/30/2025; language: en; collectionCode: Insects; basisOfRecord: PreservedSpecimen
- **Type status:** Other material. **Occurrence:** recordedBy: Choi, Sei-Woong; individualCount: 1; sex: male; lifeStage: adult; disposition: Mokpo National University; occurrenceID: 3F68EA41-2F14-540D-99C5-7B181279E0A0; **Taxon:** scientificName: *Hypenastygiana*; **Location:** country: South Korea; stateProvince: GW; county: Yeongwol; locality: Mt. Hamback; **Identification:** identifiedBy: Sung-Soo Kim; dateIdentified: 2024; **Event:** samplingProtocol: Ultraviolet bucket trap; samplingEffort: 6 trapping hours; eventDate: 5/5/2012; **Record Level:** modified: 3/30/2025; language: en; collectionCode: Insects; basisOfRecord: PreservedSpecimen
- **Type status:** Other material. **Occurrence:** recordedBy: Choi, Sei-Woong; individualCount: 1; sex: female; lifeStage: adult; disposition: Mokpo National University; occurrenceID: 6C6D4826-20F3-502F-9E12-2AC2241786AB; **Taxon:** scientificName: *Hypenastygiana*; **Location:** country: South Korea; stateProvince: GW; county: Yeongwol; locality: Mt. Hamback; **Identification:** identifiedBy: Sung-Soo Kim; dateIdentified: 2024; **Event:** samplingProtocol: Ultraviolet bucket trap; samplingEffort: 6 trapping hours; eventDate: 5/5/2012; **Record Level:** modified: 3/30/2025; language: en; collectionCode: Insects; basisOfRecord: PreservedSpecimen
- **Type status:** Other material. **Occurrence:** recordedBy: Choi, Sei-Woong; individualCount: 2; sex: males; lifeStage: adult; disposition: Mokpo National University; occurrenceID: FEB1145D-9A85-5D1A-A56E-E3DC1264304F; **Taxon:** scientificName: *Hypenastygiana*; **Location:** country: South Korea; stateProvince: GW; county: Yeongwol; locality: Ssangyong-ri, Hanbando-myeon; **Identification:** identifiedBy: Sung-Soo Kim; dateIdentified: 2024; **Event:** samplingProtocol: Ultraviolet bucket trap; samplingEffort: 6 trapping hours; eventDate: 5/27/2022; **Record Level:** modified: 3/30/2025; language: en; collectionCode: Insects; basisOfRecord: PreservedSpecimen
- **Type status:** Other material. **Occurrence:** recordedBy: Choi, Sei-Woong; individualCount: 1; sex: female; lifeStage: adult; disposition: Mokpo National University; occurrenceID: 42F00D63-E192-544E-B2A3-2A3BC5913BB1; **Taxon:** scientificName: *Hypenastygiana*; **Location:** country: South Korea; stateProvince: GG; county: Namyangju; locality: Mt. Chungnyeong, Sudong-myeon; **Identification:** identifiedBy: Sung-Soo Kim; dateIdentified: 2024; **Event:** samplingProtocol: Ultraviolet bucket trap; samplingEffort: 6 trapping hours; eventDate: 7/23/2016; **Record Level:** modified: 3/30/2025; language: en; collectionCode: Insects; basisOfRecord: PreservedSpecimen
- **Type status:** Other material. **Occurrence:** recordedBy: Choi, Sei-Woong; individualCount: 1; sex: female; lifeStage: adult; disposition: Mokpo National University; occurrenceID: 10A8B81C-A007-529E-8F79-209AB3E94EBE; **Taxon:** scientificName: *Hypenastygiana*; **Location:** country: South Korea; stateProvince: CB; county: Jecheon; locality: Jeongok-ri, Susan-myeon; **Identification:** identifiedBy: Sung-Soo Kim; dateIdentified: 2024; **Event:** samplingProtocol: Ultraviolet bucket trap; samplingEffort: 6 trapping hours; eventDate: 7/8/2018; **Record Level:** modified: 3/30/2025; language: en; collectionCode: Insects; basisOfRecord: PreservedSpecimen
- **Type status:** Other material. **Occurrence:** recordedBy: Choi, Sei-Woong; individualCount: 1; sex: male; lifeStage: adult; disposition: Mokpo National University; occurrenceID: 94BD85C0-4964-5BA8-B4C3-A4996EDFD1EE; **Taxon:** scientificName: *Hypenastygiana*; **Location:** country: South Korea; stateProvince: GN; county: Hamyang; locality: Samjeong-ri, Macheon-myeon; **Identification:** identifiedBy: Sung-Soo Kim; dateIdentified: 2024; **Event:** samplingProtocol: Ultraviolet bucket trap; samplingEffort: 6 trapping hours; eventDate: 6/2/2015; **Record Level:** modified: 3/30/2025; language: en; collectionCode: Insects; basisOfRecord: PreservedSpecimen
- **Type status:** Other material. **Occurrence:** recordedBy: Choi, Sei-Woong; individualCount: 1; sex: female; lifeStage: adult; disposition: Mokpo National University; occurrenceID: 6EAFC65A-EC2F-5C66-8852-C10C01444214; **Taxon:** scientificName: *Hypenastygiana*; **Location:** country: South Korea; stateProvince: JN; county: Gurye; locality: Mt. Jiri,Toji-myeon; verbatimElevation: 1362; verbatimCoordinates: 35°18'2"N 127°33'10.5“E; **Identification:** identifiedBy: Sei-Woong Choi; dateIdentified: 2024; **Event:** samplingProtocol: Ultraviolet bucket trap; samplingEffort: 6 trapping hours; eventDate: 6/19/2020; **Record Level:** modified: 3/30/2025; language: en; collectionCode: Insects; basisOfRecord: PreservedSpecimen
- **Type status:** Other material. **Occurrence:** recordedBy: Choi, Sei-Woong; individualCount: 1; sex: male; lifeStage: adult; disposition: Mokpo National University; occurrenceID: 482594AF-6405-5E8D-8E59-E92705465673; **Taxon:** scientificName: *Hypenastygiana*; **Location:** country: South Korea; stateProvince: JN; county: Gurye; locality: Mt. Jiri,Toji-myeon; verbatimElevation: 1362; verbatimCoordinates: 35°18'2"N 127°33'10.5“E; **Identification:** identifiedBy: Sei-Woong Choi; dateIdentified: 2024; **Event:** samplingProtocol: Ultraviolet bucket trap; samplingEffort: 6 trapping hours; eventDate: 6/19/2020; **Record Level:** modified: 3/30/2025; language: en; collectionCode: Insects; basisOfRecord: PreservedSpecimen
- **Type status:** Other material. **Occurrence:** recordedBy: Choi, Sei-Woong; individualCount: 1; sex: female; lifeStage: adult; disposition: Mokpo National University; occurrenceID: 98289FFD-87BB-5556-9A86-DD0C374EFAC4; **Taxon:** scientificName: *Hypenastygiana*; **Location:** country: South Korea; stateProvince: JN; county: Gurye; locality: Mt.Jiri, Toji-myeon; verbatimElevation: 1371; verbatimCoordinates: 35°18'01"N 127°33'09“E; **Identification:** identifiedBy: Sei-Woong Choi; dateIdentified: 2024; **Event:** samplingProtocol: Ultraviolet bucket trap; samplingEffort: 6 trapping hours; eventDate: 8/11/2016; **Record Level:** modified: 3/30/2025; language: en; collectionCode: Insects; basisOfRecord: PreservedSpecimen
- **Type status:** Other material. **Occurrence:** recordedBy: Choi, Sei-Woong; individualCount: 1; sex: female; lifeStage: adult; disposition: Mokpo National University; occurrenceID: 2BDDEE00-A786-5F1A-A211-A97F6F74E8A6; **Taxon:** scientificName: *Hypenastygiana*; **Location:** country: South Korea; stateProvince: JN; county: Gurye; locality: Mt. Jiri,Sandong-myeon; verbatimElevation: 1504; verbatimCoordinates: 35°17'37.8"N 127°31'58.5“E; **Identification:** identifiedBy: Sei-Woong Choi; dateIdentified: 2024; **Event:** samplingProtocol: Ultraviolet bucket trap; samplingEffort: 6 trapping hours; eventDate: 6/9/2023; **Record Level:** modified: 3/30/2025; language: en; collectionCode: Insects; basisOfRecord: PreservedSpecimen
- **Type status:** Other material. **Occurrence:** recordedBy: Choi, Sei-Woong; individualCount: 1; sex: male; lifeStage: adult; disposition: Mokpo National University; occurrenceID: 9FD5E0E0-C63E-50F9-9652-0D4C3D6C386D; **Taxon:** scientificName: *Hypenastygiana*; **Location:** country: South Korea; stateProvince: JN; county: Goheung; locality: Sangnam-ri, Daeseo-myeon; **Identification:** identifiedBy: Sung-Soo Kim; dateIdentified: 2024; **Event:** samplingProtocol: Ultraviolet bucket trap; samplingEffort: 6 trapping hours; eventDate: 6/17/2022; **Record Level:** modified: 3/30/2025; language: en; collectionCode: Insects; basisOfRecord: PreservedSpecimen
- **Type status:** Other material. **Occurrence:** recordedBy: Choi, Sei-Woong; individualCount: 1; sex: female; lifeStage: adult; disposition: Mokpo National University; occurrenceID: F945D0BE-FDDC-5204-A0F8-10C5B75C6A63; **Taxon:** scientificName: *Hypenastygiana*; **Location:** country: South Korea; stateProvince: JN; county: Goheung; locality: Sangnam-ri, Daeseo-myeon; **Identification:** identifiedBy: Sung-Soo Kim; dateIdentified: 2024; **Event:** samplingProtocol: Ultraviolet bucket trap; samplingEffort: 6 trapping hours; eventDate: 6/17/2022; **Record Level:** modified: 3/30/2025; language: en; collectionCode: Insects; basisOfRecord: PreservedSpecimen
- **Type status:** Other material. **Occurrence:** recordedBy: Choi, Sei-Woong; individualCount: 1; sex: male; lifeStage: adult; disposition: Mokpo National University; occurrenceID: 4AFF78B0-6B6E-5445-BE3B-D57BEBDBF835; **Taxon:** scientificName: *Hypenastygiana*; **Location:** country: South Korea; stateProvince: JJ; county: Seogwipo; locality: Jeju International Univ., Hawon-dong; **Identification:** identifiedBy: Sung-Soo Kim; dateIdentified: 2024; **Event:** samplingProtocol: Ultraviolet bucket trap; samplingEffort: 6 trapping hours; eventDate: 7/13/2015; **Record Level:** modified: 3/30/2025; language: en; collectionCode: Insects; basisOfRecord: PreservedSpecimen
- **Type status:** Other material. **Occurrence:** recordedBy: Choi, Sei-Woong; individualCount: 1; sex: female; lifeStage: adult; disposition: Mokpo National University; occurrenceID: AE76B653-218A-5D00-A11C-A7BCA6AF0B57; **Taxon:** scientificName: *Hypenastygiana*; **Location:** country: South Korea; stateProvince: JJ; county: Seogwipo; locality: Jeju International Univ., Hawon-dong; **Identification:** identifiedBy: Sung-Soo Kim; dateIdentified: 2024; **Event:** samplingProtocol: Ultraviolet bucket trap; samplingEffort: 6 trapping hours; eventDate: 7/13/2015; **Record Level:** modified: 3/30/2025; language: en; collectionCode: Insects; basisOfRecord: PreservedSpecimen

##### Distribution

Korea, Japan, Russian Far East.

##### Notes

*Hypenastygiana* (GenBank accession No. PV274475, PV274482) showed the highest mean genetic distance of 8.52% from *H.pulverulenta*, while it exhibited the lowest mean genetic distance of 4.25% from *H.squalida* (Fig. 4f).

#### Hypena squalida

Butler, 1879

051EC568-C774-5A60-B4E2-61F4A8B2878E

##### Materials

- **Type status:** Other material. **Occurrence:** recordedBy: Choi, Sei-Woong; individualCount: 1; sex: female; lifeStage: adult; disposition: Mokpo National University; occurrenceID: 2B2E0B7F-A418-5789-B8EF-996246FD8D5D; **Taxon:** scientificName: *Hypenasqualida*; **Location:** country: South Korea; stateProvince: GW; county: Pyeongchang; locality: Singi-ri, Jinbu-myeon; **Identification:** identifiedBy: Sung-Soo Kim; dateIdentified: 2024; **Event:** samplingProtocol: Ultraviolet bucket trap; samplingEffort: 6 trapping hours; eventDate: 7/5/2021; **Record Level:** modified: 3/30/2025; language: en; collectionCode: Insects; basisOfRecord: PreservedSpecimen
- **Type status:** Other material. **Occurrence:** recordedBy: Choi, Sei-Woong; individualCount: 1; sex: female; lifeStage: adult; disposition: Mokpo National University; occurrenceID: C1B38E33-35FD-599F-8207-834B1190020F; **Taxon:** scientificName: *Hypenasqualida*; **Location:** country: South Korea; stateProvince: GW; county: Taebaek; locality: near RailwayStation Chugeon; **Identification:** identifiedBy: Sung-Soo Kim; dateIdentified: 2024; **Event:** samplingProtocol: Ultraviolet bucket trap; samplingEffort: 6 trapping hours; eventDate: 7/7/2017; **Record Level:** modified: 3/30/2025; language: en; collectionCode: Insects; basisOfRecord: PreservedSpecimen
- **Type status:** Other material. **Occurrence:** recordedBy: Choi, Sei-Woong; individualCount: 1; sex: male; lifeStage: adult; disposition: Mokpo National University; occurrenceID: 2D2201F7-853E-5D64-8AB4-7B230E4D2904; **Taxon:** scientificName: *Hypenasqualida*; **Location:** country: South Korea; stateProvince: GB; county: Ulleung; locality: Na-ri, Buk-myeon; **Identification:** identifiedBy: Sung-Soo Kim; dateIdentified: 2024; **Event:** samplingProtocol: Ultraviolet bucket trap; samplingEffort: 6 trapping hours; eventDate: 7/23/1998; **Record Level:** modified: 3/30/2025; language: en; collectionCode: Insects; basisOfRecord: PreservedSpecimen
- **Type status:** Other material. **Occurrence:** recordedBy: Choi, Sei-Woong; individualCount: 4; sex: female; lifeStage: adult; disposition: Mokpo National University; occurrenceID: F2A1EB4A-7BA6-5912-9419-A72B944A9B93; **Taxon:** scientificName: *Hypenasqualida*; **Location:** country: South Korea; stateProvince: GB; county: Ulleung; locality: Na-ri, Buk-myeon; **Identification:** identifiedBy: Sung-Soo Kim; dateIdentified: 2024; **Event:** samplingProtocol: Ultraviolet bucket trap; samplingEffort: 6 trapping hours; eventDate: 7/23/1998; **Record Level:** modified: 3/30/2025; language: en; collectionCode: Insects; basisOfRecord: PreservedSpecimen

##### Distribution

Korea, Japan, Russian Far East.

##### Notes

*Hypenasqualida* (GenBank accession No. PV274473) showed the highest mean genetic distance of 9.06% from *H.obacerralis*, while it exhibited the lowest mean genetic distance of 4.25% from *H.stygiana* (Fig. 5a).

#### Hypena nigrobasalis

(Herz, 1904)

42C0BD34-6A85-5413-A0C0-A6B0A401A20B

##### Materials

- **Type status:** Other material. **Occurrence:** recordedBy: Choi, Sei-Woong; individualCount: 1; sex: male; lifeStage: adult; disposition: Mokpo National University; occurrenceID: 1E8336C5-1D49-5422-9FC9-701594F364D6; **Taxon:** scientificName: *Hypenanigrobasalis*; **Location:** country: South Korea; stateProvince: GW; county: Yeongwol; locality: Mt. Saja, Suju-myeon; **Identification:** identifiedBy: Sung-Soo Kim; dateIdentified: 2024; **Event:** samplingProtocol: Ultraviolet bucket trap; samplingEffort: 6 trapping hours; eventDate: 5/24/2011; **Record Level:** modified: 3/30/2025; language: en; collectionCode: Insects; basisOfRecord: PreservedSpecimen
- **Type status:** Other material. **Occurrence:** recordedBy: Choi, Sei-Woong; individualCount: 1; sex: female; lifeStage: adult; disposition: Mokpo National University; occurrenceID: 39075E1C-1571-5896-A505-7522FEB6B387; **Taxon:** scientificName: *Hypenanigrobasalis*; **Location:** country: South Korea; stateProvince: GW; county: Yeongwol; locality: Mt. Saja, Suju-myeon; **Identification:** identifiedBy: Sung-Soo Kim; dateIdentified: 2024; **Event:** samplingProtocol: Ultraviolet bucket trap; samplingEffort: 6 trapping hours; eventDate: 8/5/2011; **Record Level:** modified: 3/30/2025; language: en; collectionCode: Insects; basisOfRecord: PreservedSpecimen
- **Type status:** Other material. **Occurrence:** recordedBy: Choi, Sei-Woong; individualCount: 1; sex: female; lifeStage: adult; disposition: Mokpo National University; occurrenceID: 6E6306A6-AFCA-5380-920E-23389C488B59; **Taxon:** scientificName: *Hypenanigrobasalis*; **Location:** country: South Korea; stateProvince: CN; county: Gongju; locality: Mt. Gyeryong, Gyeryong-myeon; **Identification:** identifiedBy: Sung-Soo Kim; dateIdentified: 2024; **Event:** samplingProtocol: Ultraviolet bucket trap; samplingEffort: 6 trapping hours; eventDate: 6/4/2011; **Record Level:** modified: 3/30/2025; language: en; collectionCode: Insects; basisOfRecord: PreservedSpecimen
- **Type status:** Other material. **Occurrence:** recordedBy: Choi, Sei-Woong; individualCount: 1; sex: female; lifeStage: adult; disposition: Mokpo National University; occurrenceID: 504C0ABF-A565-5AE4-B8C1-C67176BEFF39; **Taxon:** scientificName: *Hypenanigrobasalis*; **Location:** country: South Korea; stateProvince: GB; county: Sangju; **Identification:** identifiedBy: Sung-Soo Kim; dateIdentified: 2024; **Event:** samplingProtocol: Ultraviolet bucket trap; samplingEffort: 6 trapping hours; eventDate: 6/20/2020; **Record Level:** modified: 3/30/2025; language: en; collectionCode: Insects; basisOfRecord: PreservedSpecimen
- **Type status:** Other material. **Occurrence:** recordedBy: Choi, Sei-Woong; individualCount: 1; sex: male; lifeStage: adult; disposition: Mokpo National University; occurrenceID: 1D6E688D-8350-5501-840C-0200EC6DB21E; **Taxon:** scientificName: *Hypenanigrobasalis*; **Location:** country: South Korea; stateProvince: GN; county: Geoje; locality: Gucheon-ri, Dongbu-myeon; **Identification:** identifiedBy: Sung-Soo Kim; dateIdentified: 2024; **Event:** samplingProtocol: Ultraviolet bucket trap; samplingEffort: 6 trapping hours; eventDate: 8/25/2012; **Record Level:** modified: 3/30/2025; language: en; collectionCode: Insects; basisOfRecord: PreservedSpecimen
- **Type status:** Other material. **Occurrence:** recordedBy: Choi, Sei-Woong; individualCount: 1; sex: male; lifeStage: adult; disposition: Mokpo National University; occurrenceID: 299A2044-9E06-5453-AA1F-10AFD0A3D7A8; **Taxon:** scientificName: *Hypenanigrobasalis*; **Location:** country: South Korea; stateProvince: GN; county: Geoje; **Identification:** identifiedBy: Sung-Soo Kim; dateIdentified: 2024; **Event:** samplingProtocol: Ultraviolet bucket trap; samplingEffort: 6 trapping hours; eventDate: 8/19/2012; **Record Level:** modified: 3/30/2025; language: en; collectionCode: Insects; basisOfRecord: PreservedSpecimen
- **Type status:** Other material. **Occurrence:** recordedBy: Choi, Sei-Woong; individualCount: 1; sex: male; lifeStage: adult; disposition: Mokpo National University; occurrenceID: F292A12A-89A7-532A-8685-9F1C93EB8945; **Taxon:** scientificName: *Hypenanigrobasalis*; **Location:** country: South Korea; stateProvince: JN; county: Gurye; locality: Mt. Jiri, Sandong-myeon; verbatimElevation: 923; verbatimCoordinates: 35°19'22"N 127°31'23“E; **Identification:** identifiedBy: Sei-Woong Choi; dateIdentified: 2024; **Event:** samplingProtocol: Ultraviolet bucket trap; samplingEffort: 6 trapping hours; eventDate: 5/22/2008; **Record Level:** modified: 3/30/2025; language: en; collectionCode: Insects; basisOfRecord: PreservedSpecimen
- **Type status:** Other material. **Occurrence:** recordedBy: Choi, Sei-Woong; individualCount: 1; sex: female; lifeStage: adult; disposition: Mokpo National University; occurrenceID: 9EE04DF5-5521-5983-92D9-0FFC63C02493; **Taxon:** scientificName: *Hypenanigrobasalis*; **Location:** country: South Korea; stateProvince: JN; county: Gurye; locality: Mt. Jiri, Toji-myeon; verbatimElevation: 1371; verbatimCoordinates: 35°18'01"N 127°33'09“E; **Identification:** identifiedBy: Sei-Woong Choi; dateIdentified: 2024; **Event:** samplingProtocol: Ultraviolet bucket trap; samplingEffort: 6 trapping hours; eventDate: 7/11/2018; **Record Level:** modified: 3/30/2025; language: en; collectionCode: Insects; basisOfRecord: PreservedSpecimen
- **Type status:** Other material. **Occurrence:** recordedBy: Choi, Sei-Woong; individualCount: 1; sex: male; lifeStage: adult; disposition: Mokpo National University; occurrenceID: 28DABF6F-ECF1-5E5D-85FF-2DC09E2D4606; **Taxon:** scientificName: *Hypenanigrobasalis*; **Location:** country: South Korea; stateProvince: JN; county: Muan; locality: Dalsan-ri, Mongtan-myeon; verbatimElevation: 261; verbatimCoordinates: 34°55'00"N 126°27'18“E; **Identification:** identifiedBy: Sei-Woong Choi; dateIdentified: 2024; **Event:** samplingProtocol: Ultraviolet bucket trap; samplingEffort: 6 trapping hours; eventDate: 4/29/2017; **Record Level:** modified: 3/30/2025; language: en; collectionCode: Insects; basisOfRecord: PreservedSpecimen
- **Type status:** Other material. **Occurrence:** recordedBy: Choi, Sei-Woong; individualCount: 2; sex: males; lifeStage: adult; disposition: Mokpo National University; occurrenceID: 9917EF4D-CC7B-5214-A6A3-11587E9E273F; **Taxon:** scientificName: *Hypenanigrobasalis*; **Location:** country: South Korea; stateProvince: JN; county: Muan; locality: Mt. Bopyeong, Muan-eup; **Identification:** identifiedBy: Sung-Soo Kim; dateIdentified: 2024; **Event:** samplingProtocol: Ultraviolet bucket trap; samplingEffort: 6 trapping hours; eventDate: 5/20/2018; **Record Level:** modified: 3/30/2025; language: en; collectionCode: Insects; basisOfRecord: PreservedSpecimen
- **Type status:** Other material. **Occurrence:** recordedBy: Choi, Sei-Woong; individualCount: 1; sex: female; lifeStage: adult; disposition: Mokpo National University; occurrenceID: 9C1F425B-4F54-5FC1-85E9-989DA832243E; **Taxon:** scientificName: *Hypenanigrobasalis*; **Location:** country: South Korea; stateProvince: JN; county: Yeongam; locality: Mt. Wolchul,; verbatimElevation: 180; verbatimCoordinates: 34°44'N 126°41'E; **Identification:** identifiedBy: Sei-Woong Choi; dateIdentified: 2024; **Event:** samplingProtocol: Ultraviolet bucket trap; samplingEffort: 6 trapping hours; eventDate: 5/25/2006; **Record Level:** modified: 3/30/2025; language: en; collectionCode: Insects; basisOfRecord: PreservedSpecimen

##### Distribution

Korea, Japan, China (Jilin), Russia.

##### Notes

*Hypenanigrobasalis* (GenBank accession No. PV274476, PV274477, PV274478, PV274480) showed the highest mean genetic distance of 8.1% from *H.pulverulenta*, while it exhibited the lowest mean genetic distance of 4.64% from *H.stygiana* (Fig. 5b).

#### Hypena zilla

(Butler, 1879)

33DABFEF-E0F2-5D18-9C8D-D98BF63A34B8

##### Materials

- **Type status:** Other material. **Occurrence:** recordedBy: Choi, Sei-Woong; individualCount: 1; sex: male; lifeStage: adult; disposition: Mokpo National University; occurrenceID: 19E30EB8-6054-5A8A-9A1D-7EC90B902C39; **Taxon:** scientificName: *Hypenazilla*; **Location:** country: South Korea; stateProvince: GW; county: Inje; locality: Yongdae-ri, Buk-myeon; **Identification:** identifiedBy: Sung-Soo Kim; dateIdentified: 2024; **Event:** samplingProtocol: Ultraviolet bucket trap; samplingEffort: 6 trapping hours; eventDate: 8/30/2023; **Record Level:** modified: 3/30/2025; language: en; collectionCode: Insects; basisOfRecord: PreservedSpecimen
- **Type status:** Other material. **Occurrence:** recordedBy: Choi, Sei-Woong; individualCount: 1; sex: male; lifeStage: adult; disposition: Mokpo National University; occurrenceID: 435C06C6-D92C-5F5B-B201-BC763DF46250; **Taxon:** scientificName: *Hypenazilla*; **Location:** country: South Korea; stateProvince: GG; county: Namyangju; locality: Mt. Chungnyeong, Sudong-myeon; **Identification:** identifiedBy: Sung-Soo Kim; dateIdentified: 2024; **Event:** samplingProtocol: Ultraviolet bucket trap; samplingEffort: 6 trapping hours; eventDate: 5/23/2016; **Record Level:** modified: 3/30/2025; language: en; collectionCode: Insects; basisOfRecord: PreservedSpecimen
- **Type status:** Other material. **Occurrence:** recordedBy: Choi, Sei-Woong; individualCount: 1; sex: female; lifeStage: adult; disposition: Mokpo National University; occurrenceID: CB70C924-4E27-5264-A16F-9501EBBB0502; **Taxon:** scientificName: *Hypenazilla*; **Location:** country: South Korea; stateProvince: GG; county: Namyangju; locality: Mt. Chungnyeong, Sudong-myeon; **Identification:** identifiedBy: Sung-Soo Kim; dateIdentified: 2024; **Event:** samplingProtocol: Ultraviolet bucket trap; samplingEffort: 6 trapping hours; eventDate: 5/23/2016; **Record Level:** modified: 3/30/2025; language: en; collectionCode: Insects; basisOfRecord: PreservedSpecimen
- **Type status:** Other material. **Occurrence:** recordedBy: Choi, Sei-Woong; individualCount: 1; sex: female; lifeStage: adult; disposition: Mokpo National University; occurrenceID: 7B440511-3980-5AA0-8839-D2A48AFD9FDA; **Taxon:** scientificName: *Hypenazilla*; **Location:** country: South Korea; stateProvince: GG; county: Yongin; locality: Cheongdeok-dong, Giheung-gu; **Identification:** identifiedBy: Sung-Soo Kim; dateIdentified: 2024; **Event:** samplingProtocol: Ultraviolet bucket trap; samplingEffort: 6 trapping hours; eventDate: 4/29/2014; **Record Level:** modified: 3/30/2025; language: en; collectionCode: Insects; basisOfRecord: PreservedSpecimen
- **Type status:** Other material. **Occurrence:** recordedBy: Choi, Sei-Woong; individualCount: 1; sex: female; lifeStage: adult; disposition: Mokpo National University; occurrenceID: 2A9B716C-050E-5BC1-8104-491EEE88A89A; **Taxon:** scientificName: *Hypenazilla*; **Location:** country: South Korea; stateProvince: CB; county: Goesan; locality: Hwayang-ri, Cheongcheon-myeon; **Identification:** identifiedBy: Sung-Soo Kim; dateIdentified: 2024; **Event:** samplingProtocol: Ultraviolet bucket trap; samplingEffort: 6 trapping hours; eventDate: 8/23/2014; **Record Level:** modified: 3/30/2025; language: en; collectionCode: Insects; basisOfRecord: PreservedSpecimen
- **Type status:** Other material. **Occurrence:** recordedBy: Choi, Sei-Woong; individualCount: 1; sex: female; lifeStage: adult; disposition: Mokpo National University; occurrenceID: EF4CC753-1511-55B3-BB9F-27E2F7CF7458; **Taxon:** scientificName: *Hypenazilla*; **Location:** country: South Korea; stateProvince: CB; county: Jincheon; locality: Singye-ri, Iwol-myeon; **Identification:** identifiedBy: Sung-Soo Kim; dateIdentified: 2024; **Event:** samplingProtocol: Ultraviolet bucket trap; samplingEffort: 6 trapping hours; eventDate: 6/15/2019; **Record Level:** modified: 3/30/2025; language: en; collectionCode: Insects; basisOfRecord: PreservedSpecimen
- **Type status:** Other material. **Occurrence:** recordedBy: Choi, Sei-Woong; individualCount: 1; sex: male; lifeStage: adult; disposition: Mokpo National University; occurrenceID: 40F595C8-3652-5661-ACD1-206CA6B83D64; **Taxon:** scientificName: *Hypenazilla*; **Location:** country: South Korea; stateProvince: GB; county: Yeongyang; locality: Suha-ri, Subi-myeon; verbatimElevation: 338; verbatimCoordinates: 36°49'44"N 129°15'53“E; **Identification:** identifiedBy: Sei-Woong Choi; dateIdentified: 2024; **Event:** samplingProtocol: Ultraviolet bucket trap; samplingEffort: 6 trapping hours; eventDate: 6/29/2007; **Record Level:** modified: 3/30/2025; language: en; collectionCode: Insects; basisOfRecord: PreservedSpecimen
- **Type status:** Other material. **Occurrence:** recordedBy: Choi, Sei-Woong; individualCount: 1; sex: female; lifeStage: adult; disposition: Mokpo National University; occurrenceID: 3E40C821-ED14-54C5-AA82-03303E2452A9; **Taxon:** scientificName: *Hypenazilla*; **Location:** country: South Korea; stateProvince: JN; county: Yeongam; locality: Mt. Wolchul; verbatimElevation: 180; verbatimCoordinates: 34°44'N 126°41'E; **Identification:** identifiedBy: Sei-Woong Choi; dateIdentified: 2024; **Event:** samplingProtocol: Ultraviolet bucket trap; samplingEffort: 6 trapping hours; eventDate: 5/25/2006; **Record Level:** modified: 3/30/2025; language: en; collectionCode: Insects; basisOfRecord: PreservedSpecimen
- **Type status:** Other material. **Occurrence:** recordedBy: Choi, Sei-Woong; individualCount: 1; sex: female; lifeStage: adult; disposition: Mokpo National University; occurrenceID: AB2E6B63-DE64-5D72-9F79-21EEA1BB15DF; **Taxon:** scientificName: *Hypenazilla*; **Location:** country: South Korea; stateProvince: JN; county: Sinan; locality: Aphae-eup; **Identification:** identifiedBy: Sung-Soo Kim; dateIdentified: 2024; **Event:** samplingProtocol: Ultraviolet bucket trap; samplingEffort: 6 trapping hours; eventDate: 5/15/2018; **Record Level:** modified: 3/30/2025; language: en; collectionCode: Insects; basisOfRecord: PreservedSpecimen
- **Type status:** Other material. **Occurrence:** recordedBy: Choi, Sei-Woong; individualCount: 1; sex: female; lifeStage: adult; disposition: Mokpo National University; occurrenceID: DE6D1F38-F02C-5969-AC1D-8637AB1BE509; **Taxon:** scientificName: *Hypenazilla*; **Location:** country: South Korea; stateProvince: JJ; county: Jeju; locality: Mt. Halla, Aewol-eup; verbatimElevation: 1694; verbatimCoordinates: 33°21'44"N 126°31'10"E; **Identification:** identifiedBy: Sei-Woong Choi; dateIdentified: 2024; **Event:** samplingProtocol: Ultraviolet bucket trap; samplingEffort: 6 trapping hours; eventDate: 7/8/2018; **Record Level:** modified: 3/30/2025; language: en; collectionCode: Insects; basisOfRecord: PreservedSpecimen
- **Type status:** Other material. **Occurrence:** recordedBy: Choi, Sei-Woong; individualCount: 1; sex: male; lifeStage: adult; disposition: Mokpo National University; occurrenceID: D2C0FF2C-63CD-5809-83FE-8B7A8EFE4819; **Taxon:** scientificName: *Hypenazilla*; **Location:** country: South Korea; stateProvince: JJ; county: Jeju; locality: Aewol-eup; verbatimElevation: 1410; verbatimCoordinates: 33°22'32.2"N 126°29'58.8"E; **Identification:** identifiedBy: Sei-Woong Choi; dateIdentified: 2024; **Event:** samplingProtocol: Ultraviolet bucket trap; samplingEffort: 6 trapping hours; eventDate: 6/30/2022; **Record Level:** modified: 3/30/2025; language: en; collectionCode: Insects; basisOfRecord: PreservedSpecimen
- **Type status:** Other material. **Occurrence:** recordedBy: Choi, Sei-Woong; individualCount: 1; sex: male; lifeStage: adult; disposition: Mokpo National University; occurrenceID: 87011482-DC39-570E-9C24-C073E7E95E6D; **Taxon:** scientificName: *Hypenazilla*; **Location:** country: South Korea; stateProvince: JJ; county: Jeju; locality: Mt. Halla, Aewol-eup; verbatimElevation: 1694; verbatimCoordinates: 33°21'44"N 126°31'10"E; **Identification:** identifiedBy: Sei-Woong Choi; dateIdentified: 2024; **Event:** samplingProtocol: Ultraviolet bucket trap; samplingEffort: 6 trapping hours; eventDate: 7/4/2019; **Record Level:** modified: 3/30/2025; language: en; collectionCode: Insects; basisOfRecord: PreservedSpecimen
- **Type status:** Other material. **Occurrence:** recordedBy: Choi, Sei-Woong; individualCount: 1; sex: male; lifeStage: adult; disposition: Mokpo National University; occurrenceID: 5B1006F8-4261-575F-B738-E01C94F3AB30; **Taxon:** scientificName: *Hypenazilla*; **Location:** country: South Korea; stateProvince: JJ; county: Seogwipo; locality: Mt. Halla, Hawon-dong; verbatimElevation: 970; verbatimCoordinates: 33°20'N 126°28'E; **Identification:** identifiedBy: Sei-Woong Choi; dateIdentified: 2024; **Event:** samplingProtocol: Ultraviolet bucket trap; samplingEffort: 6 trapping hours; eventDate: 8/26/2005; **Record Level:** modified: 3/30/2025; language: en; collectionCode: Insects; basisOfRecord: PreservedSpecimen
- **Type status:** Other material. **Occurrence:** recordedBy: Choi, Sei-Woong; individualCount: 2; sex: females; lifeStage: adult; disposition: Mokpo National University; occurrenceID: 556F8B2F-327D-5363-A7DF-14778180E67C; **Taxon:** scientificName: *Hypenazilla*; **Location:** country: South Korea; stateProvince: JJ; county: Jeju; locality: Mt. Halla,; verbatimElevation: 1700; **Identification:** identifiedBy: Sung-Soo Kim; dateIdentified: 2024; **Event:** samplingProtocol: Ultraviolet bucket trap; samplingEffort: 6 trapping hours; eventDate: 7/18/2009; **Record Level:** modified: 3/30/2025; language: en; collectionCode: Insects; basisOfRecord: PreservedSpecimen

##### Distribution

Korea, Japan, China.

##### Notes

Fig. 5c.

#### Hypena rivuligera

(Butler, 1881)

34975832-9672-545A-80AD-6342D025B83B

##### Materials

- **Type status:** Other material. **Occurrence:** recordedBy: Choi, Sei-Woong; individualCount: 1; sex: female; lifeStage: adult; disposition: Mokpo National University; occurrenceID: 223C4441-84DE-55A0-B4BA-4B68562B6A7A; **Taxon:** scientificName: *Hypenarivuligera*; **Location:** country: South Korea; stateProvince: GG; county: Gapyeong; locality: Gugmangbong, Buk-myeon; **Identification:** identifiedBy: Sung-Soo Kim; dateIdentified: 2024; **Event:** samplingProtocol: Ultraviolet bucket trap; samplingEffort: 6 trapping hours; eventDate: 6/14/2014; **Record Level:** modified: 3/30/2025; language: en; collectionCode: Insects; basisOfRecord: PreservedSpecimen
- **Type status:** Other material. **Occurrence:** recordedBy: Choi, Sei-Woong; individualCount: 1; sex: female; lifeStage: adult; disposition: Mokpo National University; occurrenceID: FAAE845B-509A-5C84-AA6C-FDC08B84FC4C; **Taxon:** scientificName: *Hypenarivuligera*; **Location:** country: South Korea; stateProvince: GB; county: Gunwi; locality: Nakjeon-ri, Goro-myeon; **Identification:** identifiedBy: Sung-Soo Kim; dateIdentified: 2024; **Event:** samplingProtocol: Ultraviolet bucket trap; samplingEffort: 6 trapping hours; eventDate: 8/14/2011; **Record Level:** modified: 3/30/2025; language: en; collectionCode: Insects; basisOfRecord: PreservedSpecimen
- **Type status:** Other material. **Occurrence:** recordedBy: Choi, Sei-Woong; individualCount: 1; sex: female; lifeStage: adult; disposition: Mokpo National University; occurrenceID: 6FDA4F14-6D48-5379-9706-C7F23425A199; **Taxon:** scientificName: *Hypenarivuligera*; **Location:** country: South Korea; stateProvince: GN; county: Hadong; locality: Hwagae-myeon; verbatimElevation: 702; verbatimCoordinates: 35°18'21"N 127°38'11“E; **Identification:** identifiedBy: Sei-Woong Choi; dateIdentified: 2024; **Event:** samplingProtocol: Ultraviolet bucket trap; samplingEffort: 6 trapping hours; eventDate: 6/21/2014; **Record Level:** modified: 3/30/2025; language: en; collectionCode: Insects; basisOfRecord: PreservedSpecimen
- **Type status:** Other material. **Occurrence:** recordedBy: Choi, Sei-Woong; individualCount: 1; sex: female; lifeStage: adult; disposition: Mokpo National University; occurrenceID: C5FC5806-C3B2-524C-BBF9-F22B414D9D0E; **Taxon:** scientificName: *Hypenarivuligera*; **Location:** country: South Korea; stateProvince: GN; county: Sacheon; locality: Gonyang; **Identification:** identifiedBy: Sung-Soo Kim; dateIdentified: 2024; **Event:** samplingProtocol: Ultraviolet bucket trap; samplingEffort: 6 trapping hours; eventDate: 7/8/2023; **Record Level:** modified: 3/30/2025; language: en; collectionCode: Insects; basisOfRecord: PreservedSpecimen

##### Distribution

Korea, Japan.

##### Notes

Fig. 5d.

#### Hypena perspicua

Leech, 1900

F8FDFDF2-4217-587B-92FB-AA80ADB1F00D

##### Materials

- **Type status:** Other material. **Occurrence:** recordedBy: Choi, Sei-Woong; individualCount: 1; sex: male; lifeStage: adult; disposition: Mokpo National University; occurrenceID: 93B669B0-4F26-538B-8963-B111FD230DC6; **Taxon:** scientificName: *Hypenaperspicua*; **Location:** country: South Korea; stateProvince: JJ; county: Seogwipo; locality: Mt. Halla; verbatimCoordinates: 33°19'N 126°36'E; **Identification:** identifiedBy: Sei-Woong Choi; dateIdentified: 2024; **Event:** samplingProtocol: Ultraviolet bucket trap; samplingEffort: 6 trapping hours; eventDate: 8/24/2004; **Record Level:** modified: 3/30/2025; language: en; collectionCode: Insects; basisOfRecord: PreservedSpecimen
- **Type status:** Other material. **Occurrence:** recordedBy: Choi, Sei-Woong; individualCount: 1; sex: male; lifeStage: adult; disposition: Mokpo National University; occurrenceID: CDCF5057-7A39-52EF-9D00-C7C7F02050AB; **Taxon:** scientificName: *Hypenaperspicua*; **Location:** country: South Korea; stateProvince: JJ; county: Seogwipo; locality: Seoquipo Natural Recreation Forest; verbatimCoordinates: 33°18'39"N 126°27'32“E; **Identification:** identifiedBy: Sung-Soo Kim; dateIdentified: 2024; **Event:** samplingProtocol: Ultraviolet bucket trap; samplingEffort: 6 trapping hours; eventDate: 9/25/2019; **Record Level:** modified: 3/30/2025; language: en; collectionCode: Insects; basisOfRecord: PreservedSpecimen

##### Distribution

Korea, Japan, China, Taiwan, Thailand.

##### Notes

This is the first record for the Korean fauna. The DNA barcode of *Hypenaperspicua* was first registered in this study (GenBank accession No. PV274488). The p-distance with *H.proboscidalis* and *H.tamsi* was 9.99%, while the lowest genetic distance (5.07%) was recorded for *H.nigrobasalis* (Figs 5e, 8c, d).

##### Diagnosis

Wingspan 30 mm. Antenna filiform, brown; vertex yellowish-brown scales; frons with dark brown scales; labial palpi covered with obliquely erected dark brown scales on dorsal and ventral surfaces, second segment three times longer than the third segment, distal end of third segment yellowish. Thorax dark brown; tegula consisting of long scales and hair-like scales. Forewing ground colour pale brown; a large rhomboidal dark brown medial patch, inner line from the base to outer line bordered dorsally with yellowish-white, outer line strongly curved at 1/3 from costa where dark brown band from apex meets; subapical and subtornus dots blackish. Hindwing brown with a large blackish discal dot. Abdomen brown with dark brown erected scales at middle of 1^st^ to 4^th^ terga. **Male genitalia.** Uncus long, weakly tapering, apex strongly hooked, pointed. Tegumen hood-shaped; tuba analis long; juxta broad, thick diamond-shape on its side; saccus long, semi-rounded. Valva simple, weakly sclerotised, medially expanded; costal margin medially swollen, ventrally swollen; sacculus ventral margin medially weakly invaginated; basally a relatively long division of clavus, about a half of length of valva with a nipple-shaped process. Aedeagus long, rod-shaped, anteriorly simple; vesica tubular, cornuti a row of spines and a small sclerotised plate.

#### Hypena bicoloralis

Graeser, 1889

10D71318-2E4A-5F6B-949C-13CA8B788F8D

##### Materials

- **Type status:** Other material. **Occurrence:** recordedBy: Choi, Sei-Woong; individualCount: 1; sex: female; lifeStage: adult; disposition: Mokpo National University; occurrenceID: 007B04BF-575A-5B56-AE51-E0A221592352; **Taxon:** scientificName: *Hypenabicoloralis*; **Location:** country: South Korea; stateProvince: GW; county: Yangyang; locality: Seorim-ri, Seo-myeon; **Identification:** identifiedBy: Sung-Soo Kim; dateIdentified: 2024; **Event:** samplingProtocol: Ultraviolet bucket trap; samplingEffort: 6 trapping hours; eventDate: 5/17/2017; **Record Level:** modified: 3/30/2025; language: en; collectionCode: Insects; basisOfRecord: PreservedSpecimen
- **Type status:** Other material. **Occurrence:** recordedBy: Choi, Sei-Woong; individualCount: 1; sex: female; lifeStage: adult; disposition: Mokpo National University; occurrenceID: 52A3DC23-3C21-5453-990C-D6F509F815FE; **Taxon:** scientificName: *Hypenabicoloralis*; **Location:** country: South Korea; stateProvince: JB; county: Jeongeup; locality: Mt. Duseoung, Gobu-myeon; **Identification:** identifiedBy: Sung-Soo Kim; dateIdentified: 2024; **Event:** samplingProtocol: Ultraviolet bucket trap; samplingEffort: 6 trapping hours; eventDate: 7/7/2013; **Record Level:** modified: 3/30/2025; language: en; collectionCode: Insects; basisOfRecord: PreservedSpecimen
- **Type status:** Other material. **Occurrence:** recordedBy: Choi, Sei-Woong; individualCount: 1; sex: male; lifeStage: adult; disposition: Mokpo National University; occurrenceID: 594B6F35-B3CC-5FC0-AF8C-404AAE16610F; **Taxon:** scientificName: *Hypenabicoloralis*; **Location:** country: South Korea; stateProvince: JN; county: Goheung; locality: Bongnae-myeon; verbatimElevation: 86; verbatimCoordinates: 34°28'45"N 127°26'41“E; **Identification:** identifiedBy: Sei-Woong Choi; dateIdentified: 2024; **Event:** samplingProtocol: Ultraviolet bucket trap; samplingEffort: 6 trapping hours; eventDate: 4/23/2009; **Record Level:** modified: 3/30/2025; language: en; collectionCode: Insects; basisOfRecord: PreservedSpecimen
- **Type status:** Other material. **Occurrence:** recordedBy: Choi, Sei-Woong; individualCount: 1; sex: female; lifeStage: adult; disposition: Mokpo National University; occurrenceID: A1D73ACC-D2E0-57D7-9934-60D6188E3CDF; **Taxon:** scientificName: *Hypenabicoloralis*; **Location:** country: South Korea; stateProvince: JN; county: Haenam; locality: Sanho-ri, Hwawon-myeon; verbatimElevation: 60; verbatimCoordinates: 34°39'44"N 126°16'40“E; **Identification:** identifiedBy: Sei-Woong Choi; dateIdentified: 2024; **Event:** samplingProtocol: Ultraviolet bucket trap; samplingEffort: 6 trapping hours; eventDate: 8/14/2023; **Record Level:** modified: 3/30/2025; language: en; collectionCode: Insects; basisOfRecord: PreservedSpecimen
- **Type status:** Other material. **Occurrence:** recordedBy: Choi, Sei-Woong; individualCount: 1; sex: female; lifeStage: adult; disposition: Mokpo National University; occurrenceID: ECFBFAE9-F0D6-59BE-A197-7149736154DF; **Taxon:** scientificName: *Hypenabicoloralis*; **Location:** country: South Korea; stateProvince: JN; county: Muan; locality: Mt. Seungdal; **Identification:** identifiedBy: Sei-Woong Choi; dateIdentified: 2024; **Event:** samplingProtocol: Ultraviolet bucket trap; samplingEffort: 6 trapping hours; eventDate: 5/12/2003; **Record Level:** modified: 3/30/2025; language: en; collectionCode: Insects; basisOfRecord: PreservedSpecimen

##### Distribution

Korea, Japan, Russian Far East.

##### Notes

Fig. 5f.

#### Hypena mandarina

Leech, 1900

982F8121-1FBA-50B5-8872-FB9843B201D5

##### Materials

- **Type status:** Other material. **Occurrence:** recordedBy: Choi, Sei-Woong; individualCount: 1; sex: male; lifeStage: adult; disposition: Mokpo National University; occurrenceID: 11093478-E51F-5491-AEEB-794BF5134E59; **Taxon:** scientificName: *Hypenamandarina*; **Location:** country: South Korea; stateProvince: GN; county: Hamyang; locality: Mt. Jiri, Macheon-myeon; verbatimElevation: 760; verbatimCoordinates: 35°21'18"N 127°38'08“E; **Identification:** identifiedBy: Sei-Woong Choi; dateIdentified: 2024; **Event:** samplingProtocol: Ultraviolet bucket trap; samplingEffort: 6 trapping hours; eventDate: 8/13/2009; **Record Level:** modified: 3/30/2025; language: en; collectionCode: Insects; basisOfRecord: PreservedSpecimen
- **Type status:** Other material. **Occurrence:** recordedBy: Choi, Sei-Woong; individualCount: 1; sex: female; lifeStage: adult; disposition: Mokpo National University; occurrenceID: E41D8B6B-3BF6-56CA-BC9E-287CEF9C3F6E; **Taxon:** scientificName: *Hypenamandarina*; **Location:** country: South Korea; stateProvince: GN; county: Hamyang; locality: Mt. Jiri, Macheon-myeon; verbatimElevation: 760; verbatimCoordinates: 35°21'18"N 127°38'08“E; **Identification:** identifiedBy: Sei-Woong Choi; dateIdentified: 2024; **Event:** samplingProtocol: Ultraviolet bucket trap; samplingEffort: 6 trapping hours; eventDate: 8/13/2009; **Record Level:** modified: 3/30/2025; language: en; collectionCode: Insects; basisOfRecord: PreservedSpecimen
- **Type status:** Other material. **Occurrence:** recordedBy: Choi, Sei-Woong; individualCount: 1; sex: male; lifeStage: adult; disposition: Mokpo National University; occurrenceID: ED53C42B-25EF-5B05-BB0D-A909EAD9AFD9; **Taxon:** scientificName: *Hypenamandarina*; **Location:** country: South Korea; stateProvince: GN; county: Hadong; locality: Hwagae-myeon; verbatimElevation: 702; verbatimCoordinates: 35°18'21"N 127°38'11“E; **Identification:** identifiedBy: Sei-Woong Choi; dateIdentified: 2024; **Event:** samplingProtocol: Ultraviolet bucket trap; samplingEffort: 6 trapping hours; eventDate: 6/21/2009; **Record Level:** modified: 3/30/2025; language: en; collectionCode: Insects; basisOfRecord: PreservedSpecimen
- **Type status:** Other material. **Occurrence:** recordedBy: Choi, Sei-Woong; individualCount: 1; sex: male; lifeStage: adult; disposition: Mokpo National University; occurrenceID: 5F7D0FC7-2FA0-5A2A-8670-E44C06E838DB; **Taxon:** scientificName: *Hypenamandarina*; **Location:** country: South Korea; stateProvince: GN; county: Hamyang; locality: Mt. Jiri, Macheon-myeon; verbatimElevation: 760; verbatimCoordinates: 35°21'18"N 127°38'08“E; **Identification:** identifiedBy: Sei-Woong Choi; dateIdentified: 2024; **Event:** samplingProtocol: Ultraviolet bucket trap; samplingEffort: 6 trapping hours; eventDate: 6/26/2008; **Record Level:** modified: 3/30/2025; language: en; collectionCode: Insects; basisOfRecord: PreservedSpecimen
- **Type status:** Other material. **Occurrence:** recordedBy: Choi, Sei-Woong; individualCount: 1; sex: male; lifeStage: adult; disposition: Mokpo National University; occurrenceID: F965802B-BDC8-5B79-9617-6D424537CC95; **Taxon:** scientificName: *Hypenamandarina*; **Location:** country: South Korea; stateProvince: GN; county: Hadong; locality: Hwagae-myeon; verbatimElevation: 702; verbatimCoordinates: 35°18'21"N 127°38'11“E; **Identification:** identifiedBy: Sei-Woong Choi; dateIdentified: 2024; **Event:** samplingProtocol: Ultraviolet bucket trap; samplingEffort: 6 trapping hours; eventDate: 6/8/2019; **Record Level:** modified: 3/30/2025; language: en; collectionCode: Insects; basisOfRecord: PreservedSpecimen
- **Type status:** Other material. **Occurrence:** recordedBy: Choi, Sei-Woong; individualCount: 1; sex: male; lifeStage: adult; disposition: Mokpo National University; occurrenceID: 1729F710-688A-525F-8EAA-863DED8B10C4; **Taxon:** scientificName: *Hypenamandarina*; **Location:** country: South Korea; stateProvince: JN; county: Gurye; locality: Mt. Jiri, Sandong-myeon; verbatimElevation: 923; verbatimCoordinates: 35°19'22"N 127°31'23“E; **Identification:** identifiedBy: Sei-Woong Choi; dateIdentified: 2024; **Event:** samplingProtocol: Ultraviolet bucket trap; samplingEffort: 6 trapping hours; eventDate: 8/15/2017; **Record Level:** modified: 3/30/2025; language: en; collectionCode: Insects; basisOfRecord: PreservedSpecimen
- **Type status:** Other material. **Occurrence:** recordedBy: Choi, Sei-Woong; individualCount: 1; sex: female; lifeStage: adult; disposition: Mokpo National University; occurrenceID: 3A443B10-C43F-5E5E-9B71-C515167A0825; **Taxon:** scientificName: *Hypenamandarina*; **Location:** country: South Korea; stateProvince: JN; county: Jangseong; locality: Baegyang-ro, Bukha-myeon; verbatimElevation: 663; verbatimCoordinates: 35°27'12"N 126°52'51“E; **Identification:** identifiedBy: Sei-Woong Choi; dateIdentified: 2024; **Event:** samplingProtocol: Ultraviolet bucket trap; samplingEffort: 6 trapping hours; eventDate: 6/16/2021; **Record Level:** modified: 3/30/2025; language: en; collectionCode: Insects; basisOfRecord: PreservedSpecimen
- **Type status:** Other material. **Occurrence:** recordedBy: Choi, Sei-Woong; individualCount: 1; sex: female; lifeStage: adult; disposition: Mokpo National University; occurrenceID: 43DB4434-2AA0-5F1D-86FB-3ED901336B92; **Taxon:** scientificName: *Hypenamandarina*; **Location:** country: South Korea; stateProvince: JJ; county: Seogwipo; locality: Mt. Halla, Hawon-dong; verbatimElevation: 997; verbatimCoordinates: 33°20'05"N 126°28'12“E; **Identification:** identifiedBy: Sei-Woong Choi; dateIdentified: 2024; **Event:** samplingProtocol: Ultraviolet bucket trap; samplingEffort: 6 trapping hours; eventDate: 8/26/2007; **Record Level:** modified: 3/30/2025; language: en; collectionCode: Insects; basisOfRecord: PreservedSpecimen

##### Distribution

Korea, China.

##### Notes

This is the first record for the Korean fauna. The DNA barcode of *Hypenamandarina* was first registered in this study (GenBank accession No. PV274489, PV274490). The p-distance with *H.proboscidalis* and *H.tamsi* was 9.79%, while the lowest genetic distance (5.71%) was recorded for *H.stygiana* (Figs 6a, 8e, f).

##### Diagnosis

Wingspan 29 mm. Antenna filiform, yellowish-brown; vertex and frons with erected yellowish-brown scales; labial palpi covered with obliquely erected dark brown scales on dorsal and ventral surfaces, second segment two times longer than the third segment. Thorax yellowish-brown; tegula consisting of long scales and hair-like scales. Forewing ground colour yellowish-brown; a large rhomboidal dark brown medial patch, inner line blackish, bordered with whitish, outer line projected at 1/3 from costa; subterminal line whitish, undulating; apical streak dark brown, bank-shaped. Hindwing yellowish-brown; a large dark brown discal dot. Abdomen greyish-brown with dark brown erected scales on the middle of 1^st^ to 4^th^ terga. **Male genitalia.** Uncus relatively short, hooked, apex pointed. Tegumen hood-shaped; tuba analis basally strongly sclerotised; juxta narrow with both sides extended and heads facing up; saccus long, semi-rounded. Valva simple, weakly sclerotised, medially expanded; costal margin medially swollen, ventral margin thin, sclerotised, largely undulating; sacculus with a small, sharply-pointed dorsal arm, ventral margin medially weakly invaginated; basally a relatively thin division of clavus. Aedeagus long, rod-shaped, anteriorly with dense spicules; vesica large, tubular, cornuti two patches of spines and a large sclerotised spine.

#### Hypena melanica

(Sugi, 1959)

021CC28A-B280-5307-B7D4-13A3E39F8504

##### Materials

- **Type status:** Other material. **Occurrence:** recordedBy: Choi, Sei-Woong; individualCount: 3; sex: males; lifeStage: adult; disposition: Mokpo National University; occurrenceID: C5607882-C817-5550-AED0-9AC099CA2024; **Taxon:** scientificName: *Hypenamelanica*; **Location:** country: South Korea; stateProvince: GW; county: Hoengseong; locality: Anheung-ri, Anheung-myeon; verbatimElevation: 510; **Identification:** identifiedBy: Sung-Soo Kim; dateIdentified: 2024; **Event:** samplingProtocol: Ultraviolet bucket trap; samplingEffort: 6 trapping hours; eventDate: 8/3/2016; **Record Level:** modified: 3/30/2025; language: en; collectionCode: Insects; basisOfRecord: PreservedSpecimen
- **Type status:** Other material. **Occurrence:** recordedBy: Choi, Sei-Woong; individualCount: 1; sex: female; lifeStage: adult; disposition: Mokpo National University; occurrenceID: 19BBAD01-749B-5A9A-8181-501F9755F43C; **Taxon:** scientificName: *Hypenamelanica*; **Location:** country: South Korea; stateProvince: GW; county: Hoengseong; locality: Anheung-ri, Anheung-myeon; verbatimElevation: 510; **Identification:** identifiedBy: Sung-Soo Kim; dateIdentified: 2024; **Event:** samplingProtocol: Ultraviolet bucket trap; samplingEffort: 6 trapping hours; eventDate: 8/3/2016; **Record Level:** modified: 3/30/2025; language: en; collectionCode: Insects; basisOfRecord: PreservedSpecimen
- **Type status:** Other material. **Occurrence:** recordedBy: Choi, Sei-Woong; individualCount: 1; sex: male; lifeStage: adult; disposition: Mokpo National University; occurrenceID: 4EF1315A-B4DE-5798-89BC-8330563A9E7C; **Taxon:** scientificName: *Hypenamelanica*; **Location:** country: South Korea; stateProvince: GW; county: Chuncheon; locality: Jiam-ri, Sabuk-myeon; **Identification:** identifiedBy: Sung-Soo Kim; dateIdentified: 2024; **Event:** samplingProtocol: Ultraviolet bucket trap; samplingEffort: 6 trapping hours; eventDate: 6/14/2014; **Record Level:** modified: 3/30/2025; language: en; collectionCode: Insects; basisOfRecord: PreservedSpecimen
- **Type status:** Other material. **Occurrence:** recordedBy: Choi, Sei-Woong; individualCount: 1; sex: female; lifeStage: adult; disposition: Mokpo National University; occurrenceID: 4DBC88A9-F29F-5AB6-AFD5-DD2DD4ED4F8A; **Taxon:** scientificName: *Hypenamelanica*; **Location:** country: South Korea; stateProvince: CB; county: Danyang; locality: Mt. Soback; verbatimElevation: 530; verbatimCoordinates: 36°59'N 128°28'E; **Identification:** identifiedBy: Sei-Woong Choi; dateIdentified: 2024; **Event:** samplingProtocol: Ultraviolet bucket trap; samplingEffort: 6 trapping hours; eventDate: 7/21/2005; **Record Level:** modified: 3/30/2025; language: en; collectionCode: Insects; basisOfRecord: PreservedSpecimen
- **Type status:** Other material. **Occurrence:** recordedBy: Choi, Sei-Woong; individualCount: 1; sex: male; lifeStage: adult; disposition: Mokpo National University; occurrenceID: A1BD5A1A-F769-5985-B19D-E035CF44F65E; **Taxon:** scientificName: *Hypenamelanica*; **Location:** country: South Korea; stateProvince: GN; county: Namhae; locality: Murim-ri, Idong-myeon; **Identification:** identifiedBy: Sung-Soo Kim; dateIdentified: 2024; **Event:** samplingProtocol: Ultraviolet bucket trap; samplingEffort: 6 trapping hours; eventDate: 6/10/2013; **Record Level:** modified: 3/30/2025; language: en; collectionCode: Insects; basisOfRecord: PreservedSpecimen
- **Type status:** Other material. **Occurrence:** recordedBy: Choi, Sei-Woong; individualCount: 1; sex: male; lifeStage: adult; disposition: Mokpo National University; occurrenceID: 0D7AAC6B-F89B-5D89-9331-B515512CD789; **Taxon:** scientificName: *Hypenamelanica*; **Location:** country: South Korea; stateProvince: GN; county: Namhae; locality: Murim-ri, Idong-myeon; **Identification:** identifiedBy: Sung-Soo Kim; dateIdentified: 2024; **Event:** samplingProtocol: Ultraviolet bucket trap; samplingEffort: 6 trapping hours; eventDate: 7/8/2013; **Record Level:** modified: 3/30/2025; language: en; collectionCode: Insects; basisOfRecord: PreservedSpecimen

##### Distribution

Korea, Japan.

##### Notes

Fig. 6b.

## Analysis

### Phylogeny of Hypena

**Figure 11.**
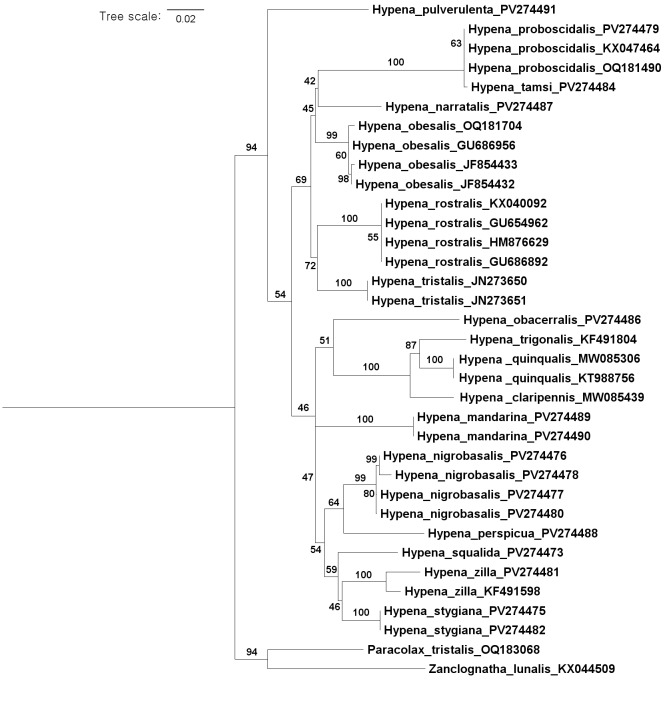
A phylogenetic analysis was conducted on 15 species of the genus *Hypena*, along with two species, *Zanclognathalunalis* and *Paracolaxtristalis* as the outgroup. The phylogenetic tree was constructed using the Maximum Likelihood (ML) method in IQ-tree 1.6.2, with the GTR+F+I+G4 model chosen, based on the Bayesian Information Criterion (BIC).

A phylogenetic analysis was performed on 15 species of the genus *Hypena* from the Palearctic region, including 11 species found in Korea (Fig. 11). Two species from the sister subfamily Herminiinae, *Zanclognathalunalis* and *Paracolaxtristalis*, were used as the outgroup. The results indicated that *Hypena* forms a monophyletic taxon. The ingroup was divided into three species groups; however, the supporting value was low (54%), suggesting a single species group instead.

## Supplementary Material

XML Treatment for Hypena

XML Treatment for Hypena sagitta

XML Treatment for Hypena claripennis

XML Treatment for Hypena amica

XML Treatment for Hypena trigonalis

XML Treatment for Hypena proboscidalis

XML Treatment for Hypena tamsi

XML Treatment for Hypena strigatus

XML Treatment for Hypena furva

XML Treatment for Hypena conspersalis

XML Treatment for Hypena sinuosa

XML Treatment for Hypena occata

XML Treatment for Hypena indicatalis

XML Treatment for Hypena subcyanea

XML Treatment for Hypena obacerralis

XML Treatment for Hypena tristalis

XML Treatment for Hypena narratalis

XML Treatment for Hypena pulverulenta

XML Treatment for Hypena abducalis

XML Treatment for Hypena kengkalis

XML Treatment for Hypena albopunctalis

XML Treatment for Hypena stygiana

XML Treatment for Hypena squalida

XML Treatment for Hypena nigrobasalis

XML Treatment for Hypena zilla

XML Treatment for Hypena rivuligera

XML Treatment for Hypena perspicua

XML Treatment for Hypena bicoloralis

XML Treatment for Hypena mandarina

XML Treatment for Hypena melanica

46D1257D-8BDE-522A-89AC-4F2EFE9863EC 10.3897/BDJ.13.e155581.suppl1 Supplementary material 1 List of the species included in our analyses and information about the sources of materials Data type genomic Brief description List of the species included in our analyses and information about the sources of materials, including collection locality, voucher and accession number in GenBank. File: oo_1290147.docx https://binary.pensoft.net/file/1290147 Dahee Jin, Sung-Soo Kim, Bora Shin, Sei-Woong Choi

## Figures and Tables

**Figure 1a.**
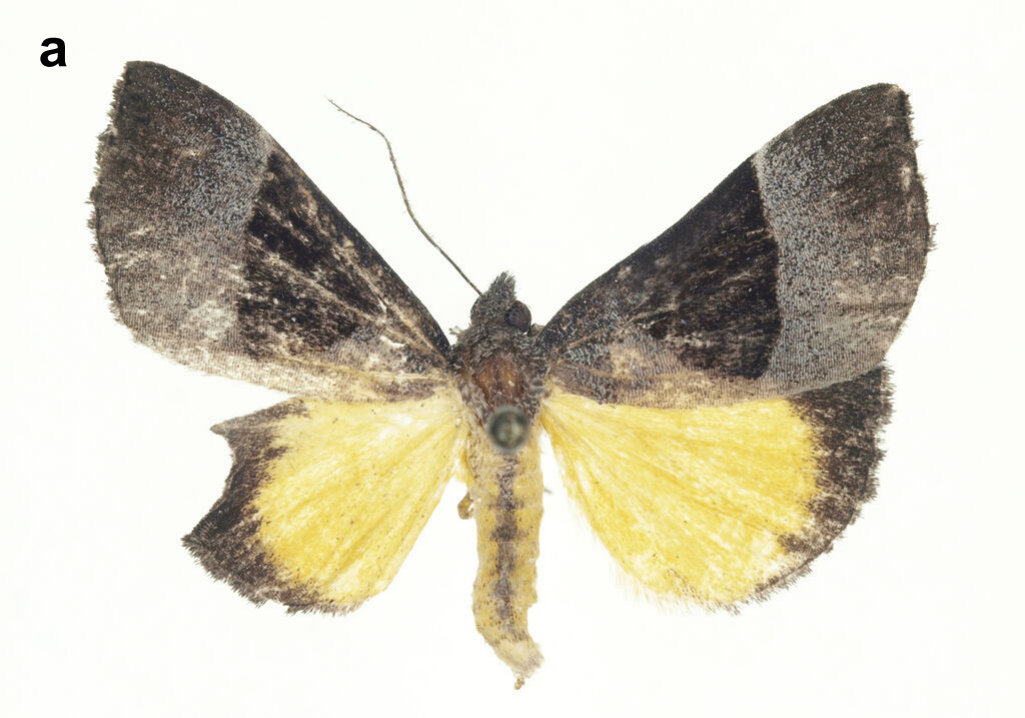
*Hypenasagitta*, male;

**Figure 1b.**
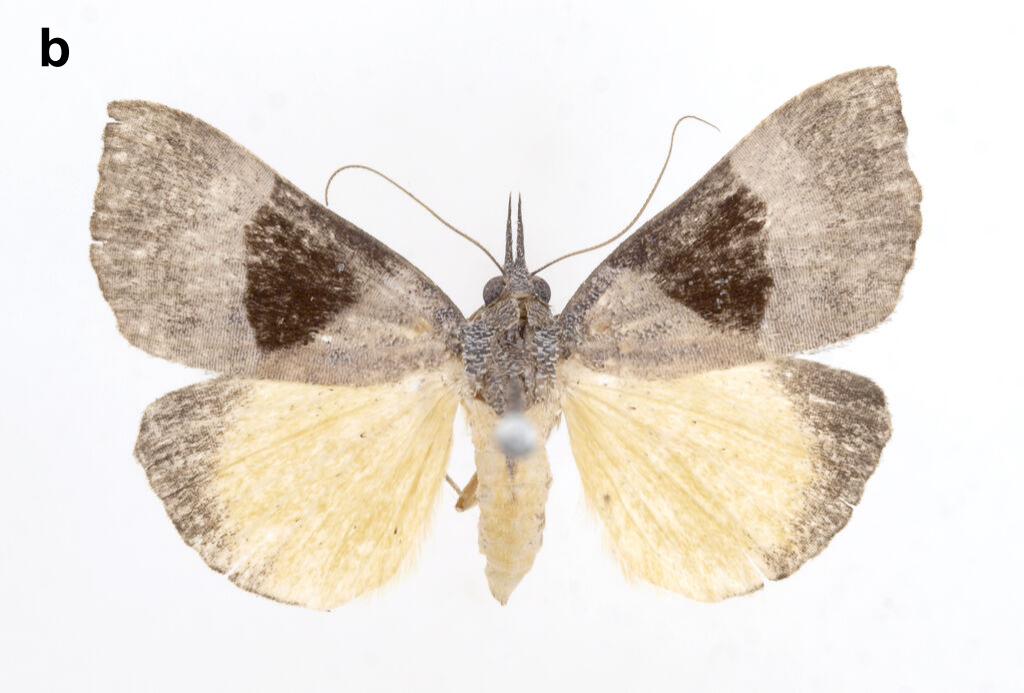
*Hypenasagitta*, female;

**Figure 1c.**
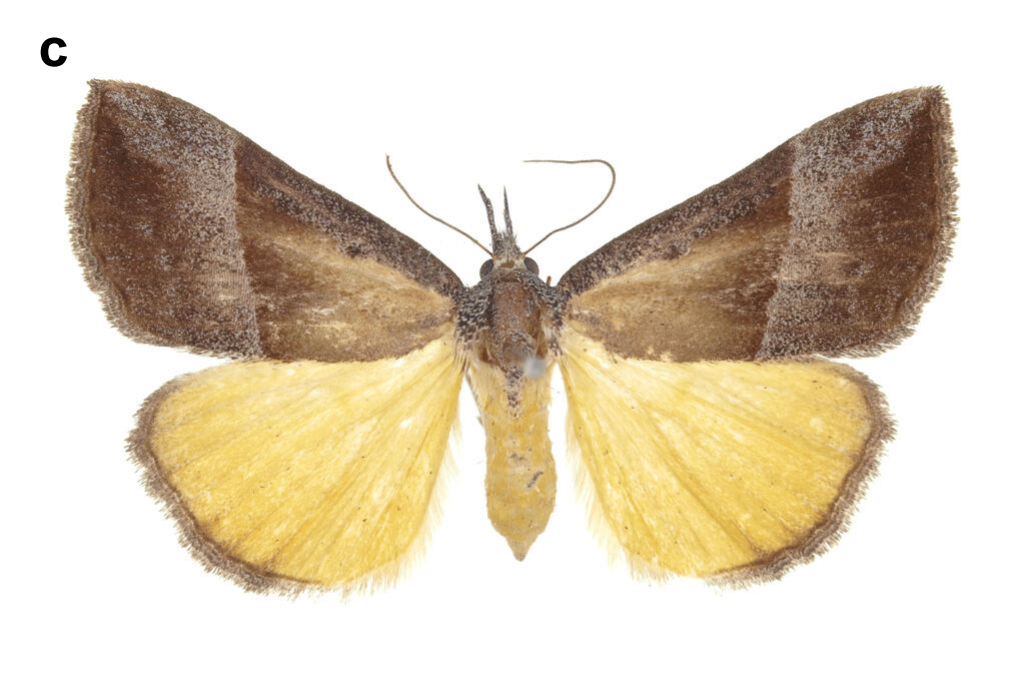
*Hypenaclaripennis*, male;

**Figure 1d.**
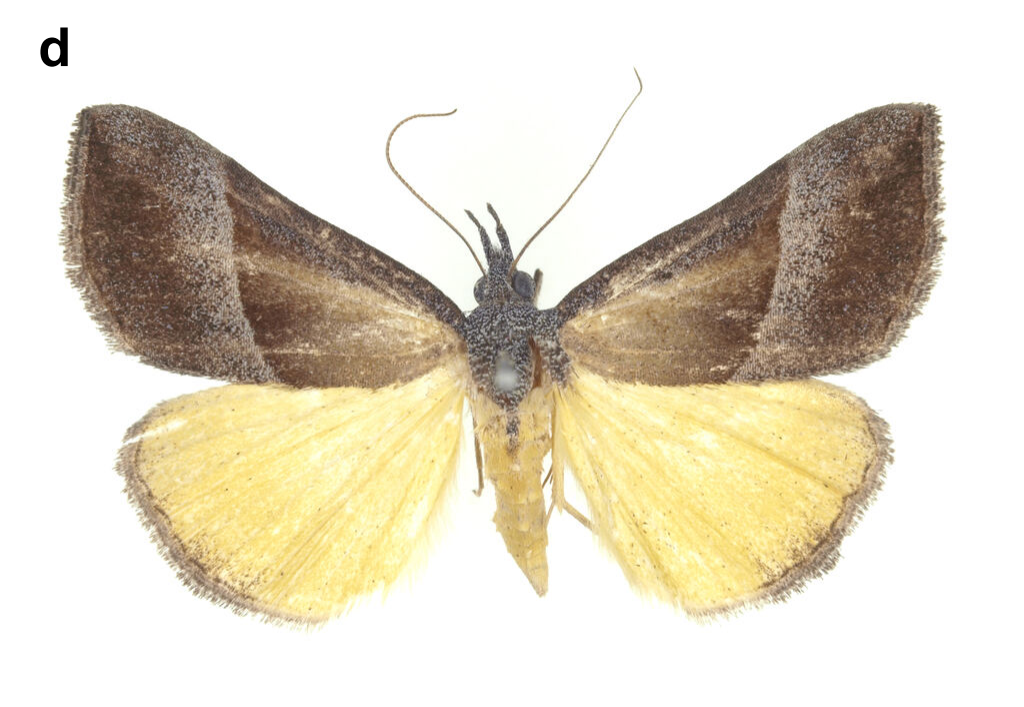
*Hypenaclaripennis*, female;

**Figure 1e.**
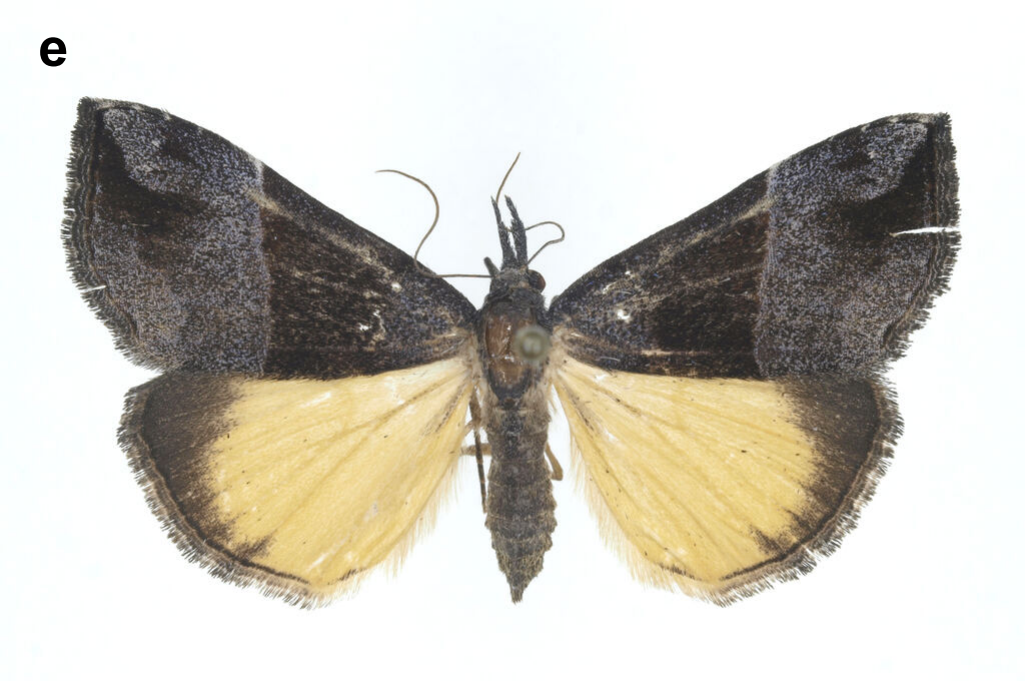
*Hypenaamica*, female;

**Figure 1f.**
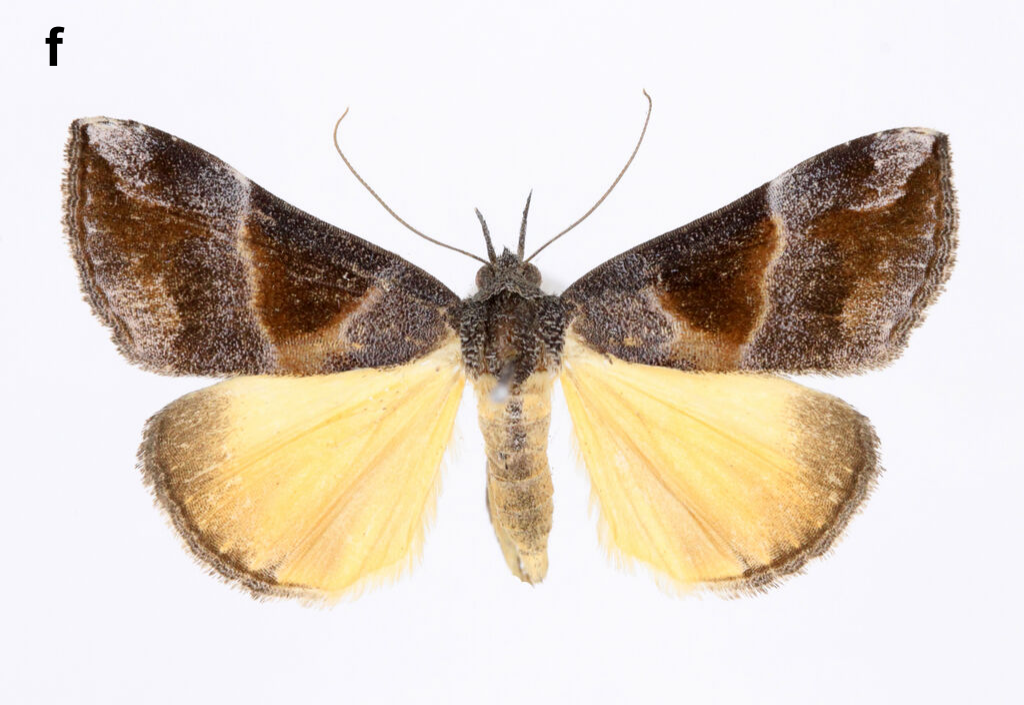
*Hypenatrigonalis*, male.

**Figure 2a.**
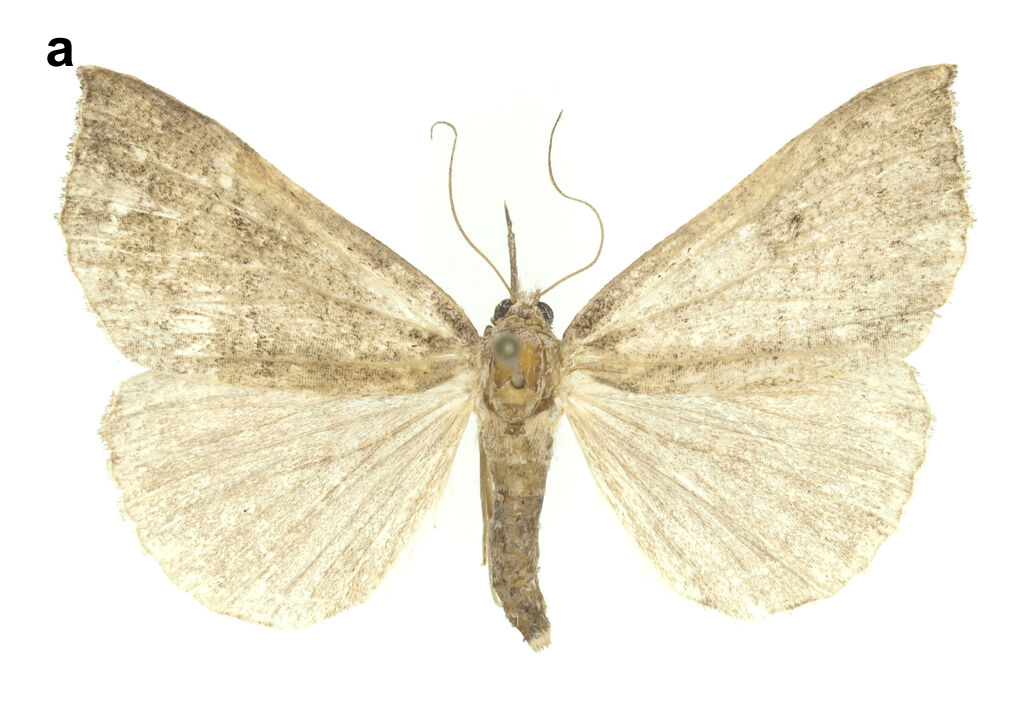
*Hypenaproboscidalis*, female;

**Figure 2b.**
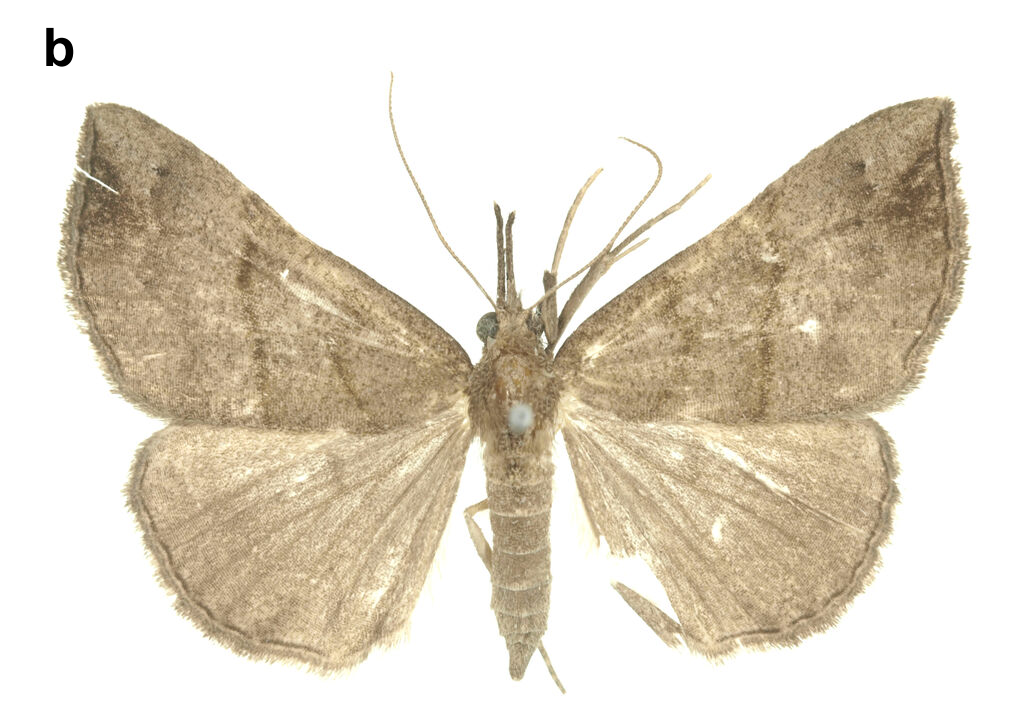
*Hypenatamsi*, male;

**Figure 2c.**
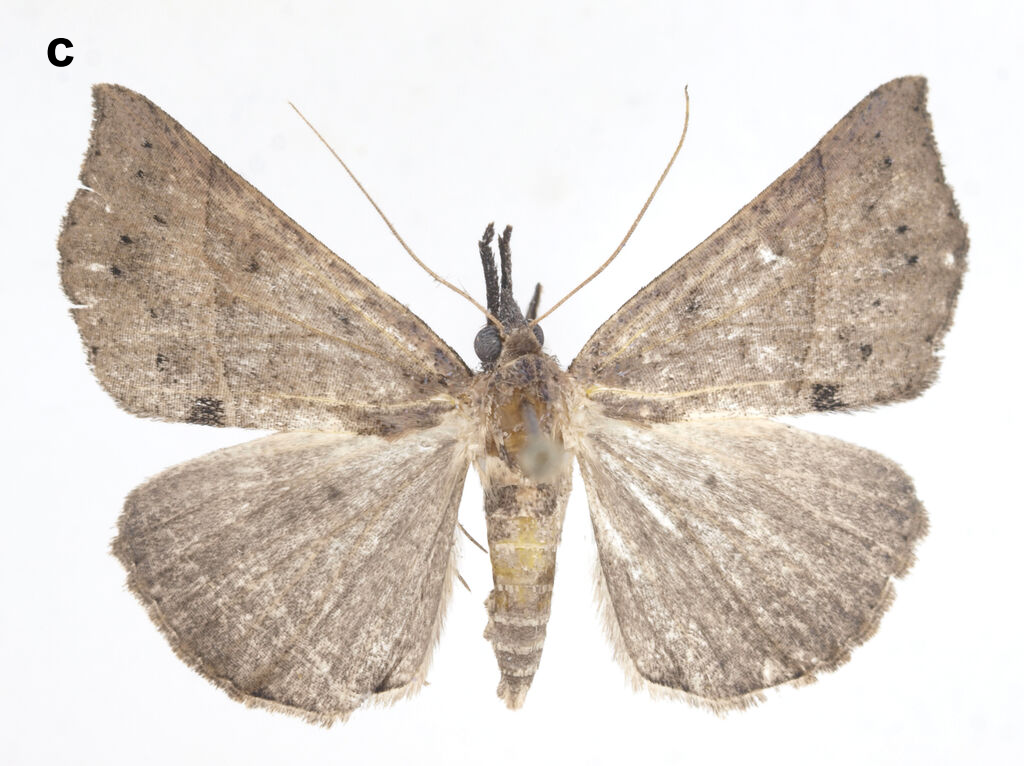
*Hypenastrigulatus*, male;

**Figure 2d.**
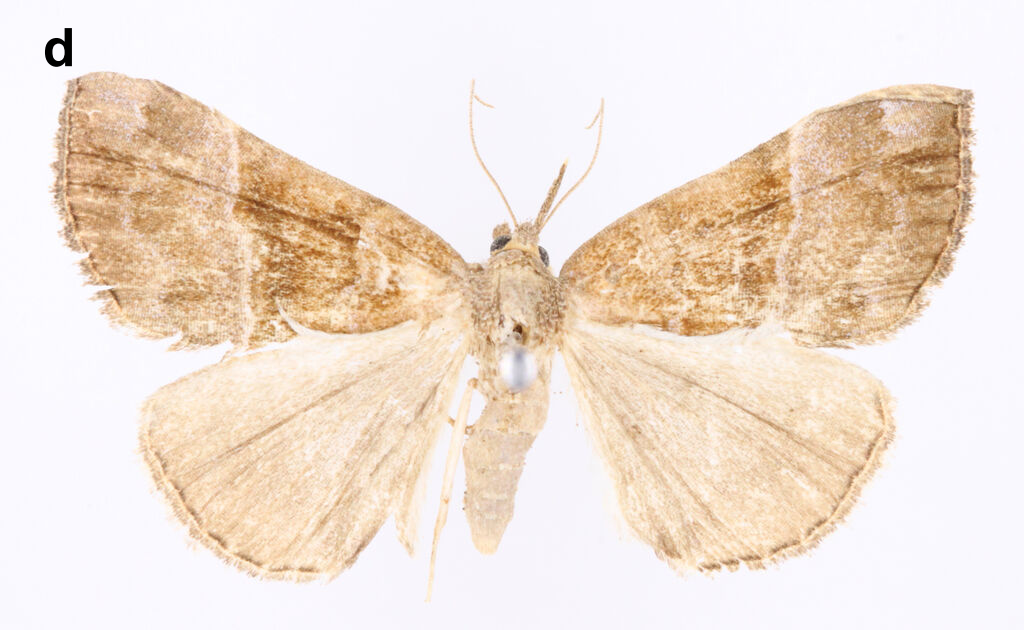
*Hypenaconspersalis*, male;

**Figure 2e.**
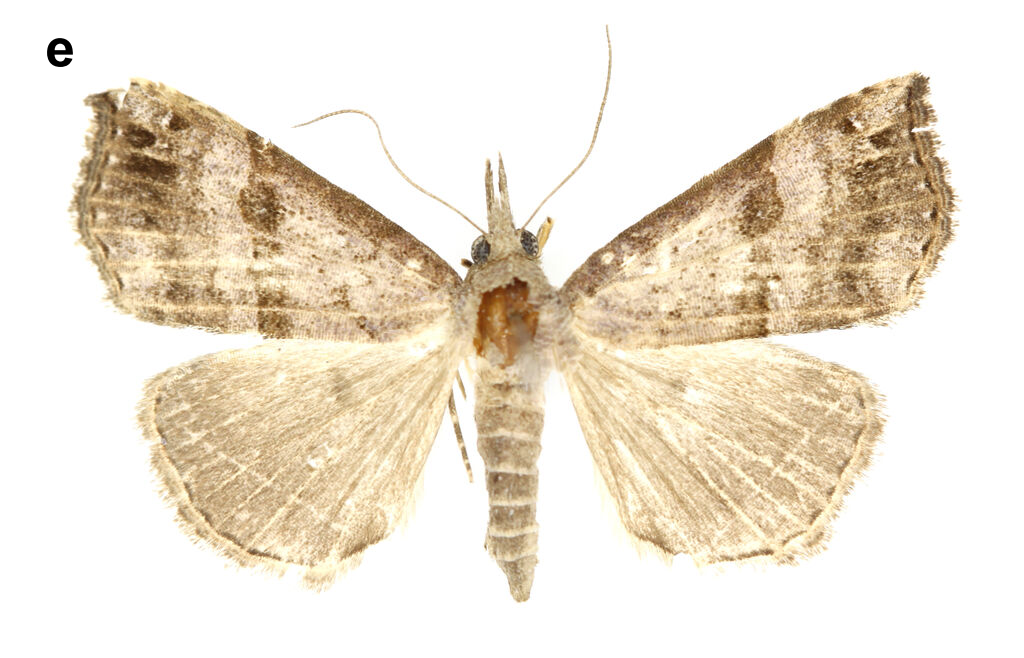
*Hypenasinuosa*, female;

**Figure 2f.**
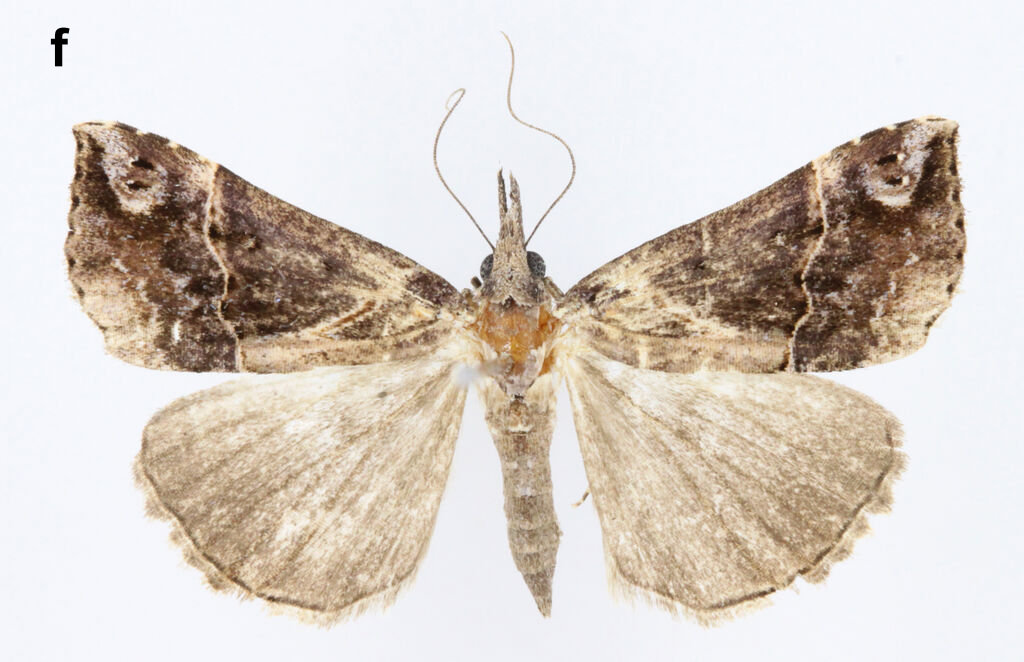
*Hypenaoccata*, male.

**Figure 3a.**
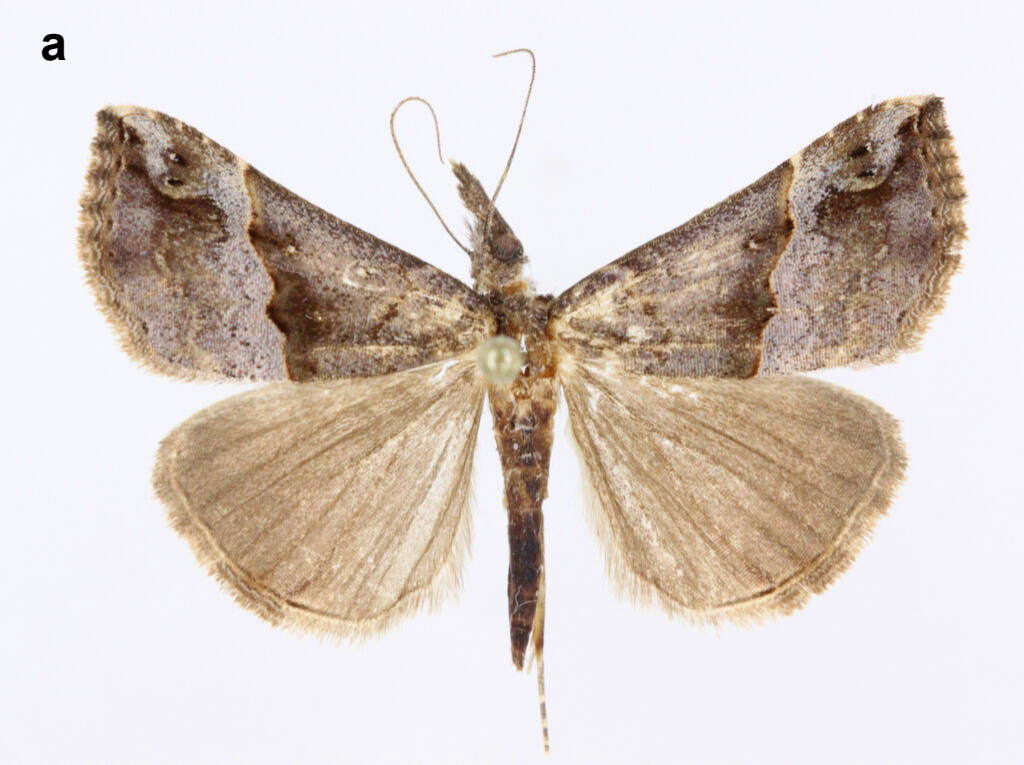
*Hypenaoccata*, female;

**Figure 3b.**
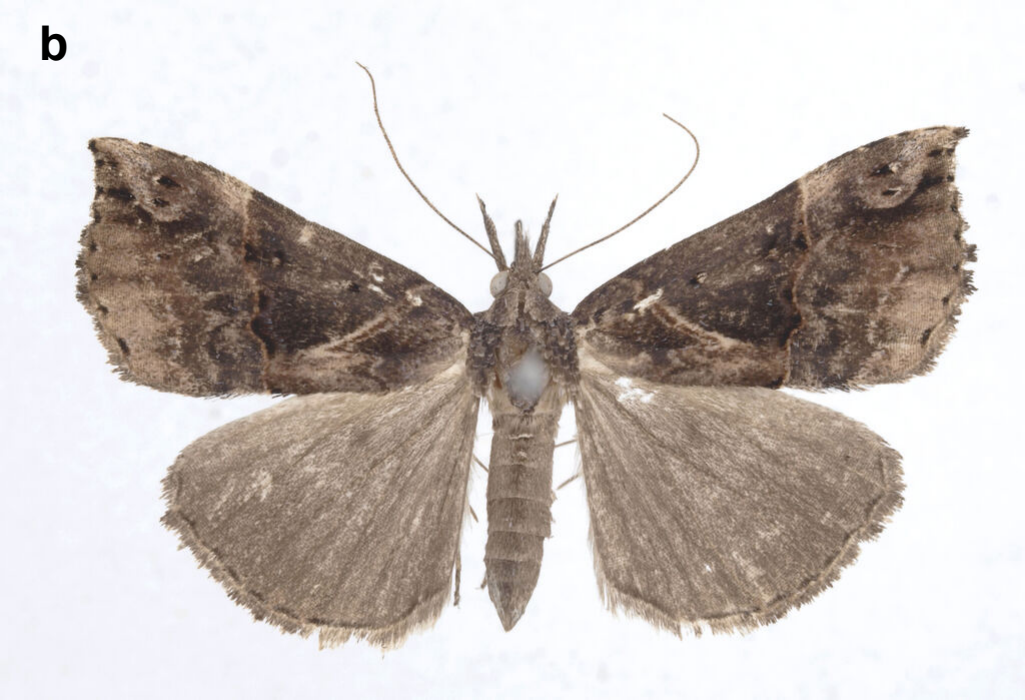
*Hypenaindicatalis*, male;

**Figure 3c.**
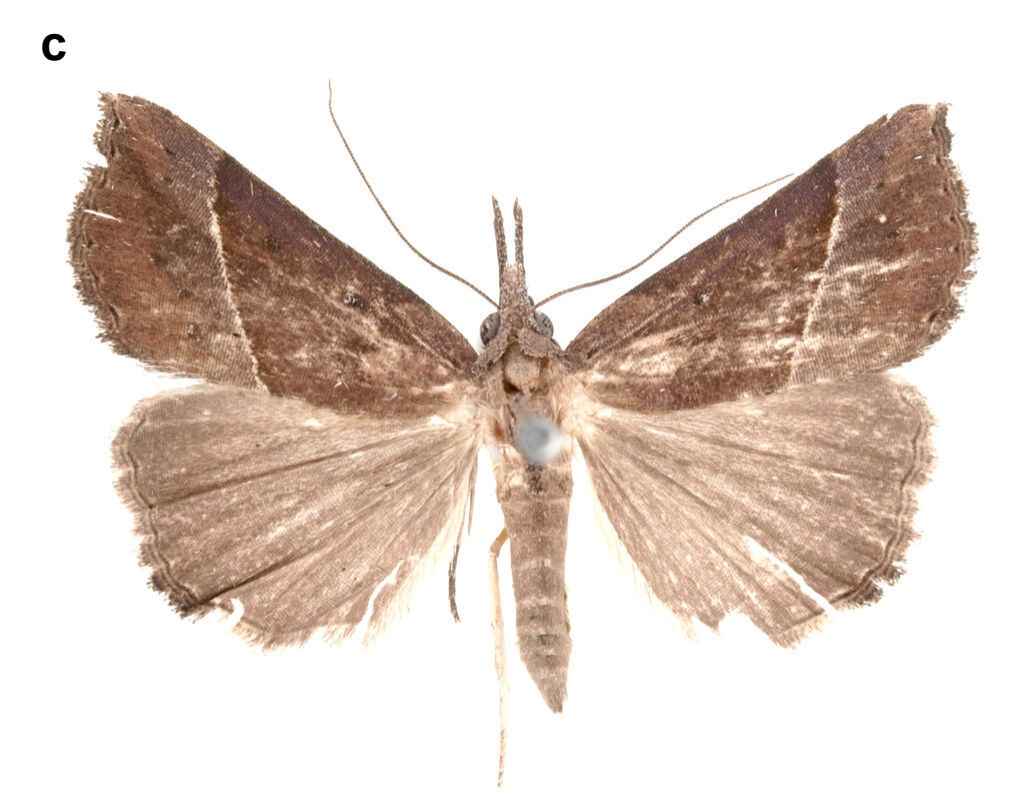
*Hypenasubcyanea*, male;

**Figure 3d.**
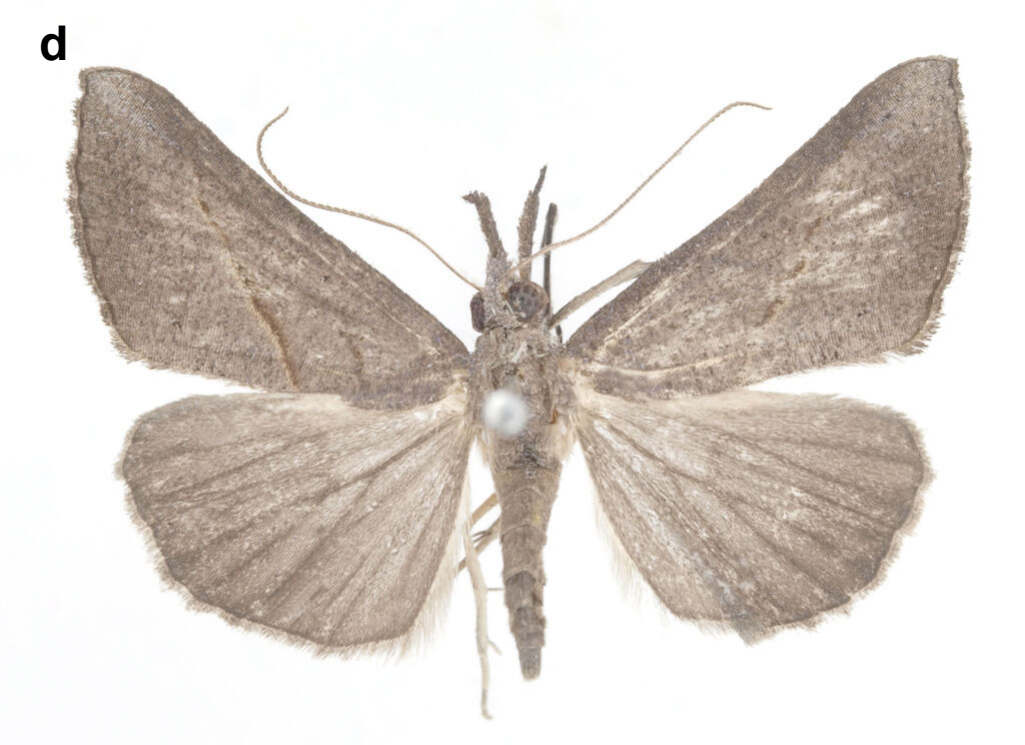
*Hypenaobacerralis*, male;

**Figure 3e.**
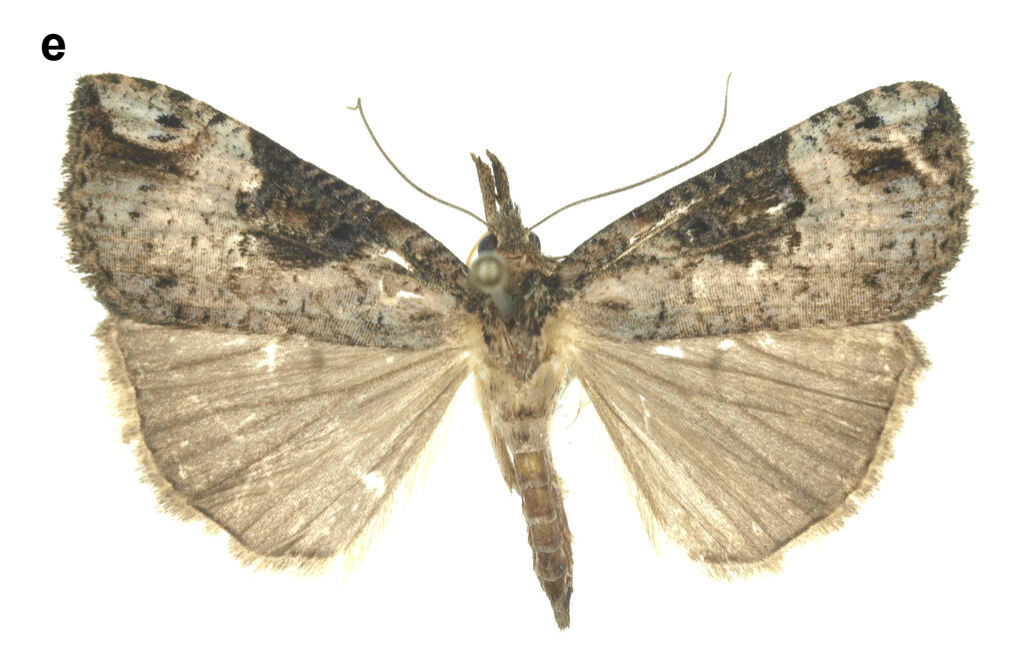
*Hypenatristalis*, female;

**Figure 3f.**
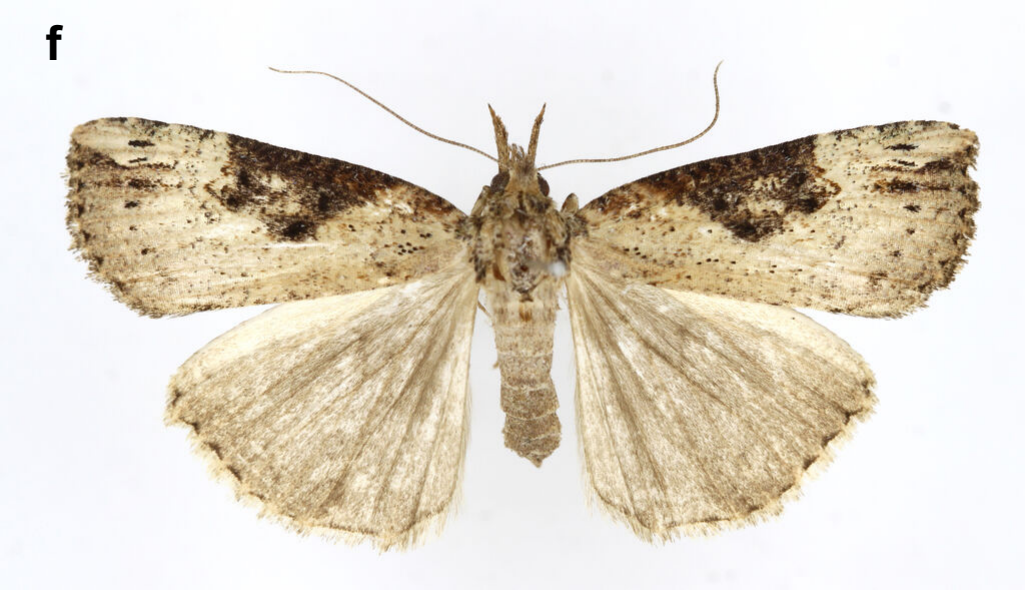
*Hypenanarratalis*, female.

**Figure 4a.**
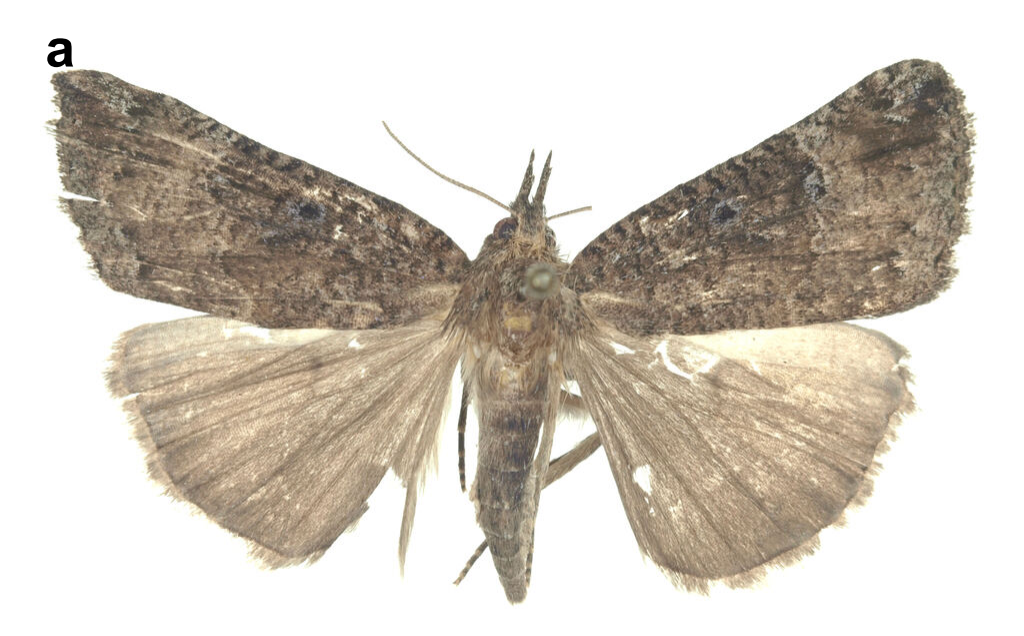
*Hypenanarratalis*, male;

**Figure 4b.**
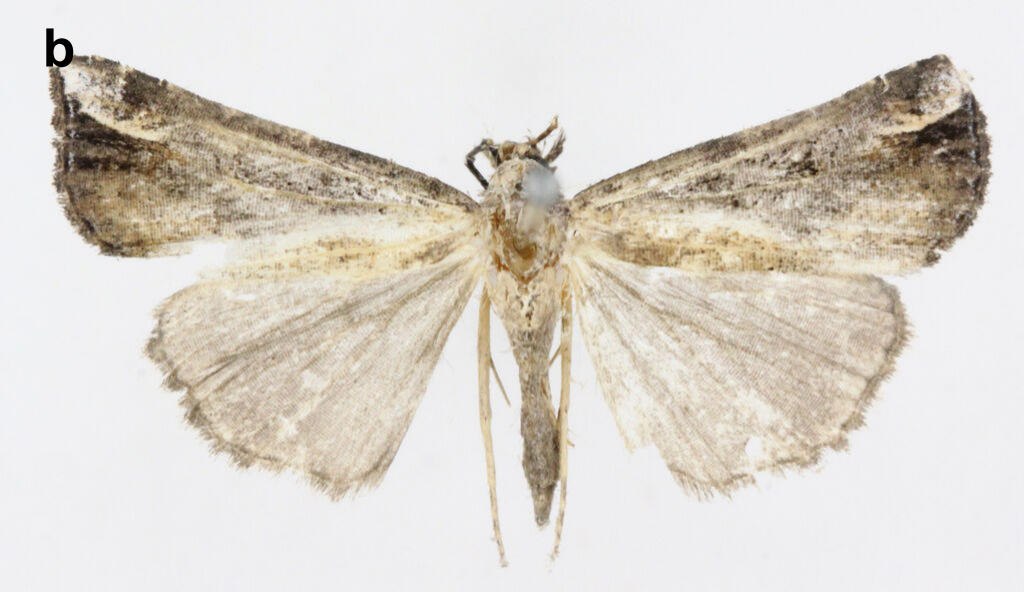
*Hypenapulverulenta*, male;

**Figure 4c.**
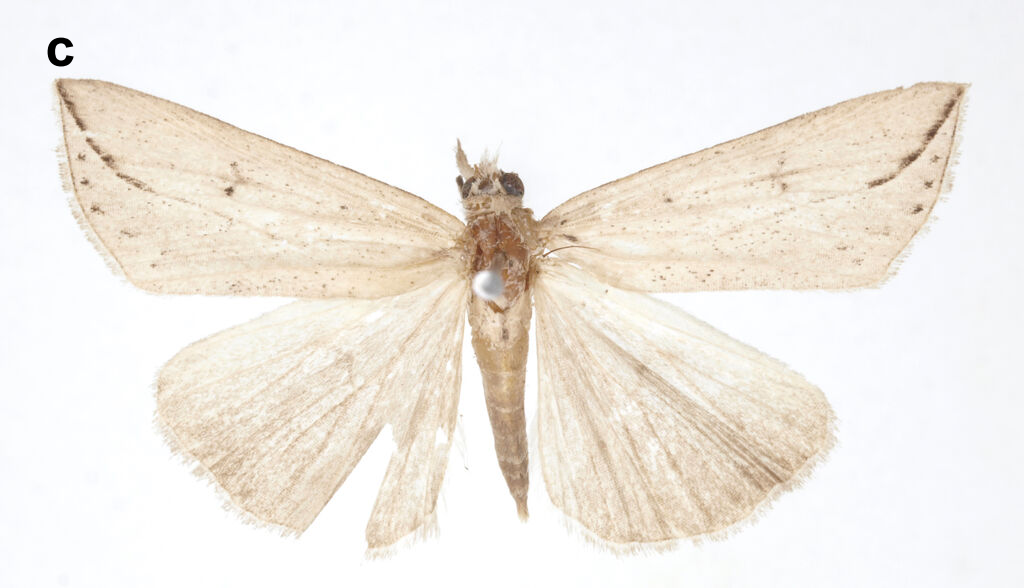
*Hypenaabducalis*, male;

**Figure 4d.**
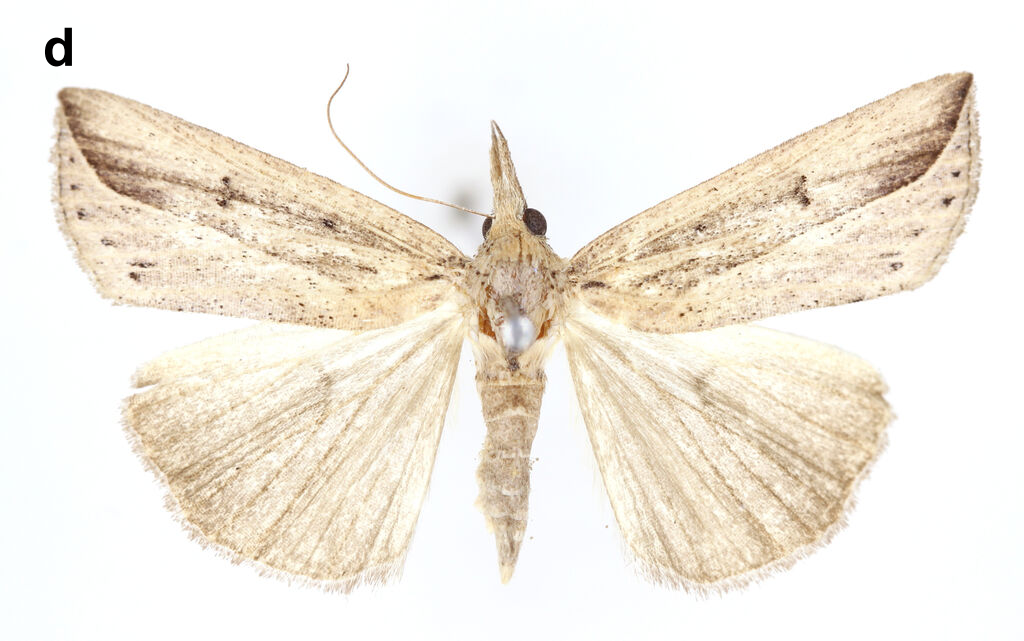
*Hypenaabducalis*, female;

**Figure 4e.**
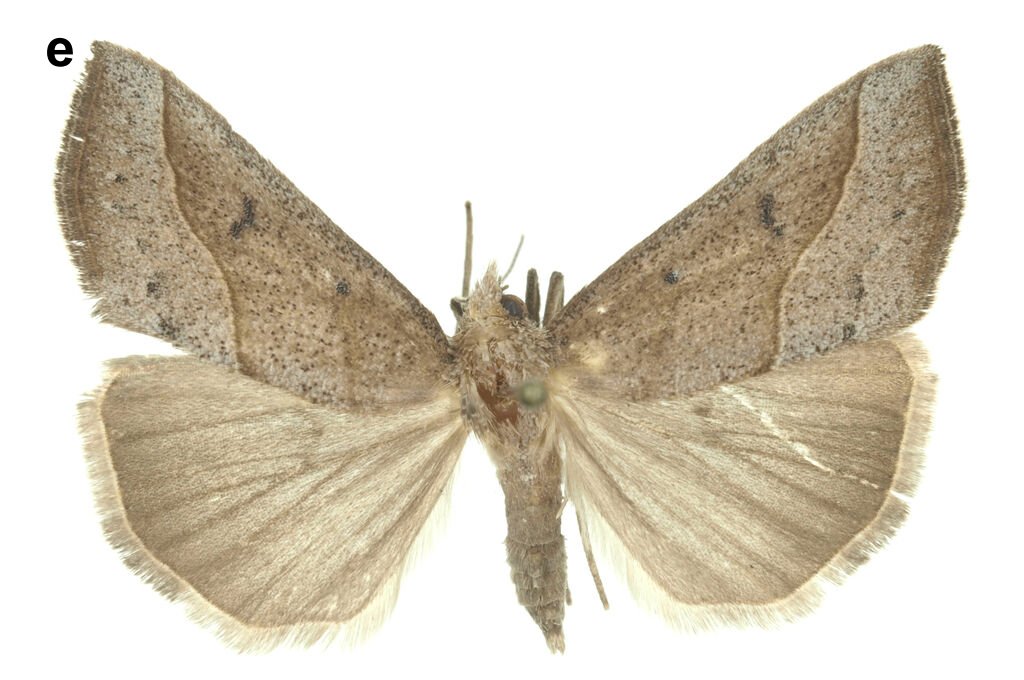
*Hypenakengkalis*, male;

**Figure 4f.**
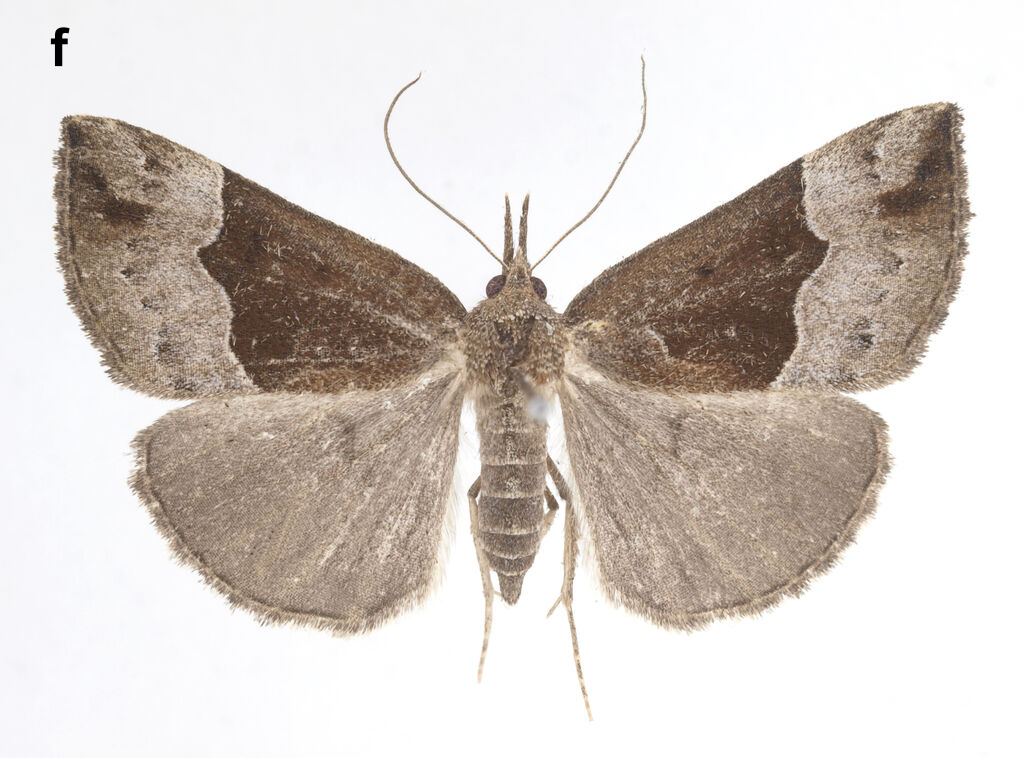
*Hypenastygiana*, female.

**Figure 5a.**
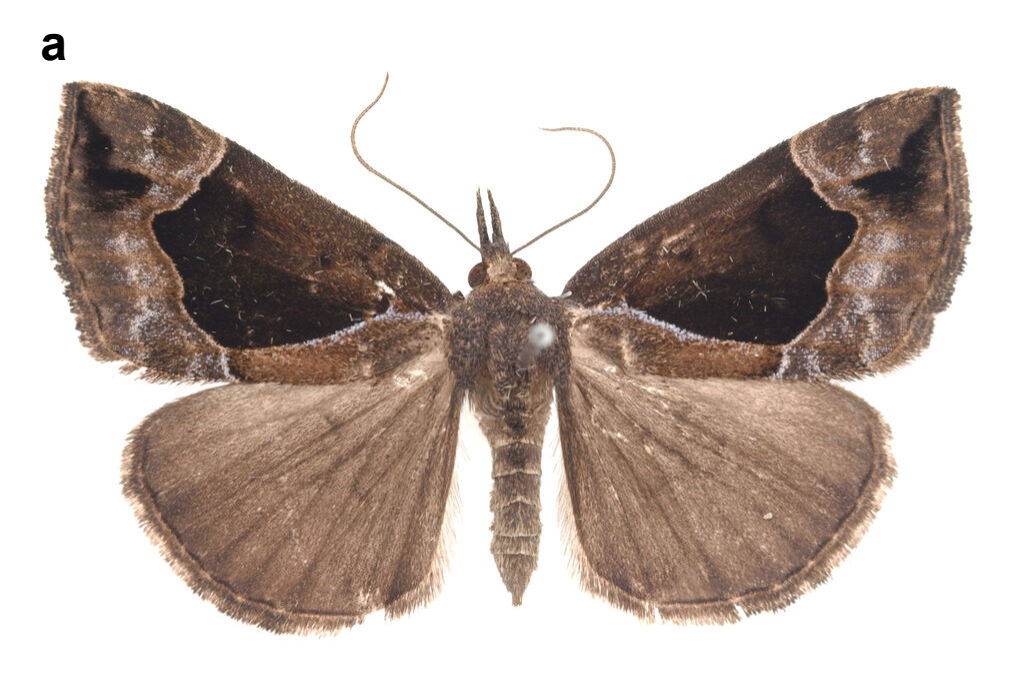
*Hypenasqualida*, female;

**Figure 5b.**
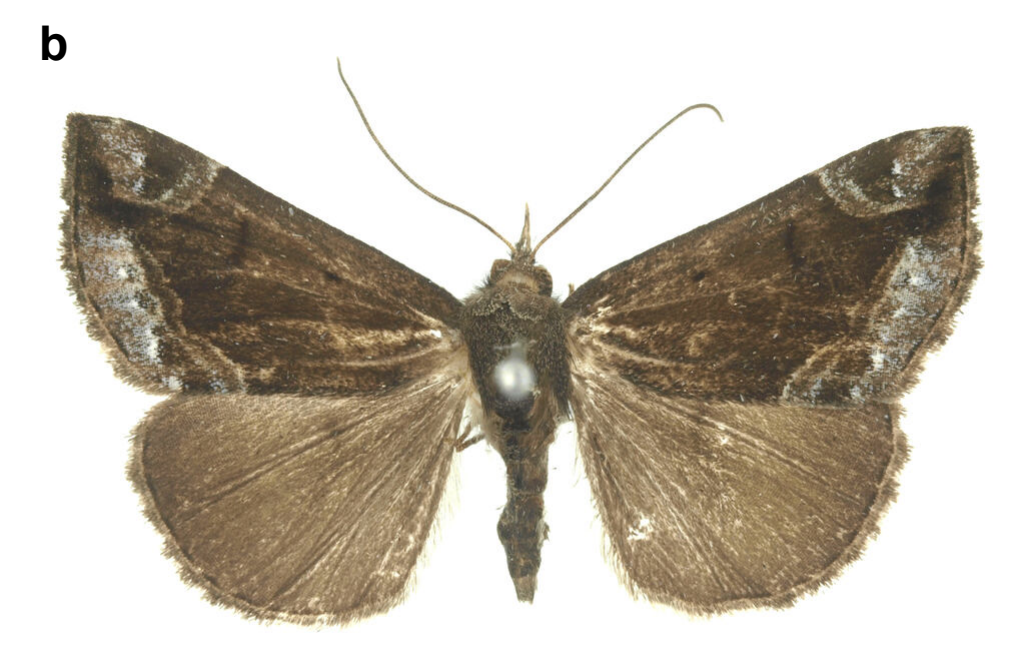
*Hypenanigrobasalis*, male;

**Figure 5c.**
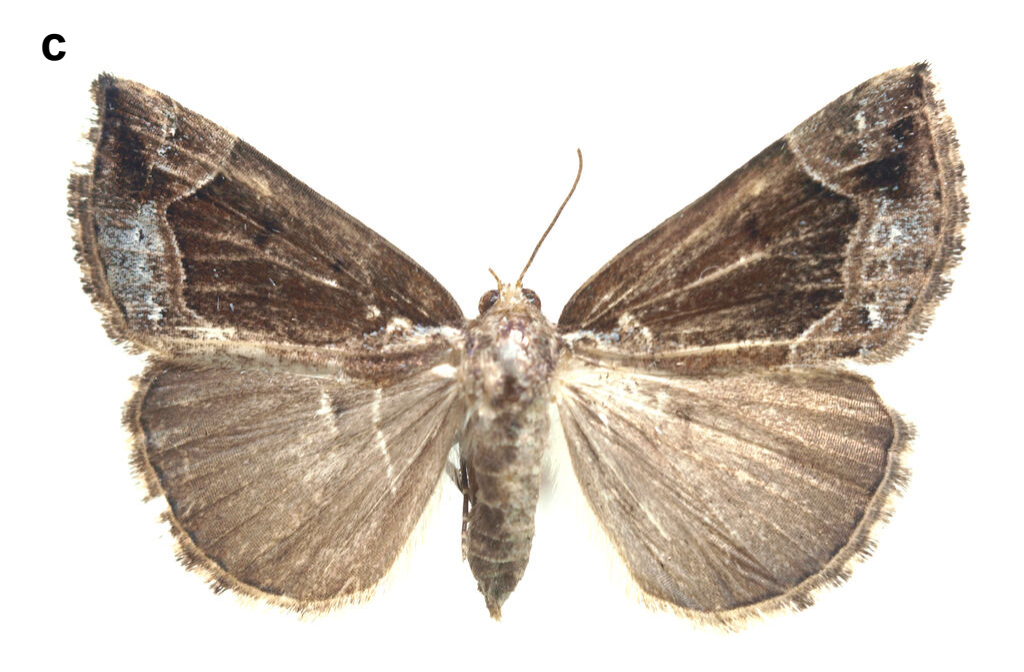
*Hypenazilla*, female;

**Figure 5d.**
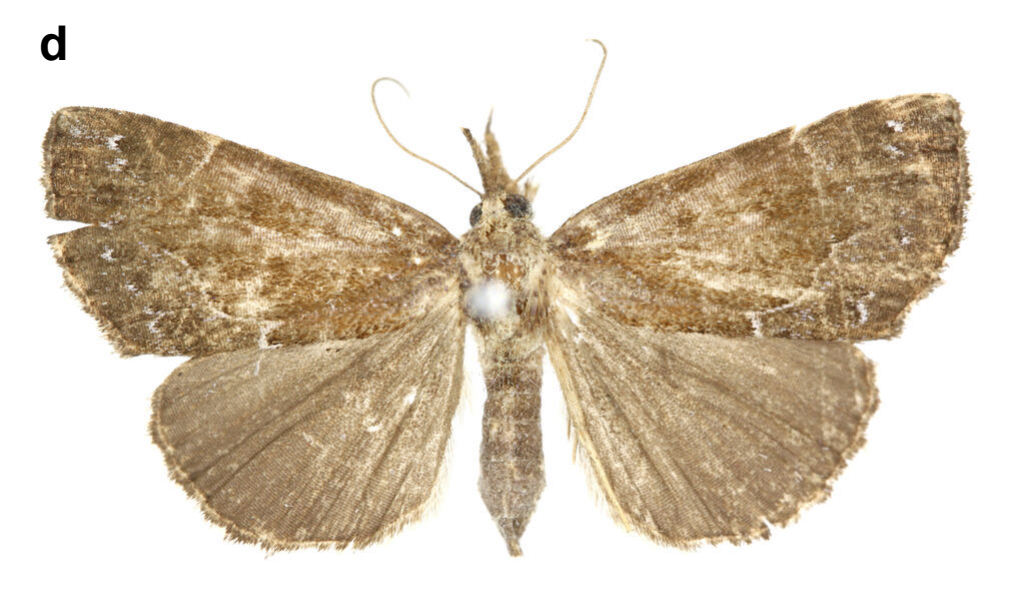
*Hypenarivuligera*, female;

**Figure 5e.**
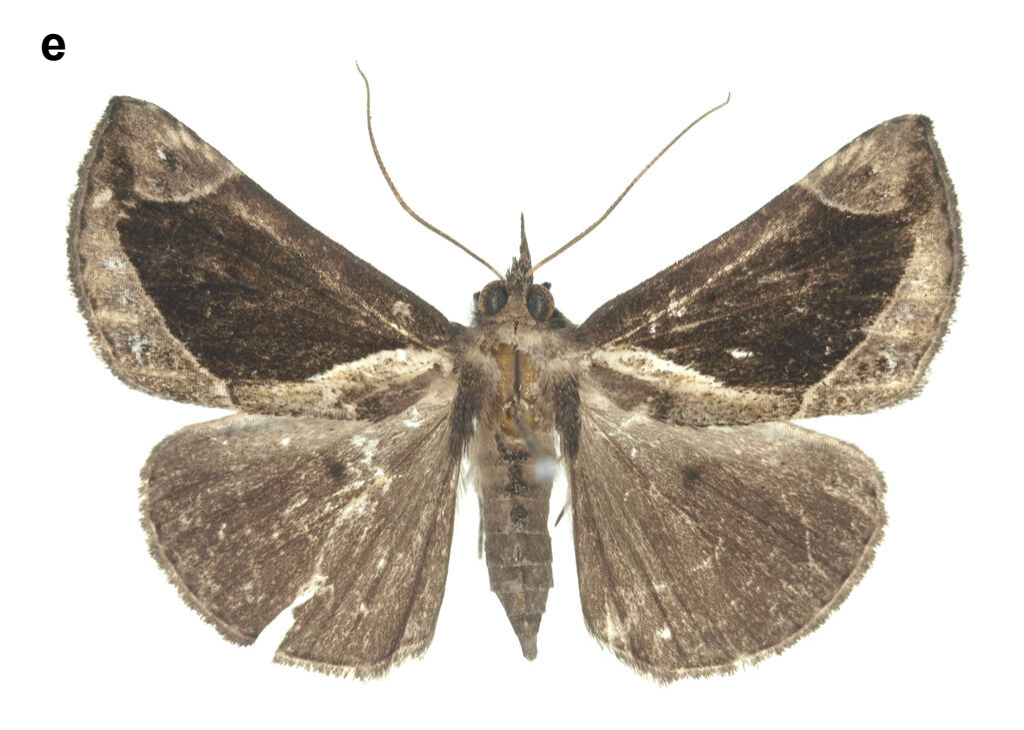
*Hypenaperspicua*, female;

**Figure 5f.**
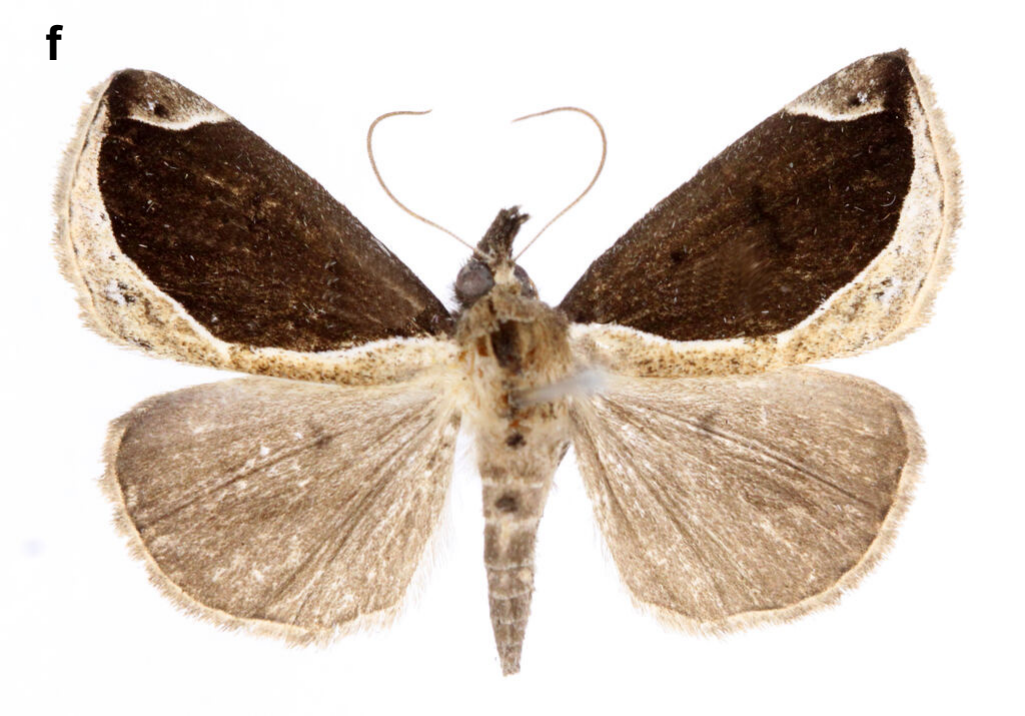
*Hypenabicoloralis*, male.

**Figure 6a.**
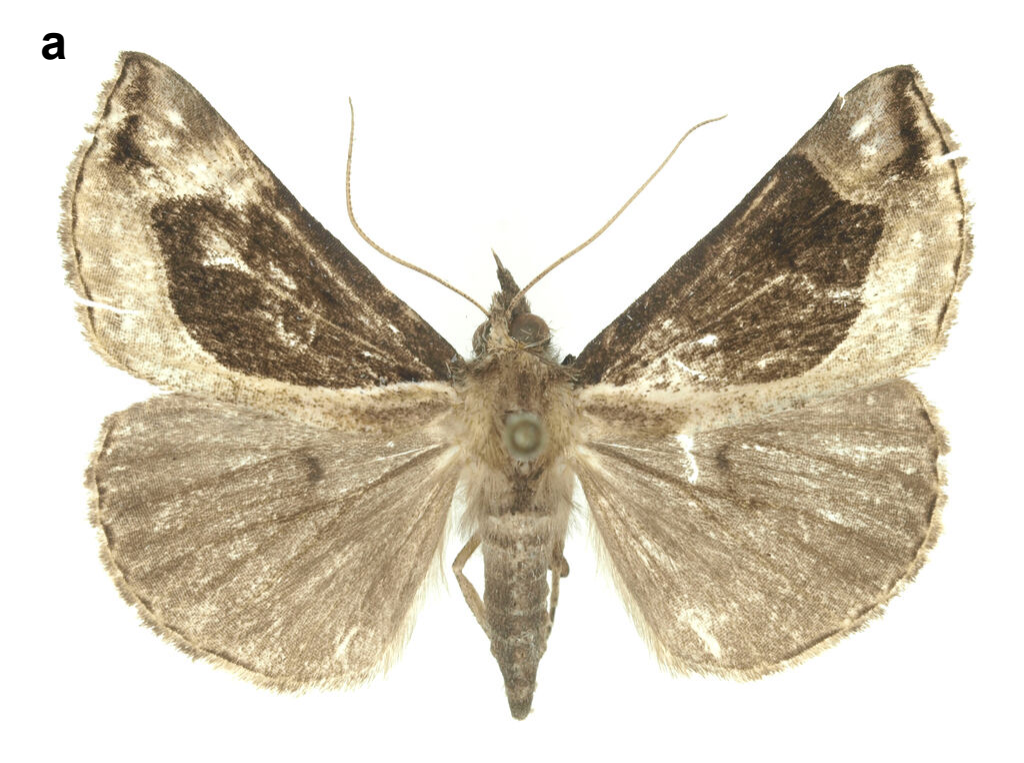
*Hypenamandarina*, male;

**Figure 6b.**
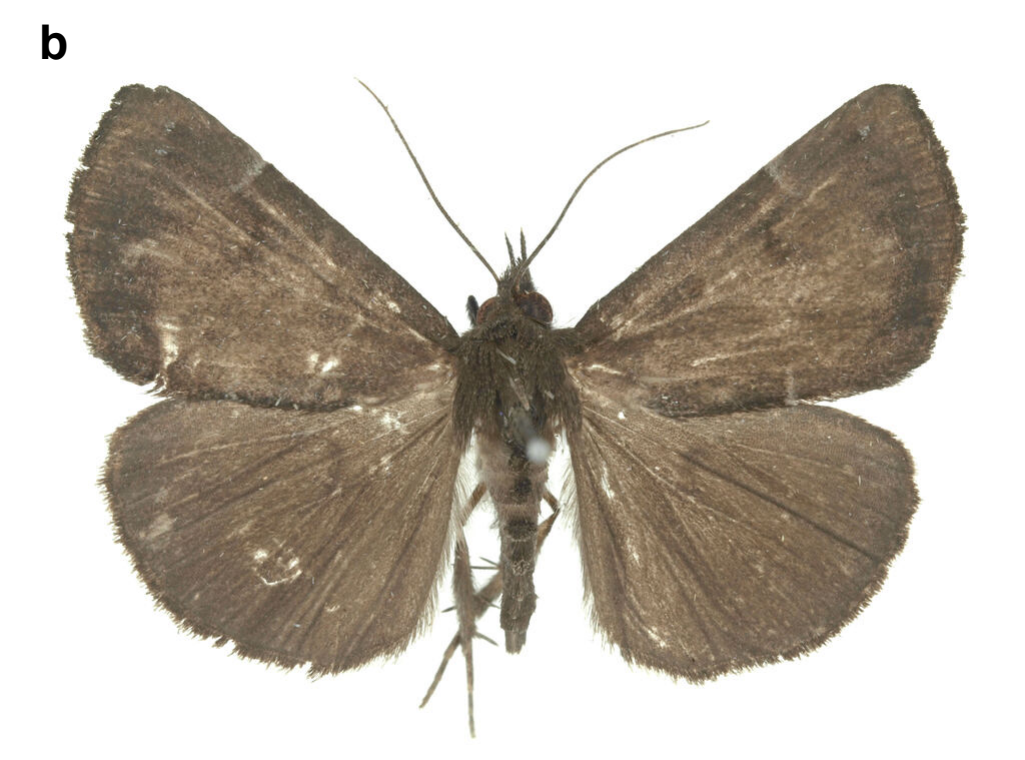
*Hypenamelanica*, male.

**Figure 7a.**
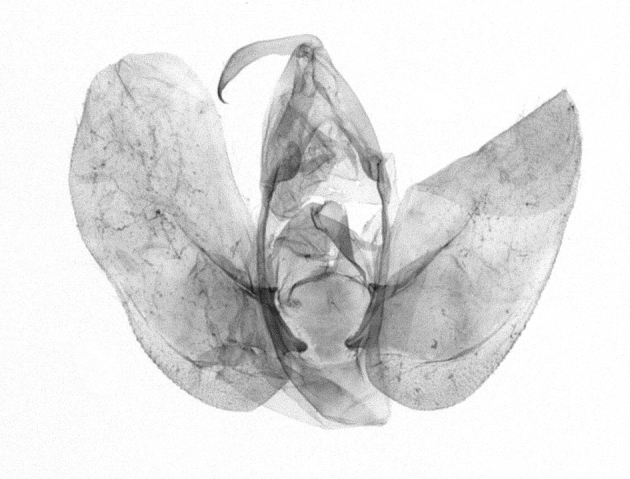
*Hypenasagitta*, male genital capsule;

**Figure 7b.**
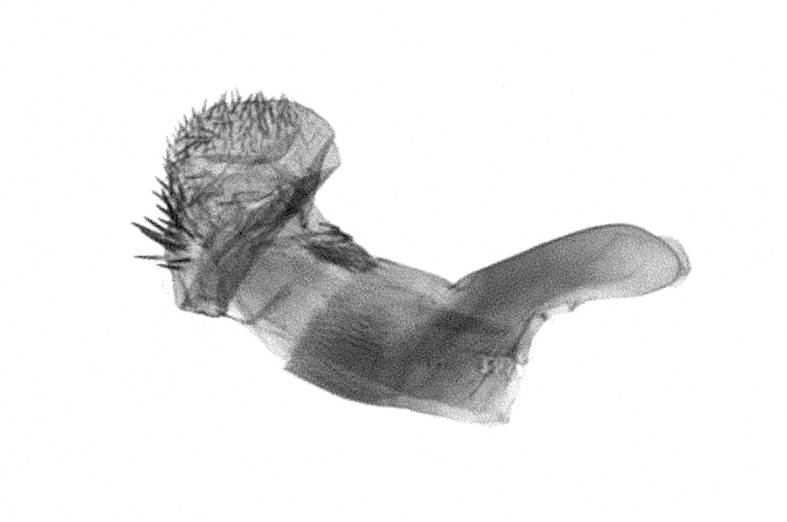
*Hypenasagitta*, aedeagus;

**Figure 7c.**
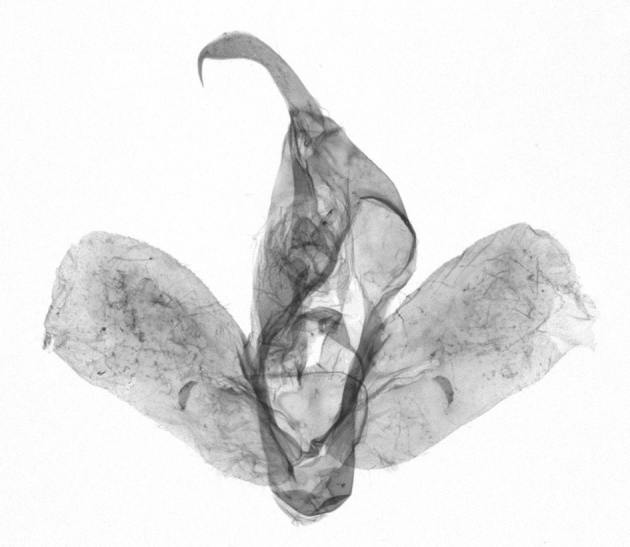
*Hypenaproboscidalis*, male genital capsule;

**Figure 7d.**
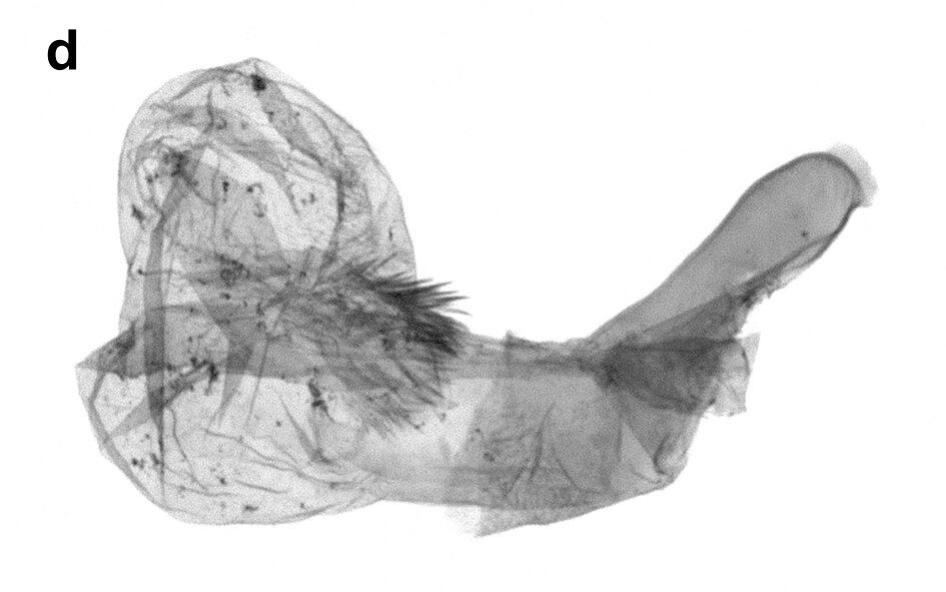
*Hypenaproboscidalis*, aedeagus;

**Figure 7e.**
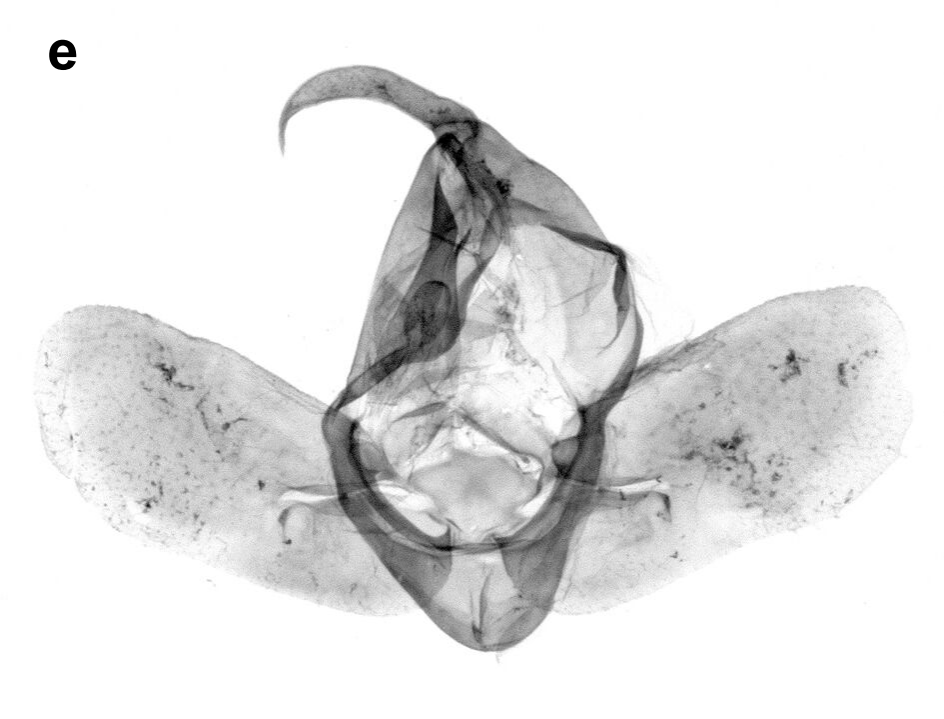
*Hypenatamsi*, male genital capsule;

**Figure 7f.**
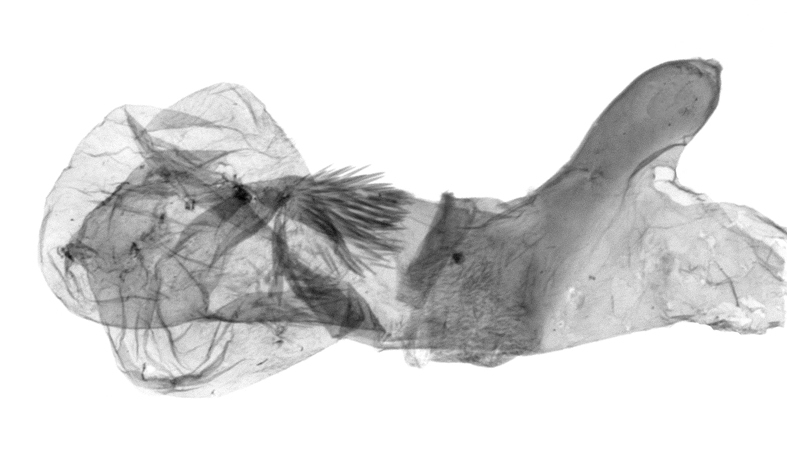
*Hypenatamsi*, aedeagus.

**Figure 8a.**
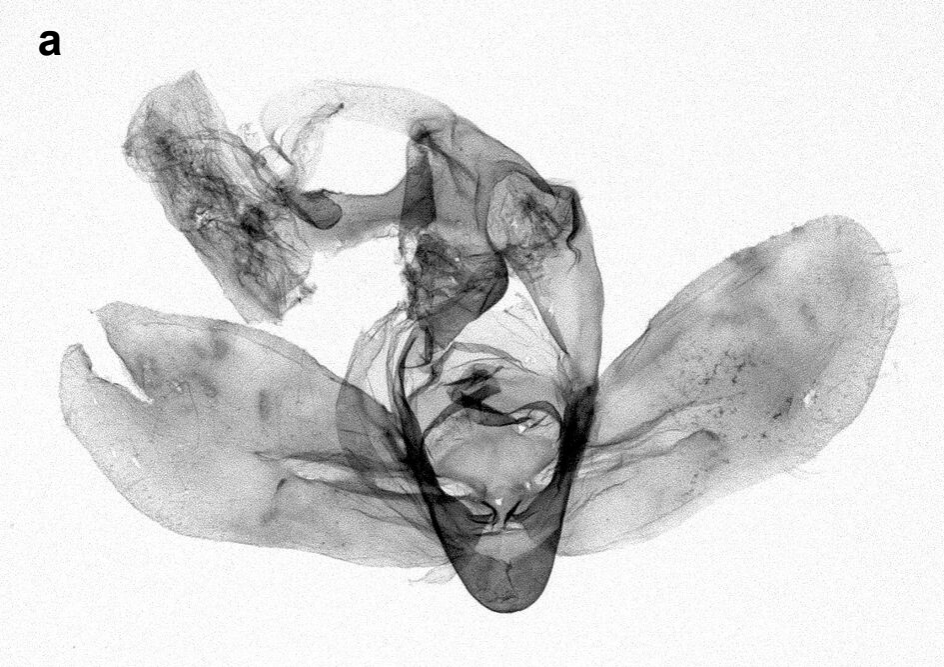
*Hypenapulverulenta*, male genital capsule;

**Figure 8b.**
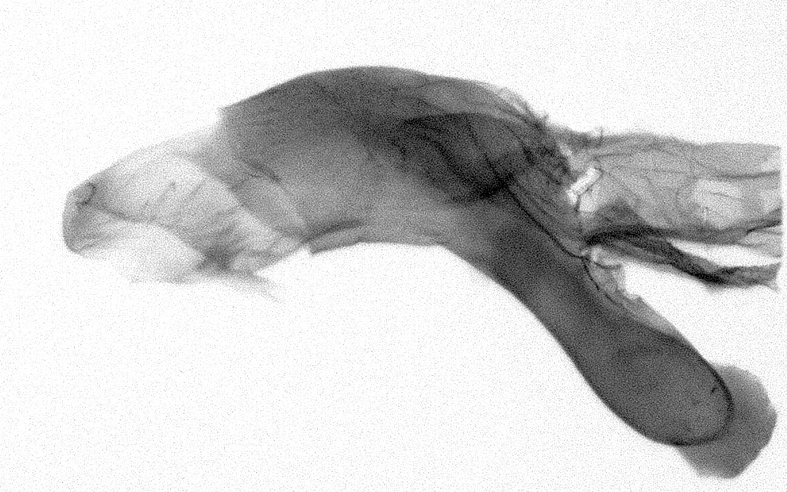
*Hypenapulverulenta*, aedeagus;

**Figure 8c.**
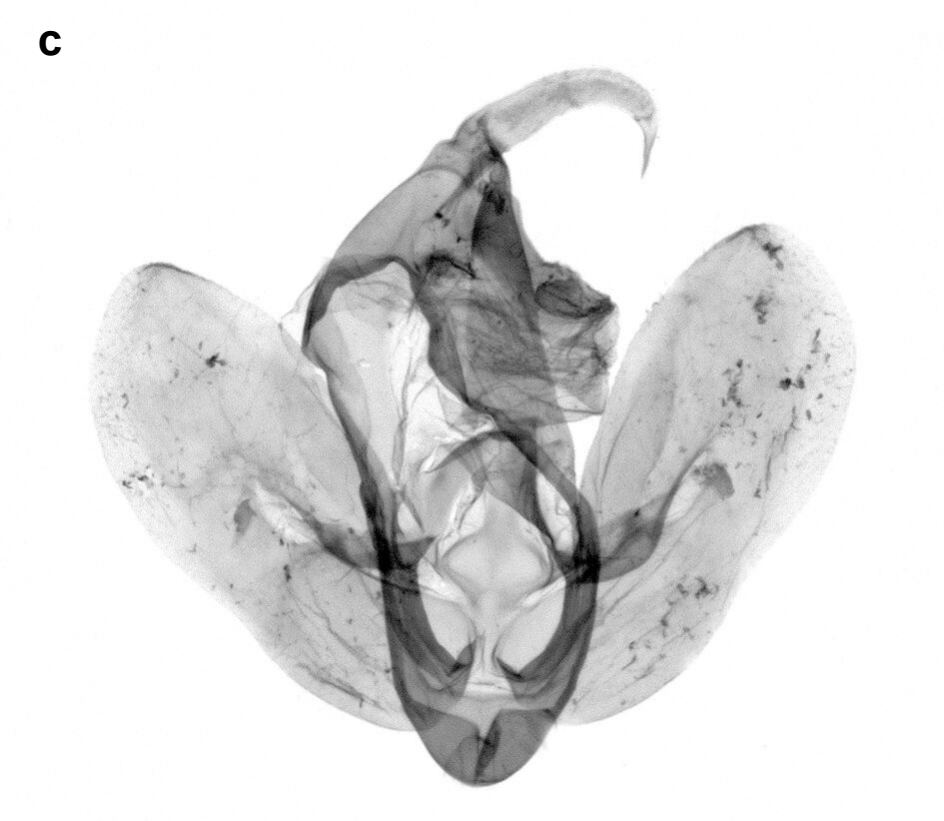
*Hypenaperspicua*, male genital capsule;

**Figure 8d.**
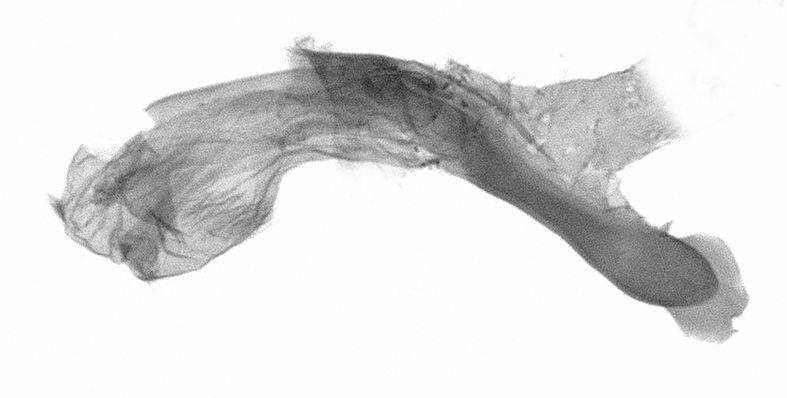
*Hypenaperspicua*, aedeagus;

**Figure 8e.**
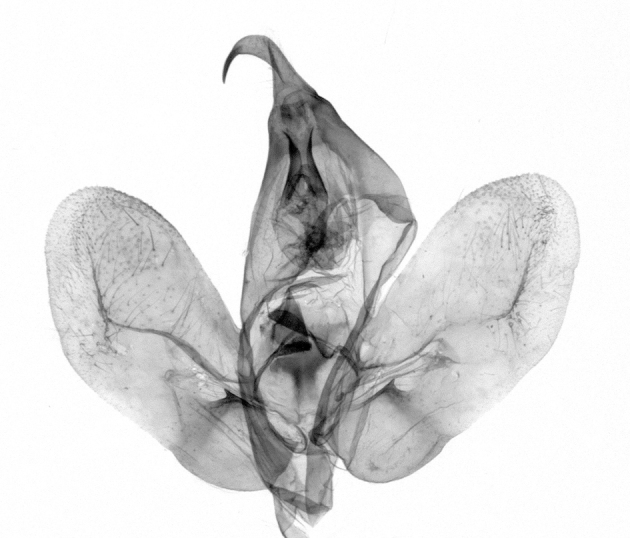
*Hypenamandarina*, male genital capsule;

**Figure 8f.**
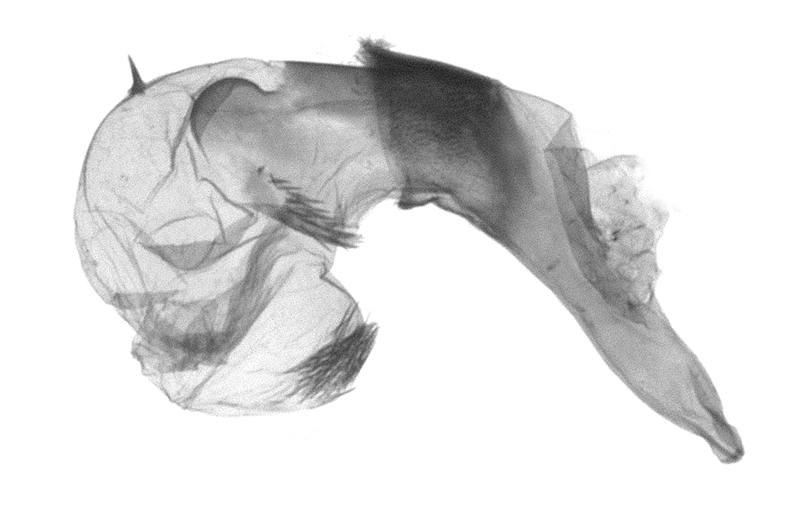
*Hypenamandarina*, aedeagus.

**Figure 9a.**
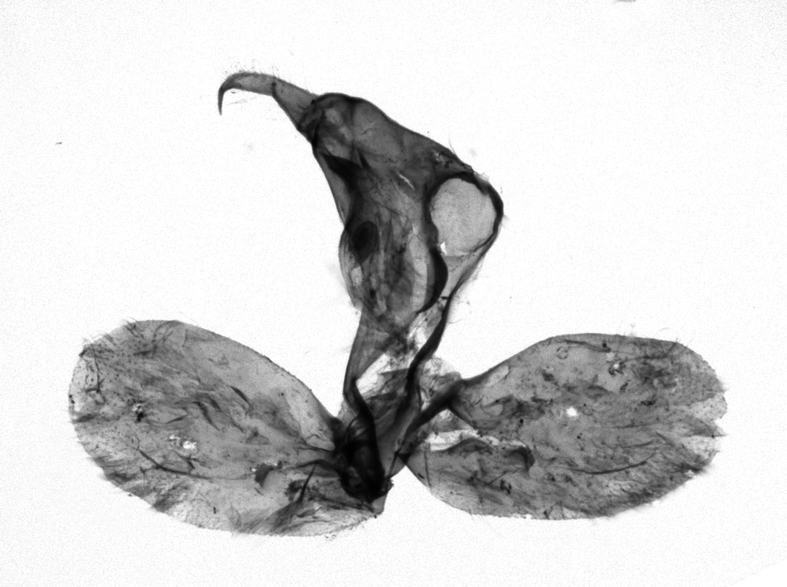
Male genital capsule;

**Figure 9b.**
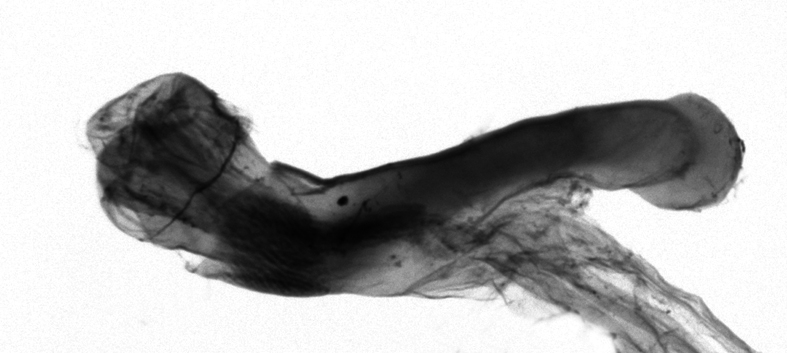
Aedeagus.
