# Supplementary material for: An updated list of the genus Hypena Schrank (Lepidoptera, Erebidae) from Korea with five additional records to the fauna
Source: Biodivers Data J. 2025 May 2;13:e155581. doi: 10.3897/BDJ.13.e155581 (PMC12065010; doi:10.3897/BDJ.13.e155581)
Supplement: Supplementary material 1 — List of the species included in our analyses and information about the sources of materials [file bdj-13-e155581-s001.docx]

Appendix 1. List of the species included in our analyses and information about the sources of materials, including collection locality, voucher, and accession number in GenBank.

| **Family Subfamily** | **Species** | **Collection locality** | **Voucher** | **GenBank accession no.** |
| --- | --- | --- | --- | --- |
| Erebidae | *Hypena squalida* | Korea: Pyeongchang | MNU_H1 | PV274473 |
| Hypeninae | *Hypena stygiana* | Korea: Jecheon | MNU_H3 | PV274475 |
|  | *Hypena nigrobasalis* | Korea: Goesan | MNU_H9 | PV274476 |
|  | *Hypena nigrobasalis* | Korea: Shinan | MNU_H12 | PV274477 |
|  | *Hypena nigrobasalis* | Korea: Jeju | MNU_H13 | PV274478 |
|  | *Hypena proboscidalis* | Korea: Yanggu | MNU_H15 | PV274479 |
|  | *Hypena nigrobasalis* | Korea: Gurye | MNU_H19 | PV274480 |
|  | *Hypena zilla* | Korea: Inje | MNU_H20 | PV274481 |
|  | *Hypena stygiana* | Korea: Gurye | MNU_H21 | PV274482 |
|  | *Hypena tamsi* | Korea: Samcheok | MNU_H29 | PV274484 |
|  | *Hypena obacerralis* | Korea: Sacheon | MNU_H31 | PV274486 |
|  | *Hypena narratalis* | Korea: Pyeongchang | MNU_H32 | PV274487 |
|  | *Hypena perspicua* | Korea: Jeju | MNU_H33 | PV274488 |
|  | *Hypena mandarina* | Korea: Jangseong | MNU_H34 | PV274489 |
|  | *Hypena mandarina* | Korea: Gurye | MNU_H35 | PV274490 |
|  | *Hypena pulverulenta* | Korea: Sejong | MNU_Mi97 | PV274491 |
|  | *Hypena trigonalis* | Japan: Chubu | AYK-04-5173 | KF491804 |
|  | *Hypena obesalis* | Italy | KLM Lep 14607 | OQ181704 |
|  | *Hypena obesalis* | Germany | BC ZSM Lep 22024 | GU686956 |
|  | *Hypena obesalis* | Russia | MM19071 | JF854432 |
|  | *Hypena obesalis* | Russia | MM19072 | JF854433 |
|  | *Hypena rostralis* | Italy | BC ZSM Lep 41890 | KX040092 |
|  | *Hypena rostralis* | Germany: Bavaria | BC ZSM Lep 22376 | GU654962 |
|  | *Hypena rostralis* | Germany: Bavaria | BC ZSM Lep 22299 | GU686892 |
|  | *Hypena rostralis* | Finland | MM15854 | HM876629 |
|  | *Hypena tristalis* | Japan | AYK-06-7270 | JN273650 |
|  | *Hypena tristalis* | Japan | AYK-06-7271 | JN273651 |
|  | *Hypena zilla* | Japan | AYK-04-1015-17 | KF491598 |
|  | *Hypena proboscidalis* | Italy: Piemonte | TLMF Lep 27911 | OQ181490 |
|  | *Hypena proboscidalis* | Italy: Basilicata | BC ZSM Lep 60926 | KX047464 |
|  | *Hypena claripennis* |  | 0728 | MW085439 |
|  | *Hypena quinqualis* |  | 0581 | MW085306 |
|  | *Hypena quinqualis* |  | 1594 | KT988756 |
| Erebidae | *Paracolax tristalis* | Italy: Piedmont | KLMLep15853 | OQ183068 |
| Herminiinae | *Zanclognatha lunalis* | Germany: Bavaria | BC ZSM Lep 61298 | KX044509 |
